# Agp2, a Member of the Yeast Amino Acid Permease Family, Positively Regulates Polyamine Transport at the Transcriptional Level

**DOI:** 10.1371/journal.pone.0065717

**Published:** 2013-06-03

**Authors:** Mustapha Aouida, Marta Rubio Texeira, Johan M. Thevelein, Richard Poulin, Dindial Ramotar

**Affiliations:** 1 Center for Desert Agriculture, King Abdullah University of Science and Technology, Thuwal, Saudi Arabia; 2 Laboratory of Molecular Cell Biology, Institute of Botany and Microbiology, Leuven, Belgium; 3 Department of Molecular Microbiology, Flanders Institute of Biotechnology, Flanders, Belgium; 4 Department of Molecular Biology, Medical Biochemistry and Pathology, Laval University Quebec, Canada; 5 Maisonneuve-Rosemont Hospital, Research Center, University of Montreal, Immunology and Oncology, Montreal, Canada; Université de Sherbrooke, Medicine, Canada

## Abstract

Agp2 is a plasma membrane protein of the *Saccharomyces cerevisiae* amino acid transporter family, involved in high-affinity uptake of various substrates including L-carnitine and polyamines. The discovery of two high affinity polyamine permeases, Dur3 and Sam3, prompted us to investigate whether Agp2 directly transports polyamines or acts instead as a regulator. Herein, we show that neither *dur3Δ* nor *sam3Δ* single mutant is defective in polyamine transport, while the *dur3Δ sam3Δ* double mutant exhibits a sharp decrease in polyamine uptake and an increased resistance to polyamine toxicity similar to the *agp2Δ* mutant. Studies of Agp2 localization indicate that in the double mutant *dur3Δ sam3Δ*, Agp2-GFP remains plasma membrane-localized, even though transport of polyamines is strongly reduced. We further demonstrate that Agp2 controls the expression of several transporter genes including *DUR3* and *SAM3*, the carnitine transporter *HNM1* and several hexose, nucleoside and vitamin permease genes, in addition to *SKY1* encoding a SR kinase that positively regulates low-affinity polyamine uptake. Furthermore, gene expression analysis clearly suggests that Agp2 is a strong positive regulator of additional biological processes. Collectively, our data suggest that Agp2 might respond to environmental cues and thus regulate the expression of several genes including those involved in polyamine transport.

## Introduction

Agp2 is a 67.2-kDa plasma membrane protein that contains 12 potential transmembrane domains and belongs to the yeast *Saccharomyces cerevisiae* amino acid permease family that also includes many other members such as Put4, Alp1, Lyp1, Can1 and Gap1 [1], [2]. Agp2 was initially shown to be involved in the plasma membrane transport of L-carnitine into yeast cells [3]. L-carnitine serves as a carrier to transport acetyl-CoA generated by fatty acid β-oxidation via the peroxisomal-mitochondrial carnitine acetyltransferase (Cat2) shuttle for complete oxidation by the Krebs cycle [3]. *AGP2* was subsequently shown to be negatively regulated by the high osmolarity glycerol response 1 gene (*HOG1*), encoding a MAP kinase, whereby under high osmolarity conditions *AGP2* expression was downregulated [4]. Additional studies identified a function for Agp2 as a low-affinity, non-specific amino acid permease in poor nutrient conditions [1]. However, its low level of expression, its low affinity and specificity as well as its redundant function with that of several more active amino acid permeases, including Gap1, raised important questions about the relevance of a direct role for Agp2 in amino acid transport.

Using a high-throughput screen, we later discovered that single deletion of *AGP2*, similar to four other genes (*PTK2*, *SKY1*, *FES1*, and *BRP1*), causes enhanced resistance to the anticancer drug bleomycin-A5 (BLM-A5), a spermidine-conjugated bleomycin species which acts via DNA damage induction [5]. We further reported that Agp2 mediates high-affinity polyamine transport, using spermine and spermidine with a higher affinity than putrescine as substrates, a specificity pattern corresponding to that observed in wild-type cells [6]. This is supported by the fact that *agp2*-defective mutants exhibit a dramatically reduced initial velocity of spermidine and spermine uptake, and a very strong resistance to the toxicity of spermine and its analogs [6]. Introduction of a plasmid overexpressing *AGP2* restored polyamine toxicity to the *agp2*-defective mutants [6]. Based on these observations, we reached the conclusion that *AGP2*, which has all the conserved features of amino acid permeases of its family, was the first eukaryotic gene identified as encoding a polyamine permease in yeast [6]. In fact, van Roermund *et al.* had identified Agp2 as a carnitine permease based on the same type of evidence as that used by our group to assign a direct role in polyamine transport to its gene product [3]. However, we were puzzled by preliminary observations indicating that addition of high concentrations of L-carnitine (up to a 1000-fold excess) did not compete for the uptake of radiolabeled polyamines nor prevented spermine toxicity [6] (see below). To account for these observations, we proposed various hypotheses for the actual transport function(s) of Agp2 that include (A) that Agp2 possesses two independent binding sites, specific for L-carnitine or polyamines, respectively, and thus acts as a polyspecific transporter accepting substrates with different sizes and molecular structures, (B) a role of Agp2 as a non transporting transceptor, *i.e.*, a transporter which has lost the ability to transport any ligands and that may thus act as a sensor to regulate the expression of L-carnitine and polyamine permeases encoded by other, as yet undescribed genes, and (C) acting as a transporting transceptor of polyamines in a signal transduction pathway for the expression of other transporters [6], [7].

In *S. cerevisiae*, several non transporting transceptors (or sensors) have been previously described such as Ssy1, a plasma membrane protein belonging like *AGP2* to the yeast amino acid permease family, but which is endowed with no or only limited transport function [8]. Ssy1 senses amino acid availability by direct interaction with extracellular amino acids and triggers the expression of several downstream target genes, that encode amino acid permeases such as *AGP1*, *BAP2*, *BAP3*, *DIP5*, and *TAT1* via the formation of an intermediary complex, the SPS sensor, with the plasma membrane proteins Ptr3 and Ssy5 [8], [9]. In this sensory system, Ptr3 becomes hyperphosphorylated allowing the activation of the protease Ssy5, which cleaves the latent form of the two homologous zinc-finger transcription factors, Stp1 and Stp2, present in the cytosol, which then enter the nucleus and induce the Ssy1-targets [10]. Other examples include transporting transceptors like Gap1 and Mep2, which not only act as a general amino acid transporter and ammonium permease, respectively, but also as a nitrogen sensor that signals through the rapid activation of protein kinase A (PKA) targets [11], [12]. Thus, in view of the increasing number of sensors that are currently being identified, and since a permease with independent binding sites for similar substrates (*i.e.* model A above) is highly implausible, we assess here the hypothesis whether Agp2 may instead possess regulatory function. A regulatory model for Agp2 became even more plausible with the report by Uemura *et al.*, that Dur3 and Sam3 are the major high-affinity permeases accounting for polyamine uptake in yeast [13]. This is supported by the fact that *agp2*-defective mutants exhibit a dramatically reduced initial velocity of spermidine and spermine uptake, and a very strong resistance to the toxicity of spermine and its analogs [6]. In this study, we now document that the *agp2Δ* single mutant and the *dur3Δ sam3Δ* double mutant display similar phenotypes towards polyamines, in contrast to the *dur3Δ* or *sam3Δ* single mutants. Using a variety of approaches that included RT-PCR, microarray and gene expression profile analyzer (GeXP), we also demonstrate for the first time that in cells lacking a functional *AGP2* gene nearly 172 genes are downregulated as compared to the parent. Amongst the downregulated genes, many encode membrane transporters and these include *DUR3*, *SAM3*, *HNM1*, *TPO2* and *HXT3*. Moreover, we have identified the expression of *SKY1*, a SR kinase that positively regulates low-affinity polyamine transport via its indirect effect on membrane potential, as a target of Agp2 [13], [14], [15], [16], [17], [18]. We propose that Agp2 mainly acts as a regulator of polyamine transport as well as other specific biological functions.

## Materials and Methods

### Strains, Media, Transformation, and Reagents

The *S. cerevisiae* strains used in the present study are listed in Table 1. Yeast cells were grown at 30***°***C in either YPD [1% (w/v) yeast extract, 2% (w/v) peptone, 2% (w/v) dextrose] or minimal synthetic (SD: 0.65% yeast nitrogen base without amino acids, 2% dextrose, 0.17% dropout mix) medium used for transformation [19], [20]. Single and double gene deletion mutants were created by standard one-step gene targeting in the parent strain BY4741 and verified by PCR analysis and cells were transformed by the lithium acetate method [6], [21]. The *dur3Δ::KANMX sam3Δ::HIS3* double mutant was created from the *dur3Δ::KANMX* single mutant using the following primers SAM3-UNI-KO-F1: CTGAAATATCAAGATGGATATACTCAAGAGGGGAAATGAATCGGACCAGATTGTAC TGAGAGTGCAC and SAM3-UNI-KO-R1:CTGTAGATTTTGTAATAGAATGGCTTAGA AGCAATGAGTTGTTCCCTGTGCGGTATTTCACACCGC. The *agp2Δ::URA3 dur3Δ::KANMX sam3Δ::HIS3* triple mutant was derived from the *dur3Δ::KANMX sam3Δ::HIS3* double mutant by replacing the entire *AGP2* gene with the selection cassette *URA3*. [^14^C]-Spermidine trihydrochloride (112 mCi/mmol) and L-[methyl-^14^C]-carnitine were obtained from Amersham Biosciences (Eugene, OR, USA). For the localization of Agp2-GFP, a previously described multicopy URA3 plasmid expressing C-terminally GFP tagged Agp2 under its own promoter, pAGP2-GFP, was employed [6]. For colocalization with the endosomal domain FYVE a plasmid expressing a DsRed tagged version (pTPQ127) was employed [22]. For colocalization of Agp2-GFP with Sec66mRFP the deletion collection BY4741 *agp2Δ::KanMX4* strain was crossed to BY4742 containing *Sec66-mRFP::KanMX6* [23]. A segregant *agp2Δ::KanMX4 Sec66-mRFP::KanMX6* (MRT 299) was selected. Similarly, BY4741 *agp2Δ::KanMX4* was crossed to BY4742 containing *agp2Δ::KanMX4* to obtain *agp2Δ::KanMX4 end3Δ::KanMX4* double mutant strain (MRT361). Both segregants were subsequently transformed with pAGP2-GFP.

**Table 1:** Strains used in this study.

Strains	*Genotypes*	*Sources*
BY4741(parent)	*MATa his3Δ leu2Δ met15Δ ura3Δ*	Research Genetics
BY 4741(*agp2Δ*)	Isogenic to BY4741, except *agp2 Δ::URA3*	This study
**BY 4741(** ***agp2Δ*** **)**	Isogenic to BY4741, except *agp2Δ::KANMX*	Research Genetics
BY 4741(*dur3Δ*)	Isogenic to BY4741, except *dur3Δ:: KANMX*	Research Genetics
BY 4741(*sam3Δ*)	Isogenic to BY4741, except *sam3Δ::HIS3*	Research Genetics
BY 4741(*hnm1Δ*)	Isogenic to BY4741, except *hnm1Δ::KANMX*	Research Genetics
BY 4741(*sky1Δ*)	Isogenic to BY4741, except *sky1Δ::KANMX*	Research Genetics
BY 4741(*sam3Δ dur3Δ*)	Isogenic to BY4741, except *dur3Δ::KANMX sam3Δ::HIS3*	This study
*agp2Δ dur3Δ sam3Δ*	Isogenic to BY4741, except *agp2Δ::URA3 dur3Δ::KANMX sam3Δ::HIS3*	This study
*agp2Δ end3Δ*	Isogenic to BY4741, except *agp2Δ::URA3 end3Δ::KANMX*	This study
*agp2Δ sky1Δ*	Isogenic to BY4741, except *agp2Δ::URA3 sky1Δ::KANMX*	This study
*agp2Δ sok2Δ*	Isogenic to BY4741, except *agp2Δ::URA3 sok2Δ::KANMX*	This study

### Spot Test Analysis of Cell Growth

Standard spot tests were performed as previously described [24].

### Microscopy

For fluorescent localization studies, imaging was carried out with an Olympus FV1000 confocal laser scanning biological microscope, and images were processed with the accompanying software, FV10-ASW 2.0. Cells were in some cases stained with DAPI (2.5 µg/ml final concentration; Roche) for simultaneous localization of nuclei.

### RNA extraction and reverse transcription (RT)-PCR analysis

Total RNA was prepared using the RiboPure-Yeast extraction kit (Ambion) from 3 ml of an overnight suspension culture and treated with TURBO DNA-free kit (Ambion) to eliminate genomic DNA contamination. Total RNA was quantified by measuring *A*_260_ spectrophotometrically. The cDNA was synthesized using M-MLV reverse transcriptase from Invitrogen. The PCR primers used to amplify the target genes are listed in Table 2. The PCR program was 2 min at 95°C followed by 25 cycles of 1 min of denaturation at 94°C, 2 min of annealing at different temperature dependent of the gene fragment (e.g. 54°C for *ACT1* fragment) and 4 min of primer extension at 72°C. This was followed by extension at 72°C for 7 min. The identity of PCR products was verified on a 1% agarose gel.

**Table 2:** List of RT-PCR used for monitoring Agp2 target and non-target genes.

Gene	Primer sequence (5′- to -3′)	Size of PCR product (pb)
*AGP2*	F:GTCATTGGTACGGCGCTATTCGTGGCGATCGG	572
	R:GCGAGAAACCCCTGGAAGTAGCCCGAAGACT	
*DUR3*	F:AGGCGCTGGGTACGCTATTGTATTGGGCCTAGGGGCCG	690
	R:CCGTCTACTGGATGCCTCTTGGCGGCTTCACGAACT	
*SAM3*	F:GCCGGAGGGGGGCCTAACTCTACTGGTTATATTGGC	604
	R:GGGCGAAAAGCCAAGTGAAGACCTCGTCCTCCT	
*HNM1*	F:CCGCATATCCGCACGCCGGTGGTCAGTTTTGGTGG	643
	R:GGGGCGCCTGTTGTAGAAGACAAGACAGCGTCC	
*SKY1*	F:ATCGCATGTGTCCATCCAATCGGATTCGGGGCCAC	712
	R:CGCGGTAATATCAGAAGACCGCGAAACGTGCCTCT	
*GAP1*	F:GCCATCGGTGGTGCCATCGGTACTGGTCTGCTGG	527
	R:GCAAAGGCACCAGGATCATGCCAGTACTTGCCCCC	
*FIG1*	F:GGGCTTGGAGGAAGTCATTATAAGATCCGG	520
	R:CCAAGCCATTACTGCTGCCTTCTTGCCCT	
*TPO2*	F:ACGGTTCTGCTTGTATCAGTGGTGGGTTGGG	410
	R:GACCCAGAAAATCAGGTCCATACGTCCGGT	
*HXT3*	F:TCCGGTTTGGGTGTTGGTGGTATTGCCG	411
	R:CTCACCCCATGATGCTGAACCAGCAGCTCT	
*ACT1*	F:GTTTTGCCGGTGACGACGCTCCTCGTGCTG	357
	R:CGGCTTGGATGGAAACGTAGAAGGCTGGAACG	
*AOT3*	F:ATTGAGACGCTCCCCCAGCAGGGTTCG	510
	R:CCAGCCCGCAAGGAAGAACCCAATGACAT	

### Microarray analysis

Total RNA was isolated from exponentially growing cultures of the parent and the *agp2Δ* mutant using the RiboPure kit above. RNA samples were then hybridized to a GeneChip Yeast Genome 2.0 array that includes approximately 5,744 probe sets for 5,841 of the 5,845 genes in *S. cerevisiae* (Affymetrix, Santa Clara, CA). Probe sets on the array include 11 oligonucleotide pairs to detect each transcript. Microarray was performed and analyzed using the services of Genome Quebec (The Innovation Centre, McGill University). Densitometry readings were exported to a project file and statistical analysis of the datasets was performed using the FlexArray® program developed by Genome Quebec which embeds the R® statistical software. Raw data were first normalized using robust multi-array averages and statistical comparisons between the two sets of RNA-DNA chip hybrids were performed with two-sample Bayesian *t*-tests including a Bonferroni correction [25], [26]. Genes that were significantly downregulated by ≥2-fold or upregulated by ≥2-fold can be found in Supplemental Tables S1 and S2, respectively, which include the gene name and a brief functional description, as well as the *t*-test and *P* values. The threshold for significant differences between the parent and *agp2Δ* mutant was set at *P*<0.001 for better stringency.

### Multiplex analysis by GeXP system

The procedure for sample preparation and subsequent gene expression analysis was performed according to manufacturer's instructions using the GenomeLab™ Gene eXpression Profiler (GeXP; Beckman Coulter, Canada) and as previously described [27]. Briefly, the primers used for GeXP are listed in Table 3 and were designed using the eXpress Designer module of the express Profiler software (Beckman Coulter). A total of 25 ng RNA was used in a 20 µL reaction volume for RT. Kanamycin RNA, an internal positive control was included. The RT reaction was performed using the GenomeLab ™ GeXP Start Kit (Beckman Coulter) under the conditions: 1 min at 48°C, 60 min at 42°C, 5 min at 95°C, hold at 4°C, in a thermal cycler. After the RT, a PCR was performed under the conditions: 10 min at 95°C, followed by 35 cycles of 30 s at 94°C, 30 s at 55°C, and 1 min at 70°C. The data was normalized to Kanamycin before being expressed as a log 2 ratio.

**Table 3:** List of multiplex primers used for GeXP analysis.

Gene	Left Sequence w/Universals	Right Sequence w/Universals
FES1	AGGTGACACTATAGAATAGCTGTGCAAAACAACTTGGA	GTACGACTCACTATAGGGACGAGTGGCTTTGTCTTGTCA
CTK1	AGGTGACACTATAGAATAGGCCCAAATCTGTTGAGAAA	GTACGACTCACTATAGGGATTCCTGGCCTTTGTGGTTAT
APN1	AGGTGACACTATAGAATACAAGAAAGTGGGTTTCTCCG	GTACGACTCACTATAGGGAAGTATTGGCCATGCGGTAAG
SAM3	AGGTGACACTATAGAATACAGAGCTTTCCTGCCAATTC	GTACGACTCACTATAGGGAAAAGAAATCAGCCAAGCCAA
TPO2	AGGTGACACTATAGAATACTCATGGGTTCGTTGGAGTT	GTACGACTCACTATAGGGACGGCTTCCATACCTACATGG
BRP1	AGGTGACACTATAGAATACGGTAATTATCGCCGGATCT	GTACGACTCACTATAGGGAAACAAGCTTCCTGAAACGGA
AGP2	AGGTGACACTATAGAATACCTTGCACTCGGCCTATTTA	GTACGACTCACTATAGGGACGAAGACTTCCCCACATCAT
SOK2	AGGTGACACTATAGAATATGATATCAAGTCCAACCGCA	GTACGACTCACTATAGGGAGGTAGGATACGGGCATCAGA
AGP1	AGGTGACACTATAGAATACAAGGTCTGGCACAAGGATT	GTACGACTCACTATAGGGAGCAACGACCCTTTTCCAATA
NHX1	AGGTGACACTATAGAATAAACCCGGCAAGGTCTTCTAT	GTACGACTCACTATAGGGAGTGATTGCGTAGCCGAATCT
OPT2	AGGTGACACTATAGAATATCAACGGGCTTGGTTTTATC	GTACGACTCACTATAGGGACCTGGAGCATAGCCGAAATA
SGS1	AGGTGACACTATAGAATAAAGACAAATTCGCCACCAAC	GTACGACTCACTATAGGGATTGATTCGATGTGGCAGTGT
SKY1	AGGTGACACTATAGAATACGCTCATTTGGCTGACACTA	GTACGACTCACTATAGGGAACCGTTGGCACTAGAGGATG
FIG1	AGGTGACACTATAGAATAGATCCGGTTACATGGGTGTT	GTACGACTCACTATAGGGAGACAACGCTTGATTGGGTTT
ABF1	AGGTGACACTATAGAATAATGCTGCATCCTCAGAAACC	GTACGACTCACTATAGGGATGTTCGCCAGATGGTATTCA
ZUO1	AGGTGACACTATAGAATATCGAACCGGTTGGTAAGTTC	GTACGACTCACTATAGGGATCGGCAGTTTTCCAGTCTCT
PDR13	AGGTGACACTATAGAATATCCGCTTTATCCTATGTCGG	GTACGACTCACTATAGGGATCCTTACCTTCGCCTCTTGA
PTK2	AGGTGACACTATAGAATAGCAGTGGTAGCGGTGGTAAT	GTACGACTCACTATAGGGAGAGGCAATGCCAGTACCTGT
RAD50	AGGTGACACTATAGAATACCAACAGCAAGGGAGGAGTA	GTACGACTCACTATAGGGAGAGACCGGGTGGACAAAGTA
HNM1	AGGTGACACTATAGAATAAATCAGGTGCGATTTTCCTG	GTACGACTCACTATAGGGAGCCGTACTGGAAGCCAAATA
RRD1	AGGTGACACTATAGAATAAGTCTCTGGTGCGCGAGTAT	GTACGACTCACTATAGGGATGTTGACCCAGGGAAAGAAG
RTS1	AGGTGACACTATAGAATATTCTTCCAGCAGCAGTAGCA	GTACGACTCACTATAGGGATACTGCCCCCAGCAAAGTAG
DUR3	AGGTGACACTATAGAATACAGACGGTATATTTGGCGGT	GTACGACTCACTATAGGGACGATAGTGTTCATCCCGGTT
ACT1	AGGTGACACTATAGAATATTGGCCGGTAGAGATTTGAC	GTACGACTCACTATAGGGACCCAAAACAGAAGGATGGAA
Kan®	AGGTGACACTATAGAATAATCATCAGCATTGCATTCGATTCCTGTTTG	GTACGACTCACTATAGGGAATTCCGACTCGTCCAACATC

### Ontological Analysis of the Effects of AGP2 Inactivation on Gene Expression

Considering the large number of genes whose expression was found to be strongly affected upon *AGP2* gene inactivation, we carried out a detailed analysis of the ontologic relationships between the genes found to be either downregulated or upregulated in *agp2* Δ mutants from the Affymetrix microarray experiments. For that purpose, we relied on the ***G*** *ene* ***O*** *ntology en* ***RI*** *chment ana* ***L*** *ysis and visua* ***L*** *iz* ***A*** *tion* (GOrilla) application (http://cbl-gorilla.cs.technion.ac.il) [28] which presents the advantage of rigorous and highly reliable statistical criteria for gene ontology (GO) group assignments and graphic interface.

### Determination of Spermidine and Carnitine Uptake

Prior to polyamine or carnitine uptake assay, cells were grown to the mid-logarithmic phase, washed three times in uptake buffer A (50 mM sodium citrate, pH 5.5, 2% D-glucose), and resuspended in 100 µl of the same buffer at 2×10^7^ cells/ml. The uptake assay was initiated by the addition of either [^14^C]-spermidine or L-[methyl-^14^C]-carnitine hydrochloride followed by incubation in a water bath at 30°C with shaking. The reaction was stopped at predetermined intervals by adding 1 ml of ice-cold uptake buffer. Cells were washed three times with uptake buffer and resuspended in 100 µl of this buffer. Five ml of scintillation mixture (Amersham Biosciences) were added to each sample, and the retained radioactivity was determined by liquid scintillation spectrometry.

### Other Statistical Analysis

Results are expressed as the mean ±SD from three separate experiments with duplicate or triplicate determinations for each experiment.

## Results

### Simultaneous null mutation of SAM3 and DUR3 is needed to mimic the defective polyamine transport phenotype of agp2Δ cells

In 2007, Dur3 and Sam3, belonging to the sodium solute symport family and the amino acid/polyamine/organocation (APC) family, respectively, were reported as the main high-affinity polyamine permeases in *S. cerevisiae* [13], [29]. Although Dur3 and Sam3 virtually account for the total high-affinity influx of polyamines, all previous evidence along with its permease-like structure, point to Agp2 as a major factor promoting both L-carnitine and polyamine transport [3], [6]. In spite of its dual effect on L-carnitine and polyamine transport, a 1000-fold excess of L-carnitine did not compete with the cytotoxic effect of spermine on a wild type yeast strain (Fig. S1). This excludes that Agp2 acts as a transporter with a single binding site for L-carnitine and polyamines, a possibility already considered unlikely in terms of the structure of H^+^-coupled symporters, which normally bear a single binding site for related substrates [2], [30]. An alternative possibility is that Agp2 harbors a sensing function similar to that of Ssy1, which would regulate the expression of L-carnitine and polyamine transporters encoded by other distinct genes, without directly acting as a major influx pathway for substrate entry [30].

**Figure 1:**
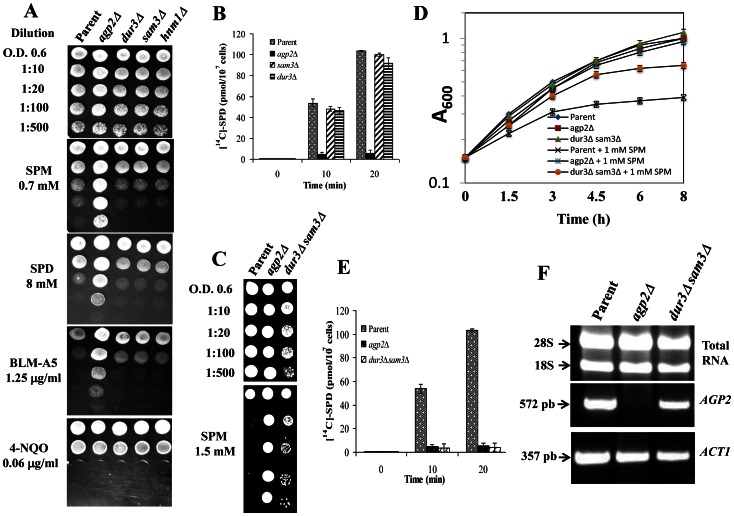
Cellular responses of the parent and the isogenic sam3Δ and dur3Δ single and double mutants towards polyamine. A and C) spot test analysis. Exponentially growing cells of OD 600 ∼0.6 were serially diluted as indicated and 5 µl spotted onto YPD agar containing various concentrations of spermine (SPM), spermidine (SPD), bleomycin-A5 (BLM), and the control drug 4-nitroquinoline-1-oxide (4-NQO). Plates were photographed after 48 h of incubation at 30°C. B and E) [^14^C]-spermidine uptake. Yeast strains were incubated with 10 µM of labelled [^14^C]-spermidine and samples withdrawn to monitor the uptake level. The data is the result of three independent analyses. D) *dur3Δ sam3Δ* double mutant is less tolerant to SPM as compared to the *agp2Δ* mutant in liquid media. Overnight cultures were diluted at a low density of OD600 ∼0.15 into fresh YDP media containing 1 mM SPM and the OD600 monitored over the indicated time, and F) assessment of *AGP2* expression by RT-PCR analysis. Total RNA (5 µg) from the indicated exponentially growing cells was used for the RT-PCR reaction. The *ACT1* gene was used as a control. Results are representative of three independent experiments.

On the basis of the aforementioned possible role of Agp2, we tested whether either *dur3*Δ or *sam3*Δ single mutants were resistant to the toxicity of high spermine concentrations using spot test analysis. As shown in Figure 1A, neither *dur3*Δ nor *sam3*Δ single mutants showed enhanced resistance to spermidine or spermine toxicity or to the spermidine derivative bleomycin-A5 (BLM-A5) when compared to the parental strain. The *agp2*Δ mutant was used as a positive control strain which displayed striking resistance to polyamines and BLM-A5 (Fig. 1A) [6]. None of the mutants showed any differential sensitivity to 4-nitroquinoline-1-oxide (4-NQO), a DNA-damaging agent without structural relationship with polyamines, which was used as a control for BLM-A5, indicating that BLM-A5 likely acts as a polyamine analogue. On the basis of these findings, we predicted that the *dur3*Δ and *sam3*Δ single mutants would show parental levels of polyamine uptake. As expected, the *agp2*Δ mutant was completely defective in [^14^C]-spermidine uptake, whereas the *dur3*Δ and *sam3*Δ single mutants showed parental levels of spermidine uptake (Fig. 1B). The latter observation is in disagreement with the previous results reported by Uemura *et al.*, i.e. that mutants disrupted for either the *DUR3* or *SAM3* genes displayed a lower velocity of spermidine uptake than the parental strain under similar uptake conditions with exponential cells [13] (see discussion).

Since Dur3 and Sam3 have been described as largely redundant high-affinity polyamine transporters, but could be deleted singly without affecting apparent spermidine uptake, we next examined the polyamine transport and toxicity phenotypes of *dur3*Δ *sam3*Δ double mutants. The *dur3*Δ *sam3*Δ cells obtained from a BY4741 parental background grew slightly slower in YPD media, unlike either *dur3*Δ or *sam3*Δ single mutants (data not shown). We observed that the *dur3*Δ *sam3*Δ double mutant was distinctly more resistant to polyamine toxicity than parental cells, but its tolerance was not nearly as pronounced as that found in *agp2*Δ cells upon growth on solid YPD medium containing spermine (Fig. 1C). The stronger tolerance to spermine toxicity observed in *agp2*Δ cells relative to the *dur3*Δ *sam3*Δ strain was independently confirmed by transferring exponentially growing cells to liquid YPD medium containing 1 mM spermine and monitoring the *OD*_600_ at various times. Indeed, under these conditions, the double mutant showed no growth defect and was less tolerant to spermine toxicity when compared to the *agp2*Δ mutants (Fig. 1D). This suggests that in addition to the high-affinity polyamine carriers Dur3 and Sam3, yeast cells also contain one or more low-affinity polyamine transporters. We next verified that the enhanced tolerance of *dur3*Δ *sam3*Δ cells towards spermine cytotoxicity was related to a defect in polyamine uptake. As shown in Figure 1E, the *dur3*Δ *sam3*Δ double mutant was completely defective in [^14^C]-spermidine uptake at a low substrate concentration (i.e. the high-affinity component, measured at 10 µM substrate), as compared to parental cells (see Discussion). Importantly, the defect in high-affinity spermidine uptake detected in the *dur3*Δ *sam3*Δ mutant was as pronounced as that found in the *agp2*Δ mutant (Fig. 1E). Taken together, these findings are consistent with the previous demonstration that Dur3 and Sam3 are high-affinity polyamine permeases [13] and reinforced the notion that Agp2 might act to regulate these two high-affinity transporters, as well as govern low-affinity polyamine transport. It is noteworthy that *AGP2* expression level was not detectably altered in the *dur3*Δ *sam3*Δ double mutant, as determined by RT-PCR (Fig. 1F), excluding the possibility that this double mutant influences *AGP2* expression.

### Agp2 localizes at the plasma membrane in the absence of polyamine transporting activity

**Figure 2:**
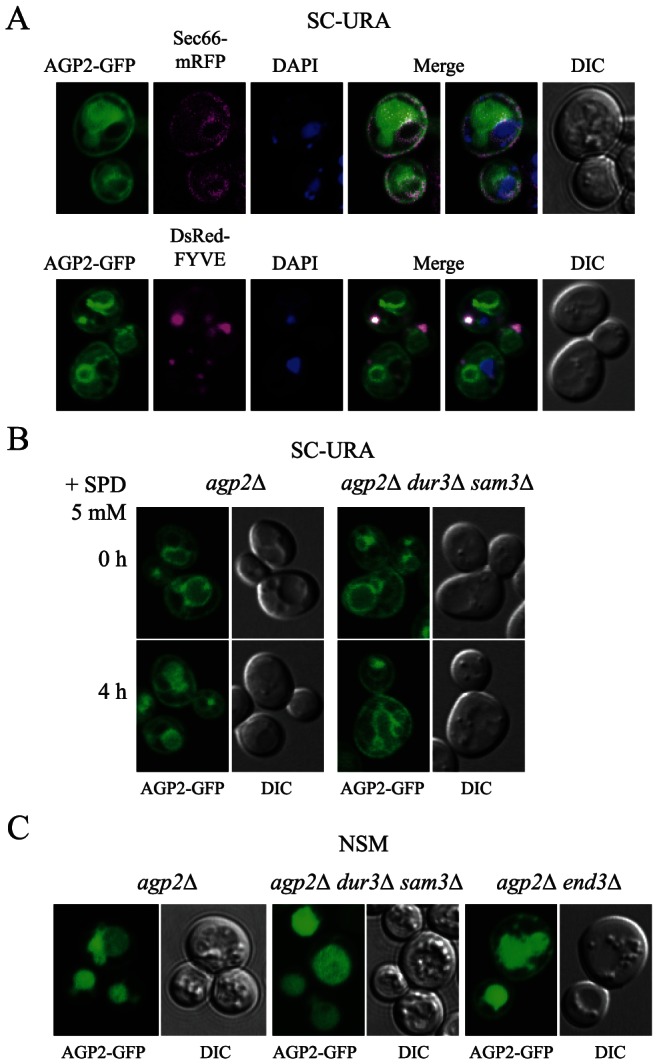
Agp2 is a ubiquitous protein localized at plasma membrane and internal compartments. **A**) Agp2-GFP localization in early exponential cells growing in selective medium and co-expressing ER marker Sec66-mRFP (above) or endosome marker DsRed-FYVE (below). DAPI staining is shown to localize nuclei in the same cells. **B**) Agp2-GFP is shown in *agp2Δ* or *agp2Δ dur3Δ sam3Δ* growing in the same conditions as in A), before, and 4 h after addition of spermidine 5 mM. **C**) Localization of Agp2-GFP in *agp2Δ*, *agp2Δ dur3Δ sam3Δ*, or *agp2Δ end3Δ* cells 24 h-starved for nitrogen.

The lack of high-affinity polyamine transport in *dur3*Δ *sam3*Δ prompted us to analyze Agp2 subcellular localization in this mutant. We investigated the localization of Agp2 by using a GFP C-terminally tagged version of the gene expressed under its own promoter, from the previously described plasmid pAGP2-GFP [6]. This plasmid expresses a functionally active fusion protein, which we have previously shown to rescue the polyamine uptake defect of the *agp2*Δ mutant. Cells growing exponentially in selective minimal medium showed a heterogeneous distribution of Agp2-GFP between plasma membrane, ER and endosomes (Fig. 2A). Under these conditions, Agp2-GFP partially colocalized with the mRFP-tagged ER translocation subcomplex subunit protein Sec66, which shows localization in cortical ER and nuclear envelope [23]. Partial colocalization of Agp2-GFP was also observed with the DsRed-tagged FYVE domain (a phosphatidylinositol 3-phosphate-binding domain found on endosomal membranes) [22] (Fig. 2A). The existence of intracellular pools of Agp2 located in different subcellular compartments is consistent with the known distribution of proteins possessing both transporter and sensor functions [31], [32]. This form of distribution may indicate that the activity of Agp2 is tightly regulated at the post-translational level by intracellular sorting, i.e., recycling or endocytosis, for rapid adaptation to environmental changes in the nutrient conditions.

Since *AGP2* is normally expressed at the transcriptional level in the *dur3*Δ *sam3*Δ mutant, the absence of polyamine transport in this mutant strongly suggests that Agp2 lacks the ability to directly transport polyamines as a plasma membrane transporter. Alternatively, the possibility remains that in the *dur3*Δ *sam3*Δ mutant Agp2 does not accumulate at the plasma membrane. In order to discern between these two possibilities, we monitored the localization of Agp2-GFP in the *agp2*Δ *dur3*Δ *sam3*Δ triple mutant (Fig. 2B). Our results showed that Agp2-GFP still accumulated at the plasma membrane of the *agp2*Δ *dur3*Δ *sam3*Δ cells growing exponentially in selective minimal medium. Under this condition, Agp2-GFP did not sensitize the *dur3*Δ *sam3*Δ double mutant to spermine or spermidine (data not shown), again excluding the possibility that Agp2 possesses the ability to directly transport polyamines. These findings, therefore, support the hypothesis that Agp2 might act as a plasma membrane polyamine sensor/regulator. Addition of polyamines (shown in Fig. 2B for the sublethal concentration of spermidine, 5 mM) did not cause apparent changes in the localization of Agp2-GFP, in either the *agp2*Δ or *agp2*Δ *dur3*Δ *sam3*Δ mutants, at least during an incubation period of up to 4 h (similar results were obtained with putrescine 5 mM, and spermine 0.05 mM; data not shown). However, Agp2-GFP plasma membrane localization was strongly dependent on the presence of an external nitrogen source. Lack of nitrogen caused vacuolar sorting of Agp2-GFP in both *agp2*Δ and *agp2*Δ *dur3*Δ *sam3*Δ mutants. Part of this vacuolar sorting is due to endocytosis of Agp2-GFP, since in the endocytosis deficient strain *agp2*Δ *end3*Δ, Agp2-GFP accumulated at the plasma membrane of nitrogen-starved cells (Fig. 2C).

### Expression of the genes DUR3, SAM3 and HNM1 is downregulated in the agp2Δ mutant

**Figure 3:**
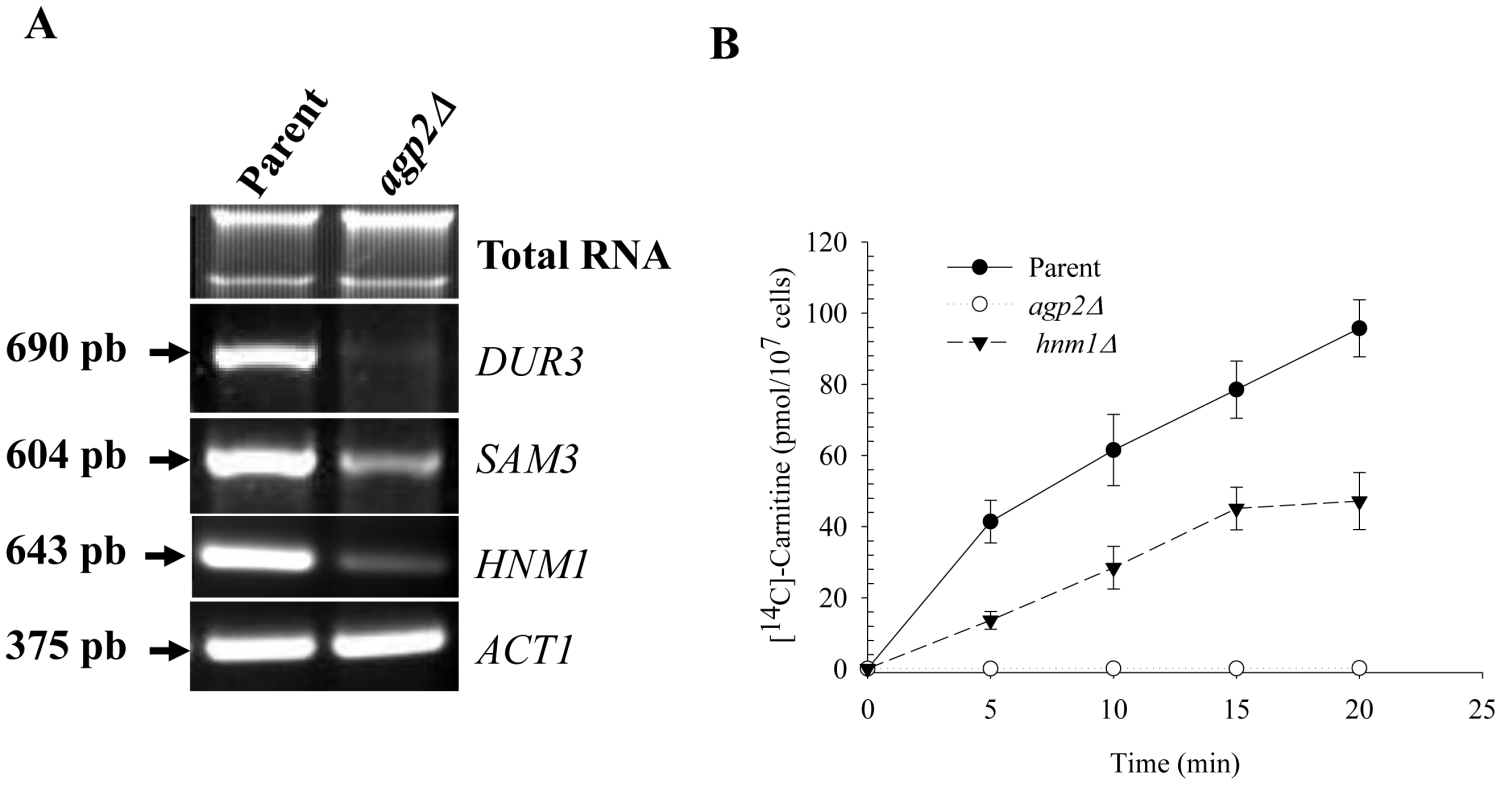
agp2Δ mutant downregulates DUR3, SAM3 and HNM1 expression and Hnm1 is required for L-carnitine uptake. **A**) RT-PCR analysis of the indicated transporter genes. Total RNA (2 µg) derived from the parent and *agp2Δ* mutant was analyzed for expression of the polyamine transporter genes *DUR3*, *SAM3*, as well as the choline transporter *HNM1* (see Materials and Methods). *ACT1* served as the control gene. The RT-PCR reaction was performed for 25 cycles and the expected size for each fragment is indicated by an arrow. **B**) *agp2Δ* and *hnm1Δ* single mutants are completely and partially defective, respectively, in L-carnitine uptake. Yeast strains were incubated with 10 µM of labelled [^14^C]-L-carnitine and samples withdrawn at the indicated time to monitor the uptake level. The data is the result of two independent analyses.

To address the hypothesis that Agp2 behaves as a regulator and controls the expression of the two high-affinity polyamine permease genes *DUR3* and *SAM3*, we performed RT-PCR analysis of total RNA isolated from the *agp2*Δ mutant and the parental strain. While the expression level of a control gene (*ACT1*) was identical in both parental and *agp2*Δ cells, *DUR3* and *SAM3* expression was considerably reduced in the *agp2*Δ mutant, with *DUR3* being the most profoundly affected (Fig. 3A). Since Agp2 was originally described as a L-carnitine transporter [3], we assessed whether its strongly positive action on L-carnitine uptake might in fact be explained by an indirect mechanism, *i.e.* by upregulation of a different transporter gene. We predicted that the choline permease Hnm1 might also act as an L-carnitine transporter on the basis of the similar quaternary amine structures of L-carnitine and choline, and as also suggested by an earlier report that L-carnitine is an effective competitive inhibitor of choline transport [33]. As shown in Figure 3B, the velocity of L-carnitine uptake was decreased by 40–50% in *hnm1*Δ mutant cells, as compared to the control strain, whereas *agp2*Δ cells were completely deficient in L-carnitine transport activity, confirming a previous report [3]. These data suggest for the first time that Hnm1 is a significant pathway for L-carnitine import, although another Agp2-dependent uptake mechanism must be invoked to fully account for L-carnitine transport activity (see Discussion). Consistent with a biologically significant role for Hnm1 in L-carnitine uptake, *HNM1* expression was barely detectable in the *agp2Δ* mutant (Fig. 3A). This confirms that a substantial fraction of L-carnitine is transported by Hnm1 into the cells and that the expression of this permease is tightly controlled by Agp2. Taken together, these findings strongly suggest that Agp2 acts on polyamine and L-carnitine transport via an indirect mechanism, *i.e.*, through regulation of expression of at least three permease genes, namely *DUR3* and *SAM3* in the case of high-affinity polyamine transport, and *HNM1* for L-carnitine transport. This regulation seems to share at least one similarity to the mode of action described for the amino acid Ssy1 and the glucose Snf3-Rgt2 sensors, namely the ability to regulate downstream targets at the transcriptional level [9], [34], [35].

### Microarray analysis reveals that Agp2 is a major regulator of gene expression

To investigate whether the postulated regulatory role of Agp2 extends to genes other than polyamine and L-carnitine permease genes, we performed three independent microarray analyses from total mRNA derived from the wild-type parent and *agp2Δ* mutant strains (see Materials and Methods). The inactivation of *AGP2* caused either the down- or upregulation of a much larger number of genes than anticipated (Table S1 and S2). Ontogeny analysis identified four major clusters of high statistical significance that were the most strongly depressed upon *AGP2* inactivation with a very strong bias (*P* = 7.5×10^−8^) towards transmembrane transport. Thus, in addition to *DUR3*, *SAM3* and *HNM1*, a large number of other transporter genes including at least four hexose permeases (*HXT1*, *HXT2*, *HXT3*, and *HXT13*) which function in maintaining a steep inwardly directed D-glucose gradient, as well as *TPO2*, which encodes one of the four plasma membrane drug/H^+^ exchangers of the major facilitator superfamily that excrete polyamines in yeast, were downregulated in *agp2*Δ mutants (Table 4 and Table S3). The three other clusters of downregulated genes represent those involved in (i) mating, cell adhesion and agglutination, several of which are under mating factor control and are essential components of the pheromone response pathway, (ii) vitamin and cofactor biosynthesis, and (iii) sulfur assimilation and methionine biosynthesis (Table 4 and Table S3).

**Table 4:** Members of the four major clusters of genes downregulated by *AGP2* deletion.

Transmembrane transport	Pheromone response pathway and mating	Vitamin and cofactor biosynthesis	Sulfur assimilation and methionine biosynthesis
*HNM1*	*STE2*	*PAN6*	*MET1*
*DUR3*	*FUS1*	*RIB1*	*UTR4*
*SAM3*	*FIG2*	*RIB2*	*MHT1*
*TOK1*	*FIG1*	*SPE4*	*CYS3*
*SUL1*	*MUC1*	*URA1*	*THI22*
*YMR279c*	*AGA2*		*BIO3*
*MUP3*	*AGA1*		*MMP1*
*NFT1*	*SAG1*		
*UGA4*	*PRM1*		
*ZRT1*	*PRM6*		
*TPO2*	*PRM5*		
*MCH5*	*ASG7*		
*YGL114w*	*PRM7*		
*YFL054c*	*FLO10*		
*ALR2*	*SPH1*		
*YOL163w*	*MFA1*		
*MMP1*	*MFA2*		
*YOL162w*	*GAS2*		
*STE6*	*MFA(ALPHA)1*		
*SUL2*			
*FCY22*			
*VHT1*			
*FAT1*			
*YOR378w*			
*THI7*			
*MCH4*			
*MMT1*			
*FUR4*			
*OPT1*			
*FRE1*			
*FLR1*			
*HXT1*			
*HXT2*			
*HXT3*			
*HXT13*			
*YFL040w*			
*MPH2*			
*RGT2*			
*MTH1*			
*MAL11*			
*MAL31*			

In a similar ontology analysis for the genes that were negatively regulated by *AGP2*, we identified three general functional classes consisting of genes involved in (i) lipid/fatty acid oxidation and mitochondrial oxidative metabolism (Krebs cycle and respiration), (ii) plasma and peroxisomal membrane transport and (iii) meiosis and sexual spore formation (Table 5 and Table S4). Thus, Agp2 seems to influence the expression of specific subgroups of genes.

**Table 5:** Members of the three major clusters of genes upregulated by *AGP2* deletion.

Oxidative metabolism	Transmembrane transport	Meiosis and spore formation
*POX1*	*PHO89*	*SPS100*
*CIT3*	*PHO84*	*AQY1*
*ADH2*	*SPL2*	*ADY2*
*NDE2*	*ATO3*	*FMP45*
*OLE1*	*MEP1*	*GIP1*
*ETR1*	*AQY1*	*SPR3*
*FAS2*	*ADY2*	*SMA1*
*ECI1*	*PMA2*	*OSW1*
*ZTA1*	*GIT1*	
*YIL057C*	*PXA1*	
*CTA1*	*OPT2*	
*ANT1*	*MGA1*	
*MBR1*	*YJR120W*	
***PDH1***		
***PAU18***		
***PAU6***		
***PAU20***		
***PAU4***		
***SPS19***		
*SAE3*		
*MAM1*		
*MER1*		
*MND1*		
*MEI5*		

**Figure 4:**
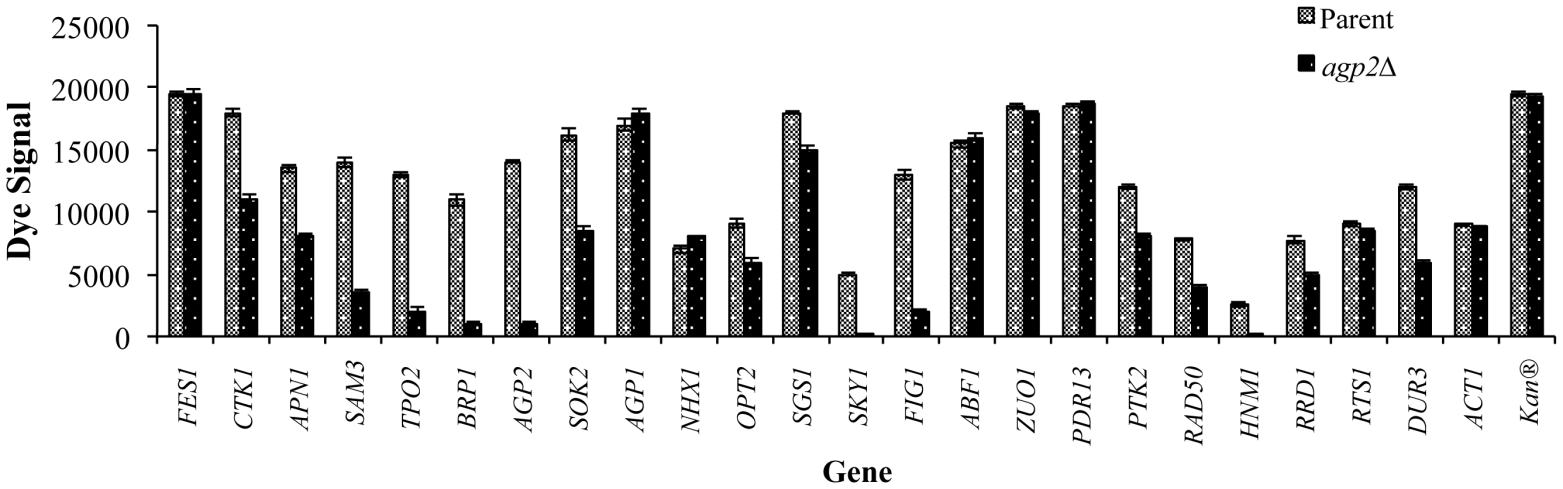
Simultaneous analysis of gene expression in the parent and agp2Δ mutant. GeXP multiplex expression analysis of 24 genes was performed from exponentially growing cultures under normal conditions. The data is derived from two independent experiments and expressed relative to *ACT1* and the internal PCR control *Kan^R^*.

To further confirm that Agp2 plays a major role in the regulation of gene expression, we measured mRNA levels for a set of genes identified from the microarray analysis using two independent approaches. In the first approach, we used a multiplex quantitative assay, namely the Gene eXpression Profiler (GeXP), to confirm the differences in the expression of 24 genes, which included both targets and non-targets, between *agp2*Δ mutants and the parental strain in a single reaction. This strategy utilizes RT-PCR and PCR followed by gene product analysis via capillary electrophoresis (see “Materials and Methods”) [27]. As shown in Figure 4, the key target genes *SKY1*, *SAM3*, *DUR3*, and *HNM1* were downregulated in the *agp2*Δ mutant as compared to the parent. In contrast, the non-target genes such as *ACT1*, *FES1*, *AGP1*, *PDR13* and *ZOU1* were not affected in the *agp2*Δ mutant (Fig. 4).

**Figure 5:**
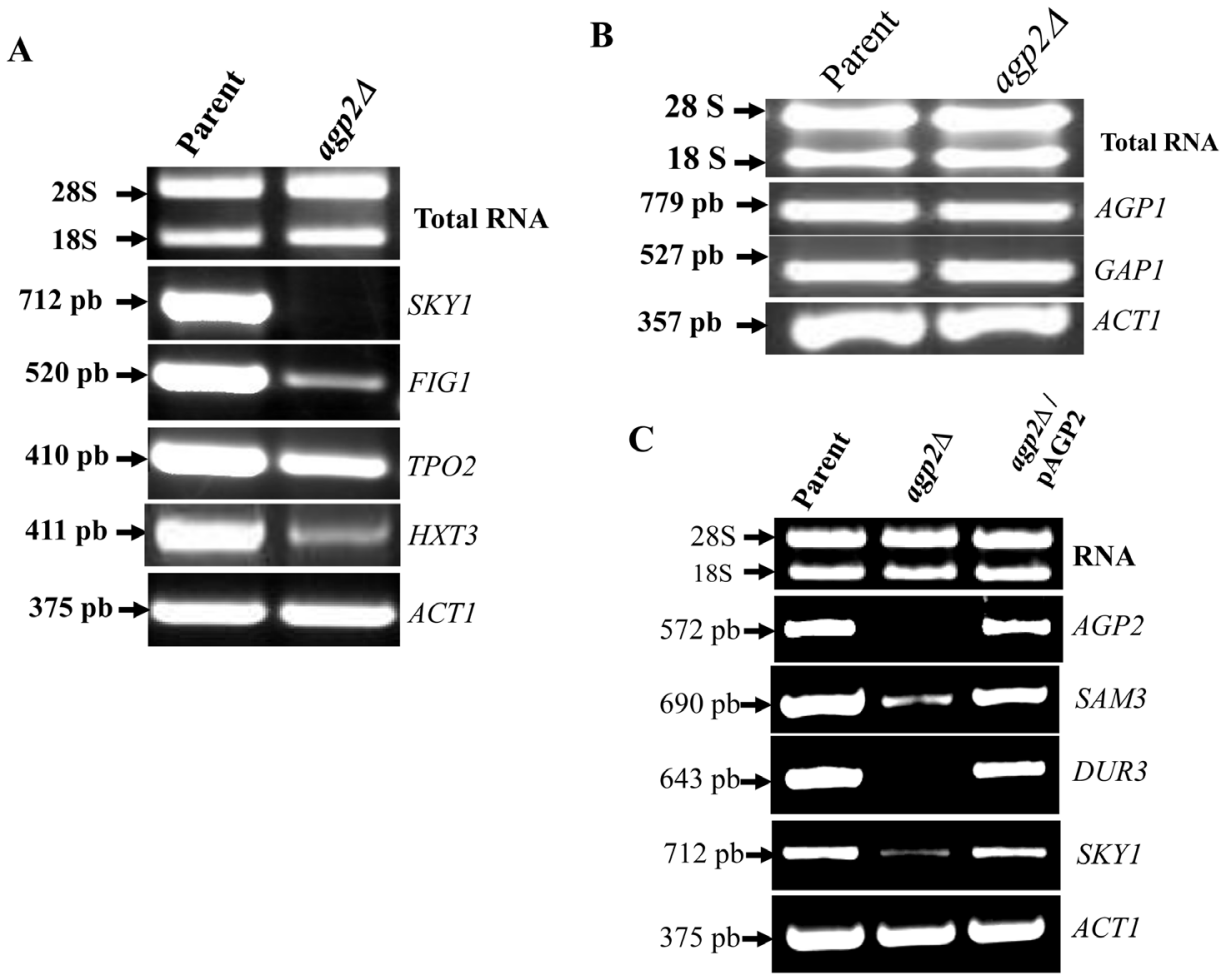
Validation of key Agp2 targeted and non-targeted genes by RT-PCR analysis. Total RNA (2 µg) derived from the parent and *agp2*Δ mutant was analyzed for gene expression as described for Fig. 3. **A**) expression analysis of Agp2-targeted genes. **B**) expression analysis of Agp2-non-targeted genes. **C**) The plasmid pAGP2 rescues expression of Agp2-targeted genes in the *agp2Δ* mutant. Total RNA (2 µg) derived from the parent, *agp2Δ* and the *agp2Δ* carrying the plasmid pAGP2-HIS was analyzed for gene expression as in panel A. *ACT1* served as the control gene. The size of the expected fragments are indicated by arrows.

In the second approach, we used RT-PCR and examined the expression pattern of a few individual genes. As shown in Figure 5A, the target genes *SKY1*, *FIG1*, *TPO2*, and *HXT3* were downregulated in the *agp2*Δ mutant, as compared to the control gene *ACT1*. In contrast, the non-target transporter genes *AGP1* and *GAP1* were not affected in *agp2*Δ mutants (Fig. 5B). We next verified whether ectopic expression of *AGP2* could restore the expression of target genes in the *agp2*Δ mutant. Introduction of the single copy plasmid pAGP2 restored nearly normal expression of the target genes *DUR3*, *SAM3* and *SKY1*, while having no effect on *ACT1* expression (Fig. 5C). Collectively, the above analyses confirm that Agp2 indeed governs the expression levels of a substantial subset of target genes in yeast.

### Sky1 is not involved in the transcriptional control of the polyamine permeases by Agp2

**Figure 6:**
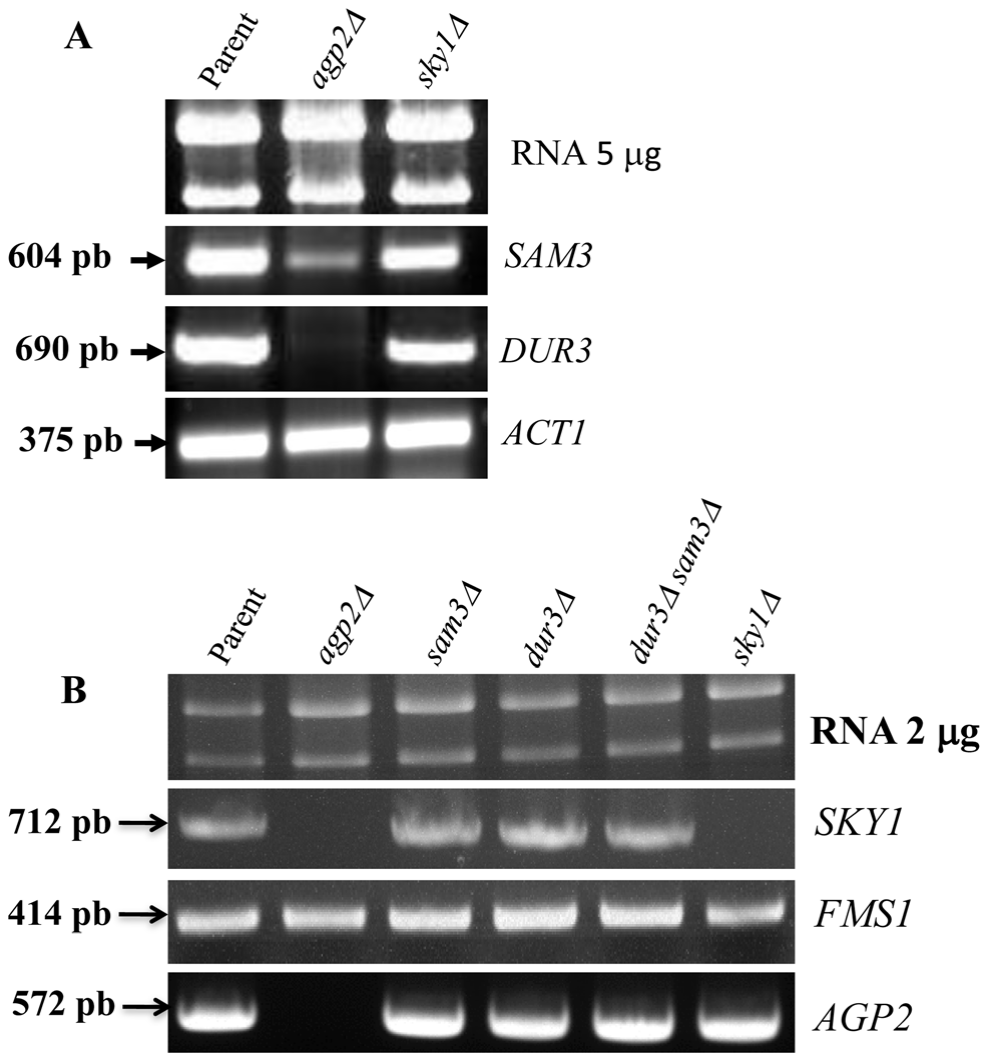
DUR3 and SAM3 expression are not affected in the sky1Δ mutant or vice versa. Total RNA (2 µg) derived from the indicated strains was analyzed for expression of *DUR3* and *SAM3* (**panel A**) and SKY1 (**panel B**). The RT-PCR conditions were the same as in Fig. 5, but the *FMS1* gene was used as a control.

From all the gene expression analyses data, *SKY1* was the most strongly downregulated gene (43-fold decrease from the microarray analysis), which led us to assess whether this SR kinase, shown to be a key factor responsible for both high- and low-affinity polyamine transport, could mediate Agp2 regulatory function [16]. As illustrated in Figure 6A, *SKY1* deletion did not alter the expression of the Agp2 target genes, *DUR3* and *SAM3*, thus eliminating Sky1 as an intermediate in the signaling mechanism leading to the positive action of Agp2 on the transcriptional expression of the two major polyamine permeases. It is noteworthy that the expression of *SKY1*, as in the case of *AGP2*, was not altered in the *dur3Δ sam3Δ* double mutant (Fig. 6B), excluding the possibility that these two permeases can influence this Agp2 target.

### Ste12, Sok2, Sfp1, Yap1 and Msn2 are predicted to transduce a major portion of the signal from Agp2

In order to obtain some insight on the potential pathway(s) responsible for the regulation of gene expression by Agp2 as a sensor, we performed an analysis of the frequency of (directly or indirectly) documented transcription factor recruitment for the expression of genes that were either downregulated by ≥2.5-fold (*n* = 140) or upregulated by ≥2.5-fold (*n* = 152), using the YEASTRACT algorithm (http://www.yeastract.com/formgroupbytf.php) [36]. More than 50% of the subset of genes found to be strongly downregulated in *agp2Δ* mutants were under the control of either Sfp1 or Ste12 (Table S5) at a frequency that was 57% and 51% higher than for the full list of ORFs tested, respectively. Ste12 was also the most highly represented transcription factor (48%) among the most strongly upregulated genes in *agp2*Δ cells (Table S6). In addition, a few other transcription factors (Yap1>Sok2>Msn2>Kar4>Dal80>Sut1>Msn4) and (Yap1>Sfp1>Aft1>Sok2>Msn2>Hap1>Rme1>Ime1>Ume6) were markedly overrepresented among the list of genes downregulated and upregulated, respectively, in the *agp2*Δ mutants. Importantly, the highest enrichment factor for potential transcription factor utilization for the regulation of genes that were either up- or downregulated in *agp2Δ* cells was found for Sok2 (2.44- and 2.46-fold, respectively) and Yap1 (2.54- and 2.12-fold, respectively). However, single mutation in any of the above transcription factor genes did not reveal the polyamine phenotypes displayed by the *agp2Δ* mutants (data not shown), raising the possibility that some of these factors could perform redundant functions in polyamine homeostasis. It is noteworthy that a subgroup of these dysregulated transcription factors, *e.g.*, Kar4, which specifically regulates meiosis and the karyogamy stages of the pheromone response pathway, controls target genes [37] that are among the most strongly affected by *AGP2* inactivation. Thus, these observations support a model that transcription factors are indeed involved in the signalling pathway by which Agp2 influences polyamine uptake.

## Discussion

We had previously documented that Agp2 affects high-affinity polyamine transport, although it was unclear whether this protein acts directly as a polyamine permease or as a regulator of polyamine transport, or both [6]. According to Uemura *et al.*, (2007) *S. cerevisiae* has only two high-affinity polyamine transporters, namely Dur3 and Sam3, while Agp2 plays virtually no role in polyamine uptake (see Fig. 6 in [13]), raising the question of whether *bona fide* Agp2 was actually examined in their study, as the text refers to Agp1 (*e.g.*, see p. 7 of Ref. 13, where the mutants are designated as *agp1Δ*). In a separate study, Porat *et al.*, (2005) claimed to re-isolate Agp2 from a high throughput screen designed to identify factors involved in protecting cells against polyamine toxicity and, in a similar manner as our previous report, demonstrated that Agp2 is indeed required for high affinity polyamine transport [6], [38]. As such, it is difficult to account for the major discrepancies between the results obtained by Uemura *et al.* and the data presented herein on Agp2 function. In this current study, we confirm and extend our previous demonstration that Agp2 plays an essential role in polyamine transport in yeast, and show that it acts as a novel regulator by sustaining the expression of several plasma membrane transporter genes, including *DUR3*, *SAM3* and *HNM1*. Hnm1 is shown for the first time to be involved in the uptake of L-carnitine into cells. In addition, Agp2 controls, either as an activator or a repressor, the expression of a relatively small subgroup of genes that are involved in well-defined biological processes such as the pheromone response pathway and vitamin and cofactor biosynthesis. Moreover, we clearly demonstrate the functional redundancy between Dur3 and Sam3 as polyamine permeases, since deletion of both genes is necessary to generate an *agp2*Δ-like phenotype, which is characterized by a strong defect in polyamine uptake as well as a marked resistance to toxic polyamines. We conclude that Agp2 is unlikely to be a high-affinity transporter with a dual role in polyamine and L-carnitine transport since (i) high concentrations of L-carnitine did not block polyamine uptake, as confirmed by the lack of protection afforded by L-carnitine against polyamine toxicity and (ii) the *dur3Δ sam3Δ* double mutant did not show a significant level of high-affinity polyamine uptake activity as would have been expected if Agp2 acts as a *bona fide* high-affinity transporter.

**Figure 7:**
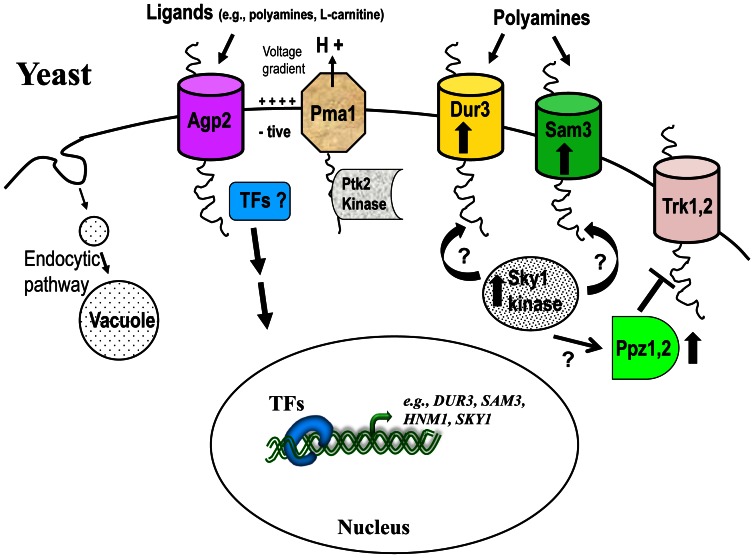
A model illustrating Agp2 function in regulating polyamine uptake. Agp2 could act to sense several nutrients such as polyamines and maintain the transcriptional expression of many genes including the regulatory kinase Sky1 and the two high affinity polyamine transporters Dur3 and Sam3. Similar to Agp2, Sky1 controls both high and low affinity polyamine transport. Since Sky1 does not affect *DUR3* or *SAM3* expression, we propose that it may activate these transporters by post-translational modification, *e.g.*, by phosphorylation. Furthermore, Sky1 has been shown to negatively regulate the Trk1 and 2 potassium transporters, perhaps via activation of the Ppz1 and 2 phosphatases, leading to polyamine uptake. *ppz1Δ ppz2Δ* mutants activate Trk1, 2 potassium uptake and contribute to polyamine resistance. The transcription factors (TFs) responsible for conveying Agp2 sensory function and culminating in gene activation remain to be identified.

The fact that Agp2 expression level and plasma membrane localization is normal in the *dur3*Δ *sam3*Δ double mutant and that this double mutant is much less tolerant to high spermine concentrations as compared to the *agp2Δ* mutant, raises the possibility that the additional resistance caused by *agp2Δ* mutant might be explained by the lack of a functional low-affinity polyamine permease. Consistent with the existence of a low-affinity transporter, Uemura *et al.* showed that the *dur3*Δ *sam3*Δ double mutant displayed an intermediate level of polyamine transport activity in the presence of a high (100 µM spermidine) substrate concentration. It is unlikely that Agp2 might directly act as a low-affinity permease, as overexpression of Agp2-GFP did not sensitize *sky1Δ* or the *dur3Δ sam3Δ* mutant to polyamines (data not shown). A more likely possibility is that Agp2 regulates the low-affinity polyamine uptake activity of an as yet unidentified permease. This is supported by several facts (i) Agp2 exerts stringent positive control on *SKY1* gene expression, encoding a SR kinase essential for both high- and low-affinity polyamine transport activities, (ii) deletion of either *AGP2* or *SKY1* leads to strong resistance against the toxicity of polyamine and polyamine analogues, (iii) *sky1Δ* mutants are strongly defective in polyamine transport, similar to that found in the *agp2*Δ mutant and the *dur3Δ sam3Δ* double mutant, and (iv) *AGP2* gene expression, as confirmed by Agp2-GFP, is normal in *sky1Δ* mutants precluding the possibility that Agp2 plays a direct role in low-affinity polyamine transport [5], [16], [17], [38], [39]. To date, the exact role of Sky1 in regulating both high- and low-affinity polyamine transports is not known. In the case of the high-affinity transport, Sky1 does not act downstream of Agp2 to positively regulate *DUR3* and *SAM3* gene transcription, but it could serve to activate Dur3 and Sam3 transport activities by phosphorylation [39]. In the case of the low-affinity transport, Sky1 is believed to activate the type 1 phosphatases Ppz1 and Ppz2, which inhibit the activity of the Trk1,2 K^+^ transporters, thus hyperpolarizing cells and increasing polyamine transport [16], [40], [41]. In the absence of Sky1, the increased activity of the Trk1,2 K^+^ permeases is believed to depolarize cells by opposing the proton motive potential built up by the Pma1 H^+^-pump leading to diminished uptake and consequently resistance to polyamine. Therefore, the positive action of Agp2 on Sky1 may ultimately increase low affinity polyamine uptake activity via the general increase in membrane potential that results from the inhibition of Trk1,2 K^+^ permeases as illustrated by the model shown in Figure 7. The latter explanation might also account for the fact that *agp2*Δ mutants are more strongly resistant to polyamine than *dur3*Δ *sam3*Δ double mutants since the former positively regulates both high-affinity, via Dur3 and Sam3, and low-affinity polyamine uptake via Sky1. Consistent with the model (Fig. 7), *ppz1Δ ppz2Δ* double mutants have been reported to be highly resistant to spermine in the presence of functional Trk1 and Trk2 [16], [40]. However, these studies did not directly examine if the *ppz1Δ ppz2Δ* double mutant is defective in polyamine uptake and whether the defect can be rescued by deletion of the *TRK1* and *TRK2* genes.

How Agp2 transmits a signal to modulate the expression of Dur3, Sam3, and Sky1 in order to maintain polyamine homeostasis is not known, although it could occur through one or more transcriptional activators in a manner similar to the Ssy1 transceptor [10]. In such a scenario, Agp2 would behave as a plasma membrane sensor or non transporting transceptor for polyamines. While our haploid genome-wide screen did not yield any such transcription factor [5], the microarray analysis performed herein has identified a number of transcription factor genes that could be involved in either the downregulation or upregulation of Agp2 target genes. According to the YEASTRACT algorithm [36], which predicts the frequency of putative transcription factors for the most strongly regulated genes identified in *agp2Δ* mutants, there is a strong bias in favour of several transcription factors including Sfp1, Ste12, Yap1, Sok2, Msn2, Aft1 and Gcn4 that could receive signals from Agp2 (Tables S5 and S6). However, the actual factor that transmits the signal from Agp2 remains elusive, perhaps due to functional redundancy.

The sum of the above observations on the pattern of gene regulation by Agp2 consistently supports the following tentative model that explains the physiological mechanism of action of Agp2 as a cell surface regulatory protein (see Fig. 7). An important unresolved issue of this model is the exact nature of the ligand(s) that stimulates Agp2 function in order to regulate the activation of the signalling pathways. The requirement for a functional *AGP2* gene for polyamine transport via its stringent positive regulation of *DUR3*, *SAM3* and *SKY1*, as well as for L-carnitine uptake at least through expression of *HNM1*, suggest a plausible connection of Agp2 with a metabolic shift towards gluconeogenesis and other carbohydrate storage. This metabolic shift would require an increased level of both coenzyme A and L-carnitine in order to transfer acetyl groups across the mitochondrial membrane [42]. Coenzyme A must be synthesized *de novo* from spermine, as spermine is the sole source of β-alanine that provides the initial precursor pantothenic acid for coenzyme A synthesis [43], [44], [45], [46]. For L-carnitine, the intracellular pool derived from the degradation of ε-trimethyllysine-containing proteins (*e.g.*, cytochrome *c*) that provides the initial substrate for L-carnitine biosynthesis might be insufficient, and thus must be attained through import from extracellular sources [47]. In this respect, the primary function of Agp2 might be to act as a sensor to expand the intracellular pool of both coenzyme A and L-carnitine. Whether polyamines and L-carnitine are indeed the actual ligand molecules sensed by Agp2, and the nature of the mechanism responsible for the activation of cellular signaling that ultimately leads to the down- or up-regulation of specific gene expression are still unanswered questions worth future investigation.

## Supporting Information

Figure S1 **The effect of L-carnitine on polyamine toxicity of parent cells.** Exponentially growing wild type cultures were coincubated without or with 10 mM L-carnitine and various concentrations of spermine for 1 h, followed by serial dilution and then plated onto solid yeast-peptone-dextrose (YPD) agar to score for survivors. The results are the averages of three independent experiments.
(TIF)

Table S1 **Genes downregulated in the absence of Agp2 function.**
(DOC)

Table S2 **Genes upregulated in the absence of Agp2 function.**
(DOC)

Table S3 **Functional category of downregulated genes in the absence of Agp2.**
(DOCX)

Table S4 **Functional category of upregulated genes in the absence of Agp2.**
(DOCX)

Table S5 **Predicted transcription factors that might signal the downregulation of genes in the absence of Agp2.**
(DOC)

Table S6 **Predicted transcription factors that might signal the upregulation of genes in the absence of Agp2.**
(DOC)
