# Correction: Agp2, a Member of the Yeast Amino Acid Permease Family, Positively Regulates Polyamine Transport at the Transcriptional Level

**DOI:** 10.1371/annotation/ff0ad3b6-fff4-4783-8c2e-968a95283ab8

**Published:** 2013-07-01

**Authors:** Mustapha Aouida, Marta Rubio Texeira, Johan M. Thevelein, Richard Poulin, Dindial Ramotar

The name of the second author was given incorrectly. The correct name is: Marta Rubio-Texeira. The correct citation is: Aouida M, Rubio-Texeira M, Thevelein JM, Poulin R, Ramotar D (2013) Agp2, a Member of the Yeast Amino Acid Permease Family, Positively Regulates Polyamine Transport at the Transcriptional Level. PLoS ONE 8(6): e65717. doi:10.1371/journal.pone.0065717. The correct abbreviation in Contributions is: MR. 

