# Supplementary material for: Agp2, a Member of the Yeast Amino Acid Permease Family, Positively Regulates Polyamine Transport at the Transcriptional Level
Source: PLoS One. 2013 Jun 3;8(6):e65717. doi: 10.1371/journal.pone.0065717 (PMC3670898; doi:10.1371/journal.pone.0065717)
Supplement: Table S1 — Genes downregulated in the absence of Agp2 function. (DOC) [file pone.0065717.s002.doc]

| **Supplemental Table S1: Genes downregulated in the absence of Agp2 function** | | | | | |
| --- | --- | --- | --- | --- | --- |
| **Probe no.** | **Gene ID** | **Annotation** | **Fold change** | **t value** | **P value** |
| 1769557_at | **SKY1** | SR protein kinase (SRPK) involved in regulating proteins involved in mRNA metabolism and cation homeostasis; similar to human SRPK1 | 0,021982 | -43,6956 | 0 |
| 1772126_at | **FIG1** | Integral membrane protein required for efficient mating; may participate in or regulate the low affinity Ca2+ influx system, which affects intracellular signaling and cell-cell fusion during mating | 0,088908 | -9,86096 | 4,001572E-09 |
| 1775220_at | **PRM1** | Pheromone-regulated multispanning membrane protein involved in membrane fusion during mating; predicted to have 5 transmembrane segments and a coiled coil domain; localizes to the shmoo tip; regulated by Ste12p | 0,093361 | -22,085 | 1,665335E-15 |
| 1777630_at | **URA1** | Dihydroorotate dehydrogenase, catalyzes the fourth enzymatic step in the de novo biosynthesis of pyrimidines, converting dihydroorotic acid into orotic acid | 0,10348 | -37,5605 | 0 |
| 1779450_at |  |  | 0,12232 | -9,70390 | 5,231866E-09 |
| 1777384_at | **PRM6** | Pheromone-regulated protein, predicted to have 2 transmembrane segments; regulated by Ste12p during mating | 0,12624 | -15,1810 | 1,928457E-12 |
| 1770957_at | **---** | Putative protein of unknown function; belongs to the DHA2 family of drug:H+ antiporters; YOR378W is not an essential gene | 0,14105 | -13,9044 | 9,661272E-12 |
| 1778591_at | **AGA1** | Anchorage subunit of a-agglutinin of a-cells, highly O-glycosylated protein with N-terminal secretion signal and C-terminal signal for addition of GPI anchor to cell wall, linked to adhesion subunit Aga2p via two disulfide bonds | 0,15426 | -12,5463 | 6,165668E-11 |
| 1774486_at | **SSA2** | ATP binding protein involved in protein folding and vacuolar import of proteins; member of heat shock protein 70 (HSP70) family; associated with the chaperonin-containing T-complex; present in the cytoplasm, vacuolar membrane and cell wall | 0,16325 | -11,0091 | 6,13576E-10 |
| 1780091_at |  |  | 0,19436 | -8,109313 | 9,445741E-08 |
| 1771190_at | **ATF2** | Alcohol acetyltransferase, may play a role in steroid detoxification; forms volatile esters during fermentation, which is important in brewing | 0,20061 | -8,566048 | 3,990191E-08 |
| 1778287_at | **---** | Dubious open reading frame unlikely to encode a protein, based on available experimental and comparative sequence data; partially overlaps the verified ORF RSM7/YJR113C | 0,21124 | -9,773911 | 4,640934E-09 |
| 1773377_at | **PRM5** | Pheromone-regulated protein, predicted to have 1 transmembrane segment; induced during cell integrity signaling | 0,22573 | -15,76894 | 9,539036E-13 |
| 1773613_at | **OPT1** | Proton-coupled oligopeptide transporter of the plasma membrane; also transports glutathione and phytochelatin; member of the OPT family | 0,23398 | -7,877919 | 1,477045E-07 |
| 1773225_at | **---** | Putative protein of unknown function; green fluorescent protein (GFP)-fusion protein localizes to the vacuole; YOR292C is not an essential gene | 0,23846 | -11,43814 | 3,15726E-10 |
| 1773889_at | **---** | Putative protein of unknown function | 0,24055 | -9,287347 | 1,080494E-08 |
| 1775529_at | **FCY22** | Putative purine-cytosine permease, very similar to Fcy2p but cannot substitute for its function | 0,24155 | -5,600502 | 1,757242E-05 |
| 1769832_at | **---** | Putative membrane protein of unknown function | 0,24770 | -6,08629 | 5,996619E-06 |
| 1773109_at | **DAL80** | Negative regulator of genes in multiple nitrogen degradation pathways; expression is regulated by nitrogen levels and by Gln3p; member of the GATA-binding family, forms homodimers and heterodimers with Deh1p | 0,26327 | -13,14949 | 2,65632E-11 |
| 1776218_at | **MFA1** | Mating pheromone a-factor, made by a cells; interacts with alpha cells to induce cell cycle arrest and other responses leading to mating; biogenesis involves C-terminal modification, N-terminal proteolysis, and export; also encoded by MFA2 | 0,26382 | -8,695872 | 3,138801E-08 |
| 1772292_at | **---** | Putative channel-like protein; similar to Fps1p; mediates passive diffusion of glycerol in the presence of ethanol | 0,26439 | -15,54026 | 1,250999E-12 |
| 1770144_at | **GAS2** | 1,3-beta-glucanosyltransferase, involved with Gas4p in spore wall assembly; has similarity to Gas1p | 0,26560 | -6,430065 | 2,85113E-06 |
| 1776721_at | **HXT1** | Low-affinity glucose transporter of the major facilitator superfamily, expression is induced by Hxk2p in the presence of glucose and repressed by Rgt1p when glucose is limiting | 0,26750 | -6,089409 | 5,95591E-06 |
| 1779682_at |  |  | 0,26976 | -12,04814 | 1,26665E-10 |
| 1778856_at |  |  | 0,27582 | -5,654063 | 1,558735E-05 |
| 1777945_at | **TPO2** | Polyamine transport protein specific for spermine; localizes to the plasma membrane; transcription of TPO2 is regulated by Haa1p; member of the major facilitator superfamily | 0,27597 | -10,30901 | 1,891902E-09 |
| 1770548_at | **---** | Putative protein of unknown function; identified as a heat-induced gene in a high-throughout screen; YMR279C is not an essential gene | 0,27813 | -7,993785 | 1,17974E-07 |
| 1776273_x_at | **IMD2** | Inosine monophosphate dehydrogenase, catalyzes the first step of GMP biosynthesis, expression is induced by mycophenolic acid resulting in resistance to the drug, expression is repressed by nutrient limitation | 0,28568 | -5,252786 | 3,854672E-05 |
| 1769716_at | **THI22** | Protein with similarity to hydroxymethylpyrimidine phosphate kinases; member of a gene family with THI20 and THI21; not required for thiamine biosynthesis | 0,28884 | -7,944614 | 1,29752E-07 |
| 1770682_at | **HXT3** | Low affinity glucose transporter of the major facilitator superfamily, expression is induced in low or high glucose conditions | 0,29674 | -7,767804 | 1,831795E-07 |
| 1773727_at | **---** | Putative cystathionine beta-lyase; involved in copper ion homeostasis and sulfur metabolism; null mutant displays increased levels of spontaneous Rad52 foci; expression induced by nitrogen limitation in a GLN3, GAT1-dependent manner | 0,29865 | -7,593526 | 2,583891E-07 |
| 1770394_at | **SSK22** | MAP kinase kinase kinase of the HOG1 mitogen-activated signaling pathway; functionally redundant with, and homologous to, Ssk2p; interacts with and is activated by Ssk1p; phosphorylates Pbs2p | 0,29941 | -12,1401 | 1,107131E-10 |
| 1773061_s_at | **HPF1** | Putative protein of unknown function; serine/threonine rich and highly similar to YOL155C, a putative glucan alpha-1,4-glucosidase; transcript is induced in both high and low pH environments; YIL169C is a non-essential gene /// Haze-protective mannoprotein that reduces the particle size of aggregated proteins in white wines | 0,29998 | -8,676297 | 3,254017E-08 |
| 1773649_at | **FUR4** | Uracil permease, localized to the plasma membrane; expression is tightly regulated by uracil levels and environmental cues | 0,30088 | -6,052605 | 6,454929E-06 |
| 1769943_at | **ACO2** | Putative mitochondrial aconitase isozyme; similarity to Aco1p, an aconitase required for the TCA cycle; expression induced during growth on glucose, by amino acid starvation via Gcn4p, and repressed on ethanol | 0,30105 | -13,42948 | 1,815748E-11 |
| 1774863_at | **---** | Putative protein of unknown function; YLR012C is not an essential gene | 0,30203 | -7,341616 | 4,27915E-07 |
| 1770834_at | **MIF2** | Kinetochore protein with homology to human CENP-C, required for structural integrity of the spindle during anaphase spindle elongation, interacts with histones H2A, H2B, and H4, phosphorylated by Ipl1p | 0,30435 | -10,10912 | 2,635183E-09 |
| 1778653_at |  |  | 0,30802 | -8,678292 | 3,242072E-08 |
| 1777383_at | **ECM3** | Non-essential protein of unknown function | 0,31076 | -10,45012 | 1,501325E-09 |
| 1772131_s_at | **---** | Putative protein of unknown function | 0,31202 | -5,837867 | 1,035589E-05 |
| 1779235_at |  |  | 0,31300 | -5,311879 | 3,370059E-05 |
| 1770859_at | **---** | Protein of unknown function that may interact with ribosomes, based on co-purification experiments; has similarity to alpha-D-glucosidase (maltase); authentic, non-tagged protein detected in purified mitochondria in high-throughput studies | 0,31307 | -5,23645 | 4,000769E-05 |
| 1776558_at | **HXT2** | High-affinity glucose transporter of the major facilitator superfamily, expression is induced by low levels of glucose and repressed by high levels of glucose | 0,31358 | -4,378155 | 0,000290665 |
| 1771649_at | **---** | Putative protein of unknown function; deletion mutant is viable | 0,32015 | -8,064541 | 1,029349E-07 |
| 1772534_s_at | **---** | Putative GPI protein /// Putative protein of unknown function; predicted to be a glycosylphosphatidylinositol-modified (GPI) protein | 0,32112 | -6,124303 | 5,519327E-06 |
| 1772209_at | **SHQ1** | Essential nuclear protein, required for accumulation of box H/ACA snoRNAs and for rRNA processing; interacts with Naf1p | 0,32189 | -7,556199 | 2,782934E-07 |
| 1779207_at |  |  | 0,32205 | -5,246818 | 3,907401E-05 |
| 1772284_at | **---** | Putative protein of unknown function; green fluorescent protein (GFP)-fusion protein localizes to the cytoplasm; YLR225C is not an essential gene | 0,32392 | -12,05353 | 1,256678E-10 |
| 1774403_at | **---** | Putative protein of unknown function; phosphorylated by Dbf2p-Mob1p in vitro; some strains contain microsatellite polymophisms at this locus; YLR177W is not an essential gene | 0,32674 | -8,144398 | 8,832161E-08 |
| 1778804_at |  |  | 0,33274 | -5,331296 | 3,224748E-05 |
| 1773829_at | **MPH2** | Alpha-glucoside permease, transports maltose, maltotriose, alpha-methylglucoside, and turanose; identical to Mph3p; encoded in a subtelomeric position in a region likely to have undergone duplication | 0,33469 | -6,159779 | 5,109044E-06 |
| 1773073_at | **MCH5** | Plasma membrane riboflavin transporter; facilitates the uptake of vitamin B2; required for FAD-dependent processes; sequence similarity to mammalian monocarboxylate permeases, however mutants are not deficient in monocarboxylate transport | 0,33515 | -9,623478 | 6,008476E-09 |
| 1771789_at | **ZPR1** | Essential protein with two zinc fingers, present in the nucleus of growing cells but relocates to the cytoplasm in starved cells via a process mediated by Cpr1p; binds to translation elongation factor eEF-1 (Tef1p) | 0,33580 | -6,092842 | 5,911435E-06 |
| 1778996_at |  |  | 0,33876 | -5,425518 | 2,605218E-05 |
| 1779158_at |  |  | 0,33963 | -5,631638 | 1,638897E-05 |
| 1772831_at | **ROG3** | Protein that binds to Rsp5p, which is a hect-type ubiquitin ligase, via its 2 PY motifs; has similarity to Rod1p; mutation suppresses the temperature sensitivity of an mck1 rim11 double mutant | 0,34589 | -10,65094 | 1,084422E-09 |
| 1771763_at | **---** | Putative protein of unknown function; green fluorescent protein (GFP)-fusion protein localizes to the cytoplasm and nucleus | 0,34716 | -5,877963 | 9,47705E-06 |
| 1772554_at | **GAL10** | UDP-glucose-4-epimerase, catalyzes the interconversion of UDP-galactose and UDP-D-glucose in galactose metabolism; also catalyzes the conversion of alpha-D-glucose or alpha-D-galactose to their beta-anomers | 0,34858 | -5,720412 | 1,344235E-05 |
| 1776992_at | **AGA2** | Adhesion subunit of a-agglutinin of a-cells, C-terminal sequence acts as a ligand for alpha-agglutinin (Sag1p) during agglutination, modified with O-linked oligomannosyl chains, linked to anchorage subunit Aga1p via two disulfide bonds | 0,34896 | -7,342388 | 4,272485E-07 |
| 1773888_at | **CYS3** | Cystathionine gamma-lyase, catalyzes one of the two reactions involved in the transsulfuration pathway that yields cysteine from homocysteine with the intermediary formation of cystathionine | 0,35218 | -4,551472 | 0,0001940609 |
| 1776766_at | **PRM7** | Pheromone-regulated protein, predicted to have one transmembrane segment; promoter contains Gcn4p binding elements | 0,35840 | -7,581597 | 2,645848E-07 |
| 1779506_at |  |  | 0,36033 | -5,474357 | 2,333309E-05 |
| 1773291_at | **---** | Putative protein of unknown function, has some homology to Ugp1p, which encodes UDP-glucose pyrophosphorylase | 0,36299 | -4,969886 | 7,365883E-05 |
| 1770279_at | **LYS1** | Saccharopine dehydrogenase (NAD+, L-lysine-forming), catalyzes the conversion of saccharopine to L-lysine, which is the final step in the lysine biosynthesis pathway | 0,36488 | -7,194371 | 5,769459E-07 |
| 1772199_at | **BIO3** | 7,8-diamino-pelargonic acid aminotransferase (DAPA), catalyzes the second step in the biotin biosynthesis pathway; BIO3 is in a cluster of 3 genes (BIO3, BIO4, and BIO5) that mediate biotin synthesis | 0,36999 | -9,817456 | 4,308795E-09 |
| 1776145_at | **---** | Putative protein of unknown function | 0,37066 | -5,005146 | 6,792224E-05 |
| 1773386_at | **---** | Putative protein of unknown function | 0,37222 | -3,502421 | 0,002242518 |
| 1773662_at | **---** | Putative protein of unknown function | 0,37352 | -5,098932 | 5,477277E-05 |
| 1773907_at | **---** | Protein of unknown function; localizes to membrane fraction; YCL049C is not an essential gene; | 0,37498 | -5,277705 | 3,642197E-05 |
| 1773168_at | **SUT1** | Transcription factor of the Zn[II]2Cys6 family involved in sterol uptake; involved in induction of hypoxic gene expression | 0,37708 | -9,210659 | 1,237665E-08 |
| 1771580_at | **---** | Protein kinase implicated in the Slt2p mitogen-activated (MAP) kinase signaling pathway; associates with Rlm1p | 0,37734 | -5,574411 | 1,863115E-05 |
| 1771019_at | **SIA1** | Protein of unassigned function involved in activation of the Pma1p plasma membrane H+-ATPase by glucose | 0,37909 | -6,027411 | 6,821067E-06 |
| 1770666_at | **BCD1** | Essential protein required for the accumulation of box C/D snoRNA | 0,38535 | -6,928319 | 9,973327E-07 |
| 1779738_at |  |  | 0,38623 | -5,473062 | 2,340133E-05 |
| 1771165_x_at | **HXT13** | Hexose transporter, induced in the presence of non-fermentable carbon sources, induced by low levels of glucose, repressed by high levels of glucose | 0,38728 | -7,040345 | 7,911284E-07 |
| 1772813_at | **HPF1** | Haze-protective mannoprotein that reduces the particle size of aggregated proteins in white wines | 0,38804 | -8,525591 | 4,301968E-08 |
| 1770303_at | **SUL1** | High affinity sulfate permease; sulfate uptake is mediated by specific sulfate transporters Sul1p and Sul2p, which control the concentration of endogenous activated sulfate intermediates | 0,38966 | -3,482376 | 0,002349013 |
| 1772275_at | **JJJ3** | Protein of unknown function, contains a J-domain, which is a region with homology to the E. coli DnaJ protein | 0,39037 | -5,720473 | 1,344055E-05 |
| 1777476_at | **---** | Putative protein of unknown function | 0,39040 | -4,967021 | 7,414608E-05 |
| 1777559_at | **PMU1** | Putative phosphomutase, contains a region homologous to the active site of phsophomutases; overexpression of the gene suppresses the histidine auxotrophy of an ade3 ade16 ade17 triple mutant and the temperature sensitivity of a tps2 mutant | 0,39694 | -7,240777 | 5,249249E-07 |
| 1772983_at | **---** | Putative protein of unknown function | 0,39770 | -4,481797 | 0,0002282498 |
| 1771752_at | **FUS1** | Membrane protein localized to the shmoo tip, required for cell fusion; expression regulated by mating pheromone; proposed to coordinate signaling, fusion, and polarization events required for fusion; potential Cdc28p substrate | 0,39851 | -7,101993 | 6,969508E-07 |
| 1779772_at |  |  | 0,39929 | -8,022807 | 1,115475E-07 |
| 1774390_at | **SNT309** | Component of NineTeen complex (NTC) containing Prp19p involved in mRNA splicing, interacts physically and genetically with Prp19p | 0,40272 | -3,847941 | 0,001003684 |
| 1771495_at | **HLR1** | Protein involved in regulation of cell wall composition and integrity and response to osmotic stress; overproduction suppresses a lysis sensitive PKC mutation; similar to Lre1p, which functions antagonistically to protein kinase A | 0,40369 | -7,391826 | 3,867178E-07 |
| 1778398_at | **---** | Putative protein of unknown function; the authentic, non-tagged protein is detected in highly purified mitochondria in high-throughput studies | 0,40427 | -6,086198 | 5,997824E-06 |
| 1780077_at |  |  | 0,40586 | -7,108747 | 6,873622E-07 |
| 1770098_at | **PGA3** | Essential protein required for maturation of Gas1p and Pho8p, protein trafficking; GFP-fusion protein localizes to the endoplasmic reticulum; null mutants have a cell separation defect | 0,40752 | -5,19487 | 4,398596E-05 |
| 1774845_at | **SMA2** | Protein of unknown function involved in the assembly of the prospore membrane during sporulation | 0,40755 | -3,294686 | 0,003620259 |
| 1772104_at | **YMR1** | Phosphatidylinositol 3-phosphate [PI(3)P] phosphatase, regulates the localization and levels of PI(3)P; involved in cytoplasm to vacuole (CVT) transport; has similarity to the conserved myotubularin dual specificity phosphatase family | 0,40960 | -8,895433 | 2,179563E-08 |
| 1773157_at | **STE2** | Receptor for alpha-factor pheromone; seven transmembrane-domain GPCR that interacts with both pheromone and a heterotrimeric G protein to initiate the signaling response that leads to mating between haploid a and alpha cells | 0,40997 | -7,13946 | 6,454416E-07 |
| 1776844_at | **---** | Putative protein of unknown function; deletion confers sensitivity to 4-(N-(S-glutathionylacetyl)amino) phenylarsenoxide (GSAO) | 0,41140 | -4,915471 | 8,349285E-05 |
| 1769388_at | **DUS3** | Dihydrouridine synthase, member of a widespread family of conserved proteins including Smm1p, Dus1p, and Dus4p; contains a consensus oleate response element (ORE) in its promoter region | 0,41180 | -8,13089 | 9,06336E-08 |
| 1778076_at | **---** | Putative protein of unknown function; YBR284W is not an essential gene; null mutant exhibits decreased resistance to rapamycin and wortmannin | 0,41513 | -5,698067 | 1,412881E-05 |
| 1772710_at | **THP2** | Subunit of the THO complex, which connects transcription elongation and mitotic recombination, and of the TREX complex, which is recruited to activated genes and couples transcription to mRNA export; involved in telomere maintenance | 0,41629 | -4,052807 | 0,0006217997 |
| 1769383_at | **MRS1** | Protein required for the splicing of two mitochondrial group I introns (BI3 in COB and AI5beta in COX1); forms a splicing complex, containing four subunits of Mrs1p and two subunits of the BI3-encoded maturase, that binds to the BI3 RNA | 0,41665 | -7,809485 | 1,688138E-07 |
| 1775820_at | **VHT1** | High-affinity plasma membrane H+-biotin (vitamin H) symporter; mutation results in fatty acid auxotrophy; 12 transmembrane domain containing major facilitator subfamily member; mRNA levels negatively regulated by iron deprivation and biotin | 0,41717 | -8,32197 | 6,302508E-08 |
| 1773565_at | **RIB4** | Lumazine synthase (6,7-dimethyl-8-ribityllumazine synthase, also known as DMRL synthase); catalyzes synthesis of immediate precursor to riboflavin | 0,41774 | -9,683126 | 5,421921E-09 |
| 1776709_at | **ERO1** | Thiol oxidase required for oxidative protein folding in the endoplasmic reticulum | 0,41874 | -6,579812 | 2,072164E-06 |
| 1776926_at | **---** | Putative protein of unknown function; deletion mutant is viable and has no detectable phenotype | 0,41937 | -6,713157 | 1,563453E-06 |
| 1770125_at | **---** | Putative protein of unknown function; YDL218W transcription is regulated by Azf1p and induced by starvation and aerobic conditions | 0,42142 | -4,065135 | 0,0006041281 |
| 1778769_at |  |  | 0,42447 | -5,337533 | 3,179447E-05 |
| 1770792_at | **THI7** | Plasma membrane transporter responsible for the uptake of thiamine, member of the major facilitator superfamily of transporters; mutation of human ortholog causes thiamine-responsive megaloblastic anemia | 0,42638 | -7,51452 | 3,024027E-07 |
| 1771363_at | **RTA1** | Protein involved in 7-aminocholesterol resistance; has seven potential membrane-spanning regions | 0,42916 | -3,669892 | 0,001520117 |
| 1770044_at | **TUB3** | Alpha-tubulin; associates with beta-tubulin (Tub2p) to form tubulin dimer, which polymerizes to form microtubules; expressed at lower level than Tub1p | 0,43191 | -6,254816 | 4,157271E-06 |
| 1775781_at | **MCH4** | Protein with similarity to mammalian monocarboxylate permeases, which are involved in transport of monocarboxylic acids across the plasma membrane; mutant is not deficient in monocarboxylate transport | 0,43246 | -8,582202 | 3,872337E-08 |
| 1771286_at | **---** | Putative dihydrokaempferol 4-reductase | 0,43563 | -6,941208 | 9,710209E-07 |
| 1773541_at | **RGT2** | Plasma membrane glucose receptor, highly similar to Snf3p; both Rgt2p and Snf3p serve as transmembrane glucose sensors generating an intracellular signal that induces expression of glucose transporter (HXT) genes | 0,43652 | -6,067741 | 6,244699E-06 |
| 1779866_at |  |  | 0,43736 | -6,267827 | 4,041939E-06 |
| 1771051_at | **LYS4** | Homoaconitase, catalyzes the conversion of homocitrate to homoisocitrate, which is a step in the lysine biosynthesis pathway | 0,44057 | -7,338079 | 4,309827E-07 |
| 1770179_at | **PRM10** | Pheromone-regulated protein, predicted to have 5 transmembrane segments | 0,44063 | -4,064939 | 0,0006044055 |
| 1772536_at | **ECM34** | Putative protein of unknown function, member of the DUP380 subfamily of conserved, often subtelomerically-encoded proteins | 0,44251 | -3,711605 | 0,001379425 |
| 1770231_at | **KTI12** | Protein that plays a role, with Elongator complex, in modification of wobble nucleosides in tRNA; involved in sensitivity to G1 arrest induced by zymocin; interacts with chromatin throughout the genome; also interacts with Cdc19p | 0,44371 | -6,173041 | 4,963824E-06 |
| 1774361_at | **COQ2** | Para hydroxybenzoate: polyprenyl transferase, catalyzes the second step in ubiquinone (coenzyme Q) biosynthesis | 0,44408 | -7,705209 | 2,071723E-07 |
| 1776896_at | **SPE4** | Spermine synthase, required for the biosynthesis of spermine and also involved in biosynthesis of pantothenic acid | 0,44430 | -9,617573 | 6,070026E-09 |
| 1780155_at |  |  | 0,44544 | -6,340815 | 3,453175E-06 |
| 1773147_at | **MEF1** | Mitochondrial elongation factor involved in translational elongation | 0,44640 | -4,822838 | 0,0001033992 |
| 1779485_at |  |  | 0,44650 | -4,996193 | 6,933435E-05 |
| 1773044_at | **SPT8** | Subunit of the SAGA transcriptional regulatory complex but not present in SAGA-like complex SLIK/SALSA, required for SAGA-mediated inhibition at some promoters | 0,44721 | -8,011527 | 1,14001E-07 |
| 1771914_at | **STB6** | Protein that binds Sin3p in a two-hybrid assay | 0,44768 | -2,842645 | 0,01006003 |
| 1780016_at |  |  | 0,44892 | -6,874444 | 1,115516E-06 |
| 1775057_at | **MF(ALPHA)1** | Mating pheromone alpha-factor, made by alpha cells; interacts with mating type a cells to induce cell cycle arrest and other responses leading to mating; also encoded by MF(ALPHA)2, although MF(ALPHA)1 produces most alpha-factor | 0,45233 | -4,768254 | 0,0001173168 |
| 1776554_at | **MSH5** | Protein of the MutS family, forms a dimer with Msh4p that facilitates crossovers between homologs during meiosis; msh5-Y823H mutation confers tolerance to DNA alkylating agents; homologs present in C. elegans and humans | 0,45336 | -8,059596 | 1,039184E-07 |
| 1778355_at | **KAR4** | Transcription factor required for gene regulation in response to pheromones; also required during ss; exists in two forms, a slower-migrating form more abundant during vegetative growth and a faster-migrating form induced by pheromone | 0,45475 | -6,526435 | 2,321025E-06 |
| 1773034_at | **ARE1** | Acyl-CoA:sterol acyltransferase, isozyme of Are2p; endoplasmic reticulum enzyme that contributes the major sterol esterification activity in the absence of oxygen | 0,45633 | -6,052649 | 6,454309E-06 |
| 1773258_at | **MAK21** | Constituent of 66S pre-ribosomal particles, required for large (60S) ribosomal subunit biogenesis; involved in nuclear export of pre-ribosomes; required for maintenance of dsRNA virus; homolog of human CAATT-binding protein | 0,45642 | -7,079673 | 7,296347E-07 |
| 1776869_at | **---** | Putative protein of unknown function; green fluorescent protein (GFP)-fusion protein co-localizes with clathrin-coated vesicles | 0,45850 | -7,803321 | 1,708626E-07 |
| 1777293_at | **KTI11** | Protein required for synthesis of diphthamide, a modified histidine residue of translation elongation factor 2; functions with Dph1p, Dph2p, Jjj3p, and Dph5p; required, with Elongator complex, for modification of wobble nucleosides in tRNA | 0,45871 | -5,336988 | 3,183372E-05 |
| 1779508_at |  |  | 0,45872 | -8,697089 | 3,131778E-08 |
| 1770115_at | **SPH1** | Protein involved in shmoo formation and bipolar bud site selection; homologous to Spa2p, localizes to sites of polarized growth in a cell cycle dependent- and Spa2p-dependent manner, interacts with MAPKKs Mkk1p, Mkk2p, and Ste7p | 0,45879 | -6,147506 | 5,247322E-06 |
| 1776354_s_at | **ERR1 /// ERR2 /// ERR3** | Protein of unknown function, has similarity to enolases | 0,46197 | -6,280101 | 3,936151E-06 |
| 1778823_at |  |  | 0,46204 | -4,612492 | 0,0001683836 |
| 1771287_at | **ERG4** | C-24(28) sterol reductase, catalyzes the final step in ergosterol biosynthesis; mutants are viable, but lack ergosterol | 0,46269 | -5,846119 | 1,016844E-05 |
| 1775699_at | **HOG1** | Mitogen-activated protein kinase involved in osmoregulation via three independent osmosensors; mediates the recruitment and activation of RNA Pol II at Hot1p-dependent promoters; localization regulated by Ptp2p and Ptp3p | 0,46271 | -6,672756 | 1,702329E-06 |
| 1775912_at | **COX5B** | Subunit Vb of cytochrome c oxidase, which is the terminal member of the mitochondrial inner membrane electron transport chain; predominantly expressed during anaerobic growth while its isoform Va (Cox5Ap) is expressed during aerobic growth | 0,46705 | -4,663218 | 0,0001496663 |
| 1775226_at | **---** | Putative protein of unknown function; YBR063C is not an essential gene | 0,46788 | -8,975243 | 1,88641E-08 |
| 1774092_at | **RRN11** | Protein required for rDNA transcription by RNA polymerase I, component of the core factor (CF) of rDNA transcription factor, which also contains Rrn6p and Rrn7p | 0,46994 | -6,633585 | 1,849127E-06 |
| 1771042_at | **UNG1** | Uracil-DNA glycosylase, required for repair of uracil in DNA formed by spontaneous cytosine deamination, not required for strand-specific mismatch repair, cell-cycle regulated, expressed in late G1, localizes to mitochondria and nucleus | 0,47271 | -6,905289 | 1,046188E-06 |
| 1772865_at | **YGK3** | Protein kinase related to mammalian glycogen synthase kinases of the GSK-3 family; GSK-3 homologs (Mck1p, Rim11p, Mrk1p, Ygk3p) are involved in control of Msn2p-dependent transcription of stress responsive genes and in protein degradation | 0,47283 | -7,188934 | 5,833805E-07 |
| 1774906_at | **RGS2** | Negative regulator of glucose-induced cAMP signaling; directly activates the GTPase activity of the heterotrimeric G protein alpha subunit Gpa2p | 0,47346 | -5,642514 | 1,599506E-05 |
| 1777531_at | **---** | Uncharacterized protein of unknown function | 0,47388 | -6,322069 | 3,595426E-06 |
| 1771392_at | **FRE1** | Ferric reductase and cupric reductase, reduces siderophore-bound iron and oxidized copper prior to uptake by transporters; expression induced by low copper and iron levels | 0,47413 | -5,301542 | 3,450115E-05 |
| 1779741_at |  |  | 0,47681 | -5,497696 | 2,213778E-05 |
| 1772597_at | **---** | Putative protein of unknown function; localizes to the membrane fraction; YCR101C is not an essential gene | 0,47709 | -2,552416 | 0,01897781 |
| 1776151_at | **MTO1** | Mitochondrial protein, forms a heterodimer complex with Mss1p that performs the 5-carboxymethylaminomethyl modification of the wobble uridine base in mitochondrial tRNAs; required for respiration in paromomycin-resistant 15S rRNA mutants | 0,47849 | -6,579536 | 2,073376E-06 |
| 1769523_at | **---** | Protein proposed to be associated with the nuclear pore complex | 0,47875 | -6,560393 | 2,159358E-06 |
| 1777443_at | **SOH1** | Subunit of the RNA polymerase II mediator complex; associates with core polymerase subunits to form the RNA polymerase II holoenzyme; involved in telomere maintenance; conserved with other metazoan MED31 subunits | 0,47957 | -9,481121 | 7,690909E-09 |
| 1777495_at | **AST1** | Peripheral membrane protein that interacts with the plasma membrane ATPase Pma1p and has a role in its targeting to the plasma membrane, possibly by influencing its incorporation into lipid rafts | 0,48013 | -2,82039 | 0,01056912 |
| 1775408_at | **---** | Putative protein of unknown function; the authentic, non-tagged protein is detected in highly purified mitochondria in high-throughput studies | 0,48197 | -5,26429 | 3,755057E-05 |
| 1774239_at | **---** | Putative protein of unknown function; YGR079W is not an essential gene | 0,48308 | -3,749896 | 0,001261669 |
| 1770071_at | **THI4** | Thiazole synthase, catalyzes formation of the thiazole moiety of thiamin pyrophosphate; required for thiamine biosynthesis and for mitochondrial genome stability | 0,48356 | -5,78886 | 1,154441E-05 |
| 1775215_at | **---** | Protein of unknown function that may interact with ribosomes; periodically expressed during the yeast metabolic cycle; phosphorylated in vitro by the mitotic exit network (MEN) kinase complex, Dbf2p/Mob1p | 0,48424 | -6,043067 | 6,591118E-06 |
| 1779018_at |  |  | 0,48468 | -7,906228 | 1,397894E-07 |
| 1774952_at | **SUL2** | High affinity sulfate permease; sulfate uptake is mediated by specific sulfate transporters Sul1p and Sul2p, which control the concentration of endogenous activated sulfate intermediates | 0,48503 | -7,437582 | 3,527434E-07 |
| 1769837_at | **TOS3** | Protein kinase, related to and functionally redundant with Elm1p and Sak1p for the phosphorylation and activation of Snf1p; functionally orthologous to LKB1, a mammalian kinase associated with Peutz-Jeghers cancer-susceptibility syndrome | 0,48510 | -4,088215 | 0,000572384 |
| 1770011_at | **RIB2** | DRAP deaminase, catalyzes the third step of the riboflavin biosynthesis pathway; cytoplasmic tRNA pseudouridine synthase involved in pseudouridylation of cytoplasmic tRNAs at position 32 | 0,48694 | -9,614558 | 6,101699E-09 |
| 1771388_at | **---** | Putative protein of unknown function; epitope tagged protein localizes to the cytoplasm | 0,48818 | -6,065831 | 6,270845E-06 |
| 1778989_at |  |  | 0,49025 | -5,168206 | 4,67467E-05 |
| 1778791_at |  |  | 0,49134 | -6,403369 | 3,018956E-06 |
| 1774944_s_at | **MPH2 /// MPH3** | Alpha-glucoside permease, transports maltose, maltotriose, alpha-methylglucoside, and turanose; identical to Mph3p; encoded in a subtelomeric position in a region likely to have undergone duplication /// Alpha-glucoside permease, transports maltose, maltotriose, alpha-methylglucoside, and turanose; identical to Mph2p; encoded in a subtelomeric position in a region likely to have undergone duplication | 0,49161 | -3,227262 | 0,004224778 |
| 1778452_at | **MRP4** | Mitochondrial ribosomal protein of the small subunit | 0,49407 | -4,744058 | 0,000124079 |
| 1775446_at | **MRS2** | Mitochondrial inner membrane Mg(2+) channel, required for maintenance of intramitochondrial Mg(2+) concentrations at the correct level to support splicing of group II introns | 0,49451 | -4,114825 | 0,00053785 |
| 1772730_at | **CRR1** | Putative glycoside hydrolase of the spore wall envelope; required for normal spore wall assembly, possibly for cross-linking between the glucan and chitosan layers; expressed during sporulation | 0,49552 | -3,526415 | 0,002121276 |
| 1773804_at | **RAD16** | Protein that recognizes and binds damaged DNA in an ATP-dependent manner (with Rad7p) during nucleotide excision repair; subunit of Nucleotide Excision Repair Factor 4 (NEF4) and the Elongin-Cullin-Socs (ECS) ligase complex | 0,49604 | -5,216346 | 4,188326E-05 |
| 1774833_at | **COX17** | Copper metallochaperone that transfers copper to Sco1p and Cox11p for eventual delivery to cytochrome c oxidase | 0,49658 | -5,552585 | 1,956682E-05 |
| 1770945_at | **NMD4** | Protein interacting with Nam7p, may be involved in the nonsense-mediated mRNA decay pathway | 0,49892 | -2,922646 | 0,00841715 |
| 1772716_at | **---** | Putative protein of unknown function with similarity to TFC7 and phosphotranfer enzymes in prokaryotes; a deletion mutant shows alterations in glucose metabolism; green fluorescent protein (GFP)-fusion protein localizes to the cytoplasm and nucleus | 0,49933 | -4,717392 | 0,0001319891 |
| 1771634_at | **TAH11** | DNA replication licensing factor, required for pre-replication complex assembly | 0,49964 | -5,92969 | 8,454895E-06 |
