# Supplementary material for: Agp2, a Member of the Yeast Amino Acid Permease Family, Positively Regulates Polyamine Transport at the Transcriptional Level
Source: PLoS One. 2013 Jun 3;8(6):e65717. doi: 10.1371/journal.pone.0065717 (PMC3670898; doi:10.1371/journal.pone.0065717)
Supplement: Table S2 — Genes upregulated in the absence of Agp2 function. (DOC) [file pone.0065717.s003.doc]

| **Supplemental Table S2: Genes upregulated in the absence of Agp2 function** | | | | | |
| --- | --- | --- | --- | --- | --- |
| **Probe number** | **Gene ID** | **Annotation** | **Fold change** | **t value** | **P-value** |
| RPTR-Sc-AJ002682-1_s_at | **---** | --- | 858,36 | 66,48991 | 0 |
| AFFX-r2-Sc-25SrRNA-3_at | **---** | --- | 197,65 | 18,18437 | 6,594725E-14 |
| AFFX-25srRnae_at | **---** | --- | 190,50 | 18,77042 | 3,608225E-14 |
| 1771373_at | **SPS100** | Protein required for spore wall maturation; expressed during sporulation; may be a component of the spore wall | 14,845 | 5,814374 | 1,090916E-05 |
| AFFX-r2-Sc-25SrRNA-M_at | **---** | --- | 12,002 | 5,825172 | 1,06512E-05 |
| 1775402_at | **SPL2** | Protein with similarity to cyclin-dependent kinase inhibitors, overproduction suppresses a plc1 null mutation; green fluorescent protein (GFP)-fusion protein localizes to the cytoplasm in a punctate pattern | 8,5740 | 11,96743 | 1,42642E-10 |
| 1769766_at | **PHO89** | Na+/Pi cotransporter, active in early growth phase; similar to phosphate transporters of Neurospora crassa; transcription regulated by inorganic phosphate concentrations and Pho4p | 7,7683 | 10,33068 | 1,825639E-09 |
| 1778111_at | **ATO3** | Plasma membrane protein, regulation pattern suggests a possible role in export of ammonia from the cell; member of the TC 9.B.33 YaaH family of putative transporters | 7,0308 | 15,75987 | 9,641177E-13 |
| AFFX-25srRnad_at | **---** | --- | 6,7353 | 8,737009 | 2,910268E-08 |
| AFFX-25srRnab_at | **---** | --- | 6,5754 | 5,797348 | 1,132899E-05 |
| 1772556_at | **PIR1** | O-glycosylated protein required for cell wall stability; attached to the cell wall via beta-1,3-glucan; mediates mitochondrial translocation of Apn1p; expression regulated by the cell integrity pathway and by Swi5p during the cell cycle | 6,2301 | 9,442849 | 8,222088E-09 |
| 1776791_at | **POX1** | Fatty-acyl coenzyme A oxidase, involved in the fatty acid beta-oxidation pathway; localized to the peroxisomal matrix | 5,8167 | 9,189643 | 1,284758E-08 |
| 1771695_at | **CIT3** | Citrate synthase, catalyzes the condensation of acetyl coenzyme A and oxaloacetate to form citrate, mitochondrial isozyme involved in the TCA cycle | 5,5736 | 12,27616 | 9,085144E-11 |
| 1774070_at | **ADH2** | Glucose-repressible alcohol dehydrogenase II, catalyzes the conversion of ethanol to acetaldehyde; involved in the production of certain carboxylate esters; regulated by ADR1 | 5,3415 | 15,54453 | 1,24456E-12 |
| 1772681_s_at | **---** | Hypothetical protein | 5,1495 | 8,929632 | 2,048544E-08 |
| RPTR-Sc-J01347-1_at | **---** | --- | 5,1450 | 6,694062 | 1,627576E-06 |
| 1776107_at | **---** | Putative protein of unknown function; identified by gene-trapping, microarray-based expression analysis, and genome-wide homology searching | 5,1424 | 6,151668 | 5,200003E-06 |
| 1770749_at | **DSE1** | Daughter cell-specific protein, may participate in pathways regulating cell wall metabolism; deletion affects cell separation after division and sensitivity to drugs targeted against the cell wall | 5,0642 | 11,4351 | 3,171947E-10 |
| 1769959_at | **NDE2** | Mitochondrial external NADH dehydrogenase, catalyzes the oxidation of cytosolic NADH; Nde1p and Nde2p are involved in providing the cytosolic NADH to the mitochondrial respiratory chain | 5,0214 | 7,63006 | 2,403307E-07 |
| 1772751_at | **---** | Putative protein of unknown function; identified by fungal homology and RT-PCR | 4,7921 | 9,396284 | 8,920203E-09 |
| AFFX-18srRnab_at | **---** | --- | 4,7898 | 4,348994 | 0,0003111423 |
| 1780248_at | **AQY1** | Spore-specific water channel that mediates the transport of water across cell membranes, developmentally controlled; may play a role in spore maturation, probably by allowing water outflow, may be involved in freeze tolerance | 4,7584 | 6,257007 | 4,137619E-06 |
| 1770692_at | **PHO84** | High-affinity inorganic phosphate (Pi) transporter and low-affinity manganese transporter; regulated by Pho4p and Spt7p; mutation confers resistance to arsenate; exit from the ER during maturation requires Pho86p | 4,7238 | 12,70427 | 4,930778E-11 |
| 1770580_at | **ADY2** | Acetate transporter required for normal sporulation | 4,6563 | 15,38582 | 1,50524E-12 |
| 1777707_at | **---** | Putative protein of unknown function; the authentic, non-tagged protein is detected in highly purified mitochondria in high-throughput studies | 4,5420 | 12,23381 | 9,660017E-11 |
| 1774888_at | **---** | Minor succinate dehydrogenase isozyme; homologous to Sdh1p, the major isozyme reponsible for the oxidation of succinate and transfer of electrons to ubiquinone; induced during the diauxic shift in a Cat8p-dependent manner | 4,5391 | 11,63986 | 2,324896E-10 |
| AFFX-25srRnac_at | **---** | --- | 4,5306 | 4,508977 | 0,0002142439 |
| 1779129_at | **INO1** | Inositol 1-phosphate synthase, involved in synthesis of inositol phosphates and inositol-containing phospholipids; transcription is coregulated with other phospholipid biosynthetic genes by Ino2p and Ino4p, which bind the UASINO DNA element | 4,2416 | 9,068038 | 1,596363E-08 |
| 1779119_at | **---** | Putative protein of unknown function | 4,1163 | 11,37112 | 3,498271E-10 |
| 1774263_at | **EGT2** | Glycosylphosphatidylinositol (GPI)-anchored cell wall endoglucanase required for proper cell separation after cytokinesis, expression is activated by Swi5p and tightly regulated in a cell cycle-dependent manner | 4,0888 | 11,53129 | 2,739791E-10 |
| 1769986_at | **HBT1** | Substrate of the Hub1p ubiquitin-like protein that localizes to the shmoo tip (mating projection); mutants are defective for mating projection formation, thereby implicating Hbt1p in polarized cell morphogenesis | 3,9262 | 9,987872 | 3,228995E-09 |
| AFFX-r2-Sc-18SrRNA-5_at | **---** | --- | 3,6973 | 4,49416 | 0,0002217684 |
| 1779688_at | **---** | Mitochondrial inner membrane of unknown function; similar to Tim18p and Sdh4p; expression induced by nitrogen limitation in a GLN3, GAT1-dependent manner | 3,5455 | 9,931732 | 3,549602E-09 |
| 1771509_at | **OLE1** | Fatty acid desaturase, required for monounsaturated fatty acid synthesis and for normal distribution of mitochondria | 3,5413 | 19,43031 | 1,876277E-14 |
| AFFX-18srRnaa_at | **---** | --- | 3,5236 | 4,184716 | 0,0004567521 |
| AFFX-r2-Sc18SrRNA-M_at | **---** | --- | 3,5201 | 5,250966 | 3,870677E-05 |
| 1769988_at | **DSE4** | Daughter cell-specific secreted protein with similarity to glucanases, degrades cell wall from the daughter side causing daughter to separate from mother | 3,5190 | 9,585903 | 6,41152E-09 |
| 1770576_at | **PMA2** | Plasma membrane H+-ATPase, isoform of Pma1p, involved in pumping protons out of the cell; regulator of cytoplasmic pH and plasma membrane potential | 3,5063 | 5,083239 | 5,677784E-05 |
| 1779985_at | **PHO5** | Repressible acid phosphatase (1 of 3) that also mediates extracellular nucleotide-derived phosphate hydrolysis; secretory pathway derived cell surface glycoprotein; induced by phosphate starvation and coordinately regulated by PHO4 and PHO2 | 3,4799 | 6,948595 | 9,562655E-07 |
| 1773699_at | **PHM7** | Protein of unknown function, expression is regulated by phosphate levels; green fluorescent protein (GFP)-fusion protein localizes to the cell periphery and vacuole | 3,4211 | 5,550797 | 1,964556E-05 |
| 1779627_at | **SAE3** | Meiosis specific protein involved in DMC1-dependent meiotic recombination, forms heterodimer with Mei5p; proposed to be an assembly factor for Dmc1p | 3,3771 | 4,384572 | 0,0002863447 |
| 1774402_at | **---** | Identified by gene-trapping, microarray-based expression analysis, and genome-wide homology searching | 3,3689 | 6,085602 | 6,005642E-06 |
| 1779297_at | **---** | Putative protein of unknown function | 3,3323 | 4,14392 | 0,0005024707 |
| 1770353_at | **GIT1** | Plasma membrane permease, mediates uptake of glycerophosphoinositol and glycerophosphocholine as sources of the nutrients inositol and phosphate; expression and transport rate are regulated by phosphate and inositol availability | 3,2863 | 6,028033 | 6,811771E-06 |
| 1769854_at | **CRF1** | Transcriptional corepressor involved in the regulation of ribosomal protein gene transcription via the TOR signaling pathway and protein kinase A, phosphorylated by activated Yak1p which promotes accumulation of Crf1p in the nucleus | 3,1127 | 6,142413 | 5,305822E-06 |
| AFFX-18srRnac_at | **---** | --- | 3,1114 | 4,44832 | 0,0002467755 |
| 1772785_at | **PHM8** | Protein of unknown function, expression is induced by low phosphate levels and by inactivation of Pho85p | 3,1091 | 8,361258 | 5,85237E-08 |
| 1774699_at | **PRR2** | Protein kinase with a possible role in MAP kinase signaling in the pheromone response pathway | 3,1087 | 7,490551 | 3,172359E-07 |
| 1770137_at | **---** | Putative protein of unknown function; strong increase in transcript abundance during anaerobic growth compared to aerobic growth; cells deleted for YML083C do not exhibit growth defects in anerobic or anaerobic conditions | 3,0770 | 10,49939 | 1,385598E-09 |
| 1769955_at | **CTT1** | Cytosolic catalase T, has a role in protection from oxidative damage by hydrogen peroxide | 3,0403 | 7,175784 | 5,992493E-07 |
| 1769333_at | **GRE1** | Hydrophilin of unknown function; stress induced (osmotic, ionic, oxidative, heat shock and heavy metals); regulated by the HOG pathway | 3,0235 | 3,434144 | 0,002626071 |
| 1770515_x_at | **PAU4** | Part of 23-member seripauperin multigene family encoded mainly in subtelomeric regions, active during alcoholic fermentation, regulated by anaerobiosis, negatively regulated by oxygen, repressed by heme /// Hypothetical protein | 3,0151 | 9,791729 | 4,501922E-09 |
| 1780089_at | **MAM1** | Monopolin, kinetochore associated protein involved in chromosome attachment to meiotic spindle | 2,9891 | 6,893367 | 1,072443E-06 |
| 1777444_at | **PAI3** | Cytoplasmic proteinase A inhibitor, dependent on Pbs2p and Hog1p protein kinases for osmotic induction; intrinsically unstructured, N-terminal half becomes ordered in the active site of proteinase A upon contact | 2,8646 | 6,435999 | 2,815146E-06 |
| 1778375_at | **TIS11** | mRNA-binding protein expressed during iron starvation; binds to a sequence element in the 3'-untranslated regions of specific mRNAs to mediate their degradation; involved in iron homeostasis | 2,8582 | 4,899184 | 8,668777E-05 |
| 1771052_at | **FMP45** | Integral membrane protein localized to mitochondria (untagged protein) and eisosomes, immobile patches at the cortex associated with endocytosis; sporulation and sphingolipid content are altered in mutants; has homologs SUR7 and YNL194C | 2,8517 | 4,653384 | 0,0001531228 |
| 1771067_at | **ABF1** | DNA binding protein with possible chromatin-reorganizing activity involved in transcriptional activation, gene silencing, and DNA replication and repair | 2,8130 | 11,23039 | 4,34544E-10 |
| RPTR-Sc-J01347-1_s_at | **---** | --- | 2,7997 | 9,069559 | 1,592013E-08 |
| 1777402_at | **---** | Putative protein of unknown function; proper regulation of expression during heat stress is sphingolipid-dependent | 2,7024 | 5,636056 | 1,622778E-05 |
| 1775539_at | **---** | Putative protein of unknown function; null mutant displays elevated sensitivity to expression of a mutant huntingtin fragment or of alpha-synuclein; YGL262W is not an essential gene | 2,6926 | 4,526698 | 0,0002055818 |
| 1776366_at | **PXA1** | Subunit of a heterodimeric peroxisomal ATP-binding cassette transporter complex (Pxa1p-Pxa2p), required for import of long-chain fatty acids into peroxisomes; similarity to human adrenoleukodystrophy transporter and ALD-related proteins | 2,6782 | 8,561209 | 4,026214E-08 |
| 1775854_s_at | **---** | Retrotransposon TYA Gag gene co-transcribed with TYB Pol; translated as TYA or TYA-TYB polyprotein; Gag is a nucleocapsid protein that is the structural constituent of virus-like particles (VLPs); similar to retroviral Gag /// Retrotransposon TYA Gag and TYB Pol genes; transcribed/translated as one unit; polyprotein is processed to make a nucleocapsid-like protein (Gag), reverse transcriptase (RT), protease (PR), and integrase (IN); similar to retroviral genes /// TyA Gag protein; the main structural constituent of virus-like particles (VLPs) | 2,6782 | 6,923872 | 1,006579E-06 |
| AFFX-25srRnaa_at | **---** | --- | 2,6744 | 4,062106 | 0,0006084235 |
| 1771615_at | **---** | Putative protein of unknown function; identified by fungal homology and RT-PCR | 2,6680 | 5,688375 | 1,443757E-05 |
| 1779075_at | **OPT2** | Oligopeptide transporter; member of the OPT family, with potential orthologs in S. pombe and C. albicans | 2,6611 | 6,067088 | 6,253633E-06 |
| 1777726_at | **MER1** | Protein with RNA-binding motifs required for meiosis-specific mRNA splicing; required for chromosome pairing and meiotic recombination | 2,6521 | 4,394296 | 0,0002799194 |
| 1778930_at | **---** | Hypothetical protein | 2,6501 | 8,645128 | 3,446609E-08 |
| 1780013_at | **ECI1** | Peroxisomal delta3,delta2-enoyl-CoA isomerase, hexameric protein that converts 3-hexenoyl-CoA to trans-2-hexenoyl-CoA, essential for the beta-oxidation of unsaturated fatty acids, oleate-induced | 2,6444 | 10,23675 | 2,131564E-09 |
| AFFX-18srRnad_at | **---** | --- | 2,6362 | 4,450398 | 0,0002455825 |
| 1775922_at | **ARA2** | NAD-dependent arabinose dehydrogenase, involved in biosynthesis of erythroascorbic acid; similar to plant L-galactose dehydrogenase | 2,6323 | 8,143345 | 8,849956E-08 |
| 1775937_at | **---** | Putative protein of unknown function | 2,5842 | 7,54073 | 2,870009E-07 |
| 1774003_at | **---** | Dubious open reading frame unlikely to encode a protein, based on available experimental and comparative sequence data | 2,5715 | 10,51007 | 1,361749E-09 |
| 1770887_at | **---** | Putative protein of unknown function; the authentic, non-tagged protein is detected in highly purified mitochondria in high-throughput studies | 2,5545 | 6,752117 | 1,440565E-06 |
| 1770349_at | **SWI5** | Transcription factor that activates transcription of genes expressed at the M/G1 phase boundary and in G1 phase; localization to the nucleus occurs during G1 and appears to be regulated by phosphorylation by Cdc28p kinase | 2,5543 | 6,564699 | 2,139706E-06 |
| 1777581_at | **---** | Putative protein of unknown function; identified by fungal homology and RT-PCR | 2,5486 | 3,815813 | 0,001081856 |
| 1779309_at | **ARG1** | Arginosuccinate synthetase, catalyzes the formation of L-argininosuccinate from citrulline and L-aspartate in the arginine biosynthesis pathway; potential Cdc28p substrate | 2,5408 | 8,546284 | 4,139463E-08 |
| 1778197_at | **MND1** | Protein required for recombination and meiotic nuclear division; forms a complex with Hop2p, which is involved in chromosome pairing and repair of meiotic double-strand breaks | 2,5314 | 5,500544 | 2,199627E-05 |
| 1773702_at | **---** | Protein of unknown function with similarity to succinate dehydrogenase cytochrome b subunit; YMR118C is not an essential gene | 2,5295 | 5,07578 | 5,775688E-05 |
| 1772377_at | **MEI5** | Meiosis specific protein involved in DMC1-dependent meiotic recombination, forms heterodimer with Sae3p; proposed to be an assembly factor for Dmc1p | 2,5212 | 5,864441 | 9,764552E-06 |
| 1771376_at | **BNA5** | Kynureninase, required for biosynthesis of nicotinic acid from tryptophan via kynurenine pathway | 2,5085 | 9,287848 | 1,079535E-08 |
| 1772833_at | **MGA2** | ER membrane protein involved in regulation of OLE1 transcription, acts with homolog Spt23p; inactive ER form dimerizes and one subunit is then activated by ubiquitin/proteasome-dependent processing followed by nuclear targeting | 2,4944 | 6,894644 | 1,069597E-06 |
| AFFX-r2-Sp-18SrRNA-5_at | **---** | --- | 2,4926 | 4,320435 | 0,0003326024 |
| 1777808_at | **GIP1** | Meiosis-specific regulatory subunit of the Glc7p protein phosphatase, regulates spore wall formation and septin organization, required for expression of some late meiotic genes and for normal localization of Glc7p | 2,4787 | 8,360861 | 5,856747E-08 |
| 1777779_at | **CSM4** | Protein required for accurate chromosome segregation during meiosis | 2,4771 | 8,167657 | 8,448269E-08 |
| 1769622_at | **---** | Putative protein of unknown function | 2,4667 | 5,468514 | 2,364251E-05 |
| 1773617_at | **---** | Putative protein of unknown function; YCL001W-A is not an essential gene | 2,4629 | 4,603133 | 0,0001720869 |
| 1776208_at | **---** | Putative protein of unknown function | 2,4618 | 4,685373 | 0,0001421646 |
| 1769657_at | **DSE3** | Daughter cell-specific protein, may help establish daughter fate | 2,4568 | 7,680686 | 2,174382E-07 |
| 1780148_at | **---** | Glyoxylate reductase; acts on glyoxylate and hydroxypyruvate substrates; YPL113C is not an essential gene | 2,4552 | 5,294901 | 3,50257E-05 |
| 1776283_at | **IZH4** | Membrane protein involved in zinc metabolism, member of the four-protein IZH family, expression induced by fatty acids and altered zinc levels; deletion reduces sensitivity to excess zinc; possible role in sterol metabolism | 2,4517 | 5,584875 | 1,819894E-05 |
| 1780070_at | **ZTA1** | Zeta-crystallin homolog, found in the cytoplasm and nucleus; has similarity to E. coli quinone oxidoreductase and to human zeta-crystallin, which has quinone oxidoreductase activity | 2,4338 | 6,001495 | 7,219974E-06 |
| AFFX-r2-Sp-28SrRNA-3_at | **---** | --- | 2,4258 | 6,812733 | 1,268802E-06 |
| 1776346_at | **HEM15** | Ferrochelatase, a mitochondrial inner membrane protein, catalyzes the insertion of ferrous iron into protoporphyrin IX, the eighth and final step in the heme biosynthetic pathway | 2,4132 | 8,646018 | 3,440949E-08 |
| 1772019_at | **BNA1** | 3-hydroxyanthranilic acid dioxygenase, required for biosynthesis of nicotinic acid from tryptophan via kynurenine pathway | 2,4093 | 8,069653 | 1,019284E-07 |
| 1772391_at | **---** | Putative protein of unknown function; expression induced under carbon limitation and repressed under high glucose | 2,4029 | 6,534954 | 2,279334E-06 |
| 1774240_at | **SPR3** | Sporulation-specific homolog of the yeast CDC3/10/11/12 family of bud neck microfilament genes; septin protein involved in sporulation; regulated by ABFI | 2,3886 | 7,064478 | 7,527876E-07 |
| 1776251_at | **---** | Putative protein of unknown function; identified by gene-trapping, microarray-based expression analysis, and genome-wide homology searching | 2,3815 | 5,42143 | 2,629391E-05 |
| 1778563_at | **---** | Putative alanine transaminase (glutamic pyruvic transaminase) | 2,3749 | 6,662243 | 1,740511E-06 |
| 1773293_at | **---** | Dubious open reading frame unlikely to encode a protein, based on available experimental and comparative sequence data /// Putative protein of unknown function; identified by gene-trapping, microarray-based expression analysis, and genome-wide homology searching | 2,3693 | 4,452489 | 0,000244388 |
| 1775439_at | **ETR1** | 2-enoyl thioester reductase, member of the medium chain dehydrogenase/reductase family; localized to in mitochondria, where it has a probable role in fatty acid synthesis | 2,3684 | 5,208359 | 4,265305E-05 |
| 1769792_at | **---** | Putative protein of unknown function; deletion mutant is viable and has no detectable phenotype | 2,3625 | 8,052711 | 1,05304E-07 |
| 1776763_at | **CLN2** | G1 cyclin involved in regulation of the cell cycle; activates Cdc28p kinase to promote the G1 to S phase transition; late G1 specific expression depends on transcription factor complexes, MBF (Swi6p-Mbp1p) and SBF (Swi6p-Swi4p) | 2,3507 | 8,616739 | 3,632319E-08 |
| 1769585_at | **---** | Putative protein of unknown function; YBR285W is not an essential gene and deletion of YBR285W leads to poor growth on glucose-minimal medium at 15C | 2,3479 | 4,155597 | 0,0004889361 |
| 1779165_s_at | **---** | Retrotransposon TYA Gag and TYB Pol genes; in YARCTY1-1 TYB is mutant and probably non-functional /// Retrotransposon TYA Gag and TYB Pol genes; transcribed/translated as one unit; polyprotein is processed to make a nucleocapsid-like protein (Gag), reverse transcriptase (RT), protease (PR), and integrase (IN); similar to retroviral genes | 2,3387 | 4,147563 | 0,0004982083 |
| 1777149_at | **---** | Putative protein of unknown function | 2,3383 | 7,014147 | 8,350362E-07 |
| 1770015_at | **---** | Putative protein of unknown function | 2,3369 | 3,855086 | 0,0009870775 |
| 1773674_at | **---** | Putative protein of unknown function; deletion mutant has no readily detectable phenotype | 2,3300 | 4,659559 | 0,0001509431 |
| 1770892_at | **---** | Cytoplasmic protein of unknown function, potentially phosphorylated by Cdc28p; YBR138C is not an essential gene | 2,3295 | 5,297443 | 3,482393E-05 |
| 1772915_at | **ACS1** | Acetyl-coA synthetase isoform which, along with Acs2p, is the nuclear source of acetyl-coA for histone acetlyation; expressed during growth on nonfermentable carbon sources and under aerobic conditions | 2,3258 | 7,372443 | 4,021132E-07 |
| 1770447_s_at | **YRF1-1 /// YRF1-2 /// YRF1-3 /// YRF1-4 /// YRF1-5 /// YRF1-6 /// YRF1-7** | Helicase encoded by the Y' element of subtelomeric regions, highly expressed in the mutants lacking the telomerase component TLC1; potentially phosphorylated by Cdc28p /// Putative protein of unknown function | 2,3250 | 3,375956 | 0,003003258 |
| 1773966_at | **YPT53** | GTPase, similar to Ypt51p and Ypt52p and to mammalian rab5; required for vacuolar protein sorting and endocytosis | 2,3212 | 7,297031 | 4,682948E-07 |
| 1778852_at | **SMA1** | Protein of unknown function involved in the assembly of the prospore membrane during sporulation | 2,3096 | 4,321498 | 0,0003317771 |
| 1769325_at | **RRI1** | Catalytic subunit of the COP9 signalosome (CSN) complex that acts as an isopeptidase in cleaving the ubiquitin-like protein Nedd8 from SCF ubiquitin ligases; metalloendopeptidase involved in the adaptation to pheromone signaling | 2,2802 | 7,889866 | 1,443089E-07 |
| 1776304_at | **CTA1** | Catalase A, breaks down hydrogen peroxide in the peroxisomal matrix formed by acyl-CoA oxidase (Pox1p) during fatty acid beta-oxidation | 2,2772 | 6,853951 | 1,164181E-06 |
| 1770587_at | **---** | Putative protein of unknown function | 2,2718 | 7,194261 | 5,770756E-07 |
| 1771084_at | **MCM10** | Essential chromatin-associated protein involved in the initiation of DNA replication; required for the association of the MCM2-7 complex with replication origins | 2,2695 | 5,420331 | 2,635931E-05 |
| 1775917_at | **LAP3** | Cysteine aminopeptidase with homocysteine-thiolactonase activity; protects cells against homocysteine toxicity; has bleomycin hydrolase activity in vitro; transcription is regulated by galactose via Gal4p; orthologous to human BLMH | 2,2542 | 7,353776 | 4,175395E-07 |
| AFFX-r2-Sc-18SrRNA-3_at | **---** | --- | 2,2526 | 6,894061 | 1,070895E-06 |
| 1777937_at | **---** | Putative protein of unknown function | 2,2482 | 7,675626 | 2,196211E-07 |
| 1770913_at | **---** | Putative protein of unknown function; identified by fungal homology and RT-PCR | 2,2372 | 6,718975 | 1,544438E-06 |
| 1771072_at | **ATG16** | Protein that interacts with the Atg12p-Atg5p conjugate during formation of the pre-autophagosomal structure; essential for autophagy | 2,2317 | 5,302117 | 3,44561E-05 |
| 1778200_at | **---** | Putative protein of unknown function; induced in respiratory-deficient cells | 2,2218 | 6,83165 | 1,219633E-06 |
| 1776143_at | **STH1** | ATPase component of the RSC chromatin remodeling complex; required for expression of early meiotic genes; essential helicase-related protein homologous to Snf2p | 2,2148 | 6,642963 | 1,812836E-06 |
| 1774245_at | **---** | Putative protein of unknown function; identified by homology | 2,2136 | 4,065723 | 0,000603298 |
| 1779645_at | **DBP2** | Essential ATP-dependent RNA helicase of the DEAD-box protein family, involved in nonsense-mediated mRNA decay and rRNA processing | 2,2016 | 3,721412 | 0,001348268 |
| 1769466_at | **KNH1** | Protein with similarity to Kre9p, which is involved in cell wall beta 1,6-glucan synthesis; overproduction suppresses growth defects of a kre9 null mutant | 2,1952 | 7,409942 | 3,728794E-07 |
| 1772496_at | **ANT1** | Peroxisomal adenine nucleotide transporter; involved in beta-oxidation of medium-chain fatty acid; required for peroxisome proliferation | 2,1786 | 8,265194 | 7,017401E-08 |
| 1774893_at | **---** | Putative protein of unknown function; identified by fungal homology and RT-PCR | 2,1775 | 3,435342 | 0,002618813 |
| 1777553_s_at | **YRF1-1 /// YRF1-2 /// YRF1-3 /// YRF1-5 /// YRF1-6 /// YRF1-7** | Helicase-like protein encoded within the telomeric Y' element /// Helicase encoded by the Y' element of subtelomeric regions, highly expressed in the mutants lacking the telomerase component TLC1; potentially phosphorylated by Cdc28p /// Putative protein of unknown function /// Putative protein of unknown function; similarity to DNA helicases that are encoded within subtelomeric Y' elements and induced in telomerase deficient survivors /// Putative protein of unknown function; similarity to DNA helicases that are also encoded within subtelomeric Y' elements and induced in telomerase deficient survivors /// Putative protein of unknown function with similarity to helicases; YLL066C is not an essential gene /// Putative protein of unknown function with similarity to helicases /// Putative protein of unknown function with similarity to helicases; the authentic, non-tagged protein is detected in highly purified mitochondria in high-throughput studies; YML133C contains an intron | 2,1768 | 11,01758 | 6,055093E-10 |
| 1779266_at | **RTN2** | Protein of unknown function; has similarity to mammalian reticulon proteins; member of the RTNLA (reticulon-like A) subfamily | 2,1710 | 4,067564 | 0,0006007073 |
| 1771880_at | **DBR1** | RNA lariat debranching enzyme, involved in intron turnover; required for efficient Ty1 transposition | 2,1682 | 5,614219 | 1,704063E-05 |
| 1777690_at | **XBP1** | Transcriptional repressor that binds to promoter sequences of the cyclin genes, CYS3, and SMF2; expression is induced by stress or starvation during mitosis, and late in meiosis; member of the Swi4p/Mbp1p family; potential Cdc28p substrate | 2,1673 | 6,502392 | 2,442974E-06 |
| 1773363_x_at | **---** | Dubious open reading frame unlikely to encode a protein, based on available experimental and comparative sequence data | 2,1663 | 9,52625 | 7,110107E-09 |
| 1776680_at | **SPG1** | Protein required for survival at high temperature during stationary phase; not required for growth on nonfermentable carbon sources; the authentic, non-tagged protein is detected in highly purified mitochondria in high-throughput studies | 2,1596 | 3,901912 | 0,0008848091 |
| 1778142_at | **TKL2** | Transketolase, similar to Tkl1p; catalyzes conversion of xylulose-5-phosphate and ribose-5-phosphate to sedoheptulose-7-phosphate and glyceraldehyde-3-phosphate in the pentose phosphate pathway; needed for synthesis of aromatic amino acids | 2,1590 | 2,816481 | 0,01066103 |
| 1776156_at | **BDH2** | Putative medium-chain alcohol dehydrogenase with similarity to BDH1; transcription induced by constitutively active PDR1 and PDR3; BDH2 is an essential gene | 2,1561 | 6,623708 | 1,888159E-06 |
| 1778380_at | **MGA1** | Protein similar to heat shock transcription factor; multicopy suppressor of pseudohyphal growth defects of ammonium permease mutants | 2,1487 | 3,864266 | 0,0009661427 |
| 1776583_at | **FIT2** | Mannoprotein that is incorporated into the cell wall via a glycosylphosphatidylinositol (GPI) anchor, involved in the retention of siderophore-iron in the cell wall | 2,1458 | 6,370695 | 3,238281E-06 |
| 1775046_at | **FAS2** | Alpha subunit of fatty acid synthetase, which catalyzes the synthesis of long-chain saturated fatty acids; contains beta-ketoacyl reductase and beta-ketoacyl synthase activities | 2,1415 | 9,631539 | 5,925491E-09 |
| 1773641_x_at | **---** | Hypothetical protein | 2,1378 | 4,276971 | 0,0003681502 |
| 1779806_at | **OSW1** | Protein involved in sporulation; required for the construction of the outer spore wall layers; required for proper localization of Spo14p | 2,1240 | 2,406684 | 0,02588195 |
| 1771313_at | **HMG1** | One of two isozymes of HMG-CoA reductase that catalyzes the conversion of HMG-CoA to mevalonate, which is a rate-limiting step in sterol biosynthesis; localizes to the nuclear envelope; overproduction induces the formation of karmellae | 2,1054 | 6,578104 | 2,079685E-06 |
| 1773376_at | **REC114** | Protein involved in early stages of meiotic recombination; possibly involved in the coordination of recombination and meiotic division; mutations lead to premature initiation of the first meiotic division | 2,1001 | 2,266864 | 0,0346406 |
| 1777924_at | **---** | Putative protein of unknown function; identified by expression profiling and mass spectrometry | 2,0989 | 2,540598 | 0,01946583 |
| 1776576_at | **MBR1** | Protein involved in mitochondrial functions and stress response; overexpression suppresses growth defects of hap2, hap3, and hap4 mutants | 2,0975 | 6,592793 | 2,015906E-06 |
| 1774770_at | **ENT5** | Protein containing an N-terminal epsin-like domain involved in clathrin recruitment and traffic between the Golgi and endosomes; associates with the clathrin adaptor Gga2p, clathrin adaptor complex AP-1, and clathrin | 2,0943 | 5,615084 | 1,700767E-05 |
| 1777872_at | **---** | Putative protein of unknown function with some characteristics of a transcriptional activator; may be a target of Dbf2p-Mob1p kinase; GFP-fusion protein co-localizes with clathrin-coated vesicles; YML037C is not an essential gene | 2,0929 | 6,428442 | 2,861057E-06 |
| 1769426_at | **PDH1** | Mitochondrial protein that participates in respiration, induced by diauxic shift; homologous to E. coli PrpD, may take part in the conversion of 2-methylcitrate to 2-methylisocitrate | 2,0898 | 9,945174 | 3,469934E-09 |
| AFFX-r2-Sc-25SrRNA-5_at | **---** | --- | 2,0865 | 2,90311 | 0,008792771 |
| 1775970_at | **---** | Dubious open reading frame unlikely to encode a protein, based on available experimental and comparative sequence data; completely overlaps the characterized snoRNA gene snR73 | 2,0764 | 2,767758 | 0,01187285 |
| 1775289_at | **SAC7** | GTPase activating protein (GAP) for Rho1p, involved in signaling to the actin cytoskeleton, null mutations suppress tor2 mutations and temperature sensitive mutations in actin; potential Cdc28p substrate | 2,0727 | 4,897584 | 8,700823E-05 |
| 1776931_x_at | **PAU6** | Hypothetical protein /// Part of 23-member seripauperin multigene family encoded mainly in subtelomeric regions, active during alcoholic fermentation, regulated by anaerobiosis, negatively regulated by oxygen, repressed by heme | 2,0638 | 6,366036 | 3,270858E-06 |
| 1769767_at | **crf** | Protein required for accurate chromosome segregation during meiosis | 2,0638 | 4,943449 | 7,828054E-05 |
| 1773369_at | **ALO1** | D-Arabinono-1,4-lactone oxidase, catalyzes the final step in biosynthesis of D-erythroascorbic acid, which is protective against oxidative stress | 2,0584 | 6,660565 | 1,746684E-06 |
| 1770966_at | **---** | Protein of unknown function; essential for growth under anaerobic conditions; mutation causes decreased expression of ATP2, impaired respiration, defective sterol uptake, and altered levels/localization of ABC transporters Aus1p and Pdr11p | 2,0450 | 4,507137 | 0,0002151638 |
| 1776164_at | **HUL4** | Protein with similarity to hect domain E3 ubiquitin-protein ligases, not essential for viability | 2,0418 | 8,615735 | 3,639072E-08 |
| 1776290_at | **---** | Putative protein of unknown function; green fluorescent protein (GFP)-fusion protein localizes to the vacuole | 2,0313 | 5,754946 | 1,244789E-05 |
| 1770128_at | **HAP1** | Zinc finger transcription factor involved in the complex regulation of gene expression in response to levels of heme and oxygen; the S288C sequence differs from other strain backgrounds due to a Ty1 insertion in the carboxy terminus | 2,0304 | 4,695791 | 0,0001387696 |
| 1777133_at | **MDG1** | Plasma membrane protein involved in G-protein mediated pheromone signaling pathway; overproduction suppresses bem1 mutations | 2,0302 | 5,949964 | 8,085751E-06 |
| 1777228_at | **---** | Putative protein of unknown function | 2,0253 | 3,42872 | 0,002659164 |
| 1780221_at | **RME1** | Zinc finger protein involved in control of meiosis; prevents meiosis by repressing IME1 expression and promotes mitosis by activating CLN2 expression; directly repressed by a1-a2 regulator; mediates cell type control of sporulation | 2,0206 | 6,171989 | 4,975181E-06 |
| 1779965_at | **DSE2** | Daughter cell-specific secreted protein with similarity to glucanases, degrades cell wall from the daughter side causing daughter to separate from mother; expression is repressed by cAMP | 2,0181 | 6,284039 | 3,902808E-06 |
| 1778753_at | **---** | Putative protein of unknown function | 2,0153 | 4,054751 | 0,0006189779 |
| 1769973_at | **CLB1** | B-type cyclin involved in cell cycle progression; activates Cdc28p to promote the transition from G2 to M phase; accumulates during G2 and M, then targeted via a destruction box motif for ubiquitin-mediated degradation by the proteasome | 2,0145 | 5,621033 | 1,678264E-05 |
| 1778270_at | **HO** | Site-specific endonuclease required for gene conversion at the MAT locus (homothallic switching) through the generation of a ds DNA break; expression restricted to mother cells in late G1 as controlled by Swi4p-Swi6p, Swi5p and Ash1p | 2,0132 | 3,072597 | 0,006006426 |
| 1777587_at | **---** | Identified by gene-trapping, microarray-based expression analysis, and genome-wide homology searching | 2,0130 | 3,682536 | 0,001476035 |
| 1770123_at | **---** | Putative protein of unknown function, similar to bacterial galactoside O-acetyltransferases; induced by oleate in an OAF1/PIP2-dependent manner; promoter contains an oleate response element consensus sequence; non-essential gene | 2,0113 | 4,449105 | 0,0002463239 |
| 1779705_at | **---** | Putative protein of unknown function; GFP-fusion protein is induced in response to the DNA-damaging agent MMS; the authentic, non-tagged protein is detected in highly purified mitochondria in high-throughput studies | 2,0085 | 3,770775 | 0,001201721 |
| 1772828_at | **BUD22** | Protein involved in bud-site selection; diploid mutants display a random budding pattern instead of the wild-type bipolar pattern | 2,0076 | 4,518066 | 0,0002097562 |
| 1777343_at | **SPS19** | Peroxisomal 2,4-dienoyl-CoA reductase, auxiliary enzyme of fatty acid beta-oxidation; homodimeric enzyme required for growth and sporulation on petroselineate medium; expression induced during late sporulation and in the presence of oleate | 2,0066 | 5,20827 | 4,266172E-05 |
| 1777175_at | **MEP1** | Ammonium permease; belongs to a ubiquitous family of cytoplasmic membrane proteins that transport only ammonium (NH4+); expression is under the nitrogen catabolite repression regulation | 2,0016 | 4,541307 | 0,0001987068 |
