# Supplementary material for: Agp2, a Member of the Yeast Amino Acid Permease Family, Positively Regulates Polyamine Transport at the Transcriptional Level
Source: PLoS One. 2013 Jun 3;8(6):e65717. doi: 10.1371/journal.pone.0065717 (PMC3670898; doi:10.1371/journal.pone.0065717)
Supplement: Table S3 — Functional category of downregulated genes in the absence of Agp2. (DOCX) [file pone.0065717.s004.docx]

**Table S3:** **Functional category of downregulated genes in the absence of Agp2**

| Function | ORF | Gene Name | Fold change |
| --- | --- | --- | --- |
| Transmembrane transport |  |  |  |
| ATP binding protein involved in protein folding and vacuolar import of proteins; member of heat shock protein 70 (HSP70) family; associated with the chaperonin-containing T-complex; present in the cytoplasm, vacuolar membrane and cell wall. | YLL024C | SSA2 | 11 |
| Putative purine-cytosine permease, very similar to Fcy2p but cannot substitute for its function | YER060W-A | FCY22 | 5.6 |
| Putative channel-like protein; similar to Fps1p; mediates passive diffusion of glycerol in the presence of ethanol |  | ----- | 15.5 |
| Low-affinity glucose transporter of the major facilitator superfamily, expression is induced by Hxk2p in the presence of glucose and repressed by Rgt1p when glucose is limiting | YHR094C | HXT1 | 6.08 |
| Low affinity glucose transporter of the major facilitator superfamily, expression is induced in low or high glucose conditions | YDR345C | HXT3 | 7.7 |
| Uracil permease, localized to the plasma membrane; expression is tightly regulated by uracil levels and environmental cues | YBR021W | FUR4 | 6.5 |
| High-affinity glucose transporter of the major facilitator superfamily, expression is induced by low levels of glucose and repressed by high levels of glucose | YMR011W | HXT2 | 4.4 |
| Alpha-glucoside permease, transports maltose, maltotriose, alpha-methylglucoside, and turanose; identical to Mph3p; encoded in a subtelomeric position in a region likely to have undergone duplication | YDL247W | MPH2 | 6.2 |
| Plasma membrane riboflavin transporter; facilitates the uptake of vitamin B2; required for FAD-dependent processes; sequence similarity to mammalian monocarboxylate permeases, however mutants are not deficient in monocarboxylate transport | YOR306C | MCH5 | 9.6 |
| Hexose transporter, induced in the presence of non-fermentable carbon sources, induced by low levels of glucose, repressed by high levels of glucose | YEL069C | HXT13 | 7 |
| High affinity sulfate permease; sulfate uptake is mediated by specific sulfate transporters Sul1p and Sul2p, which control the concentration of endogenous activated sulfate intermediates | YBR294W | SUL1 | 3.5 |
| High-affinity plasma membrane H+-biotin (vitamin H) symporter; mutation results in fatty acid auxotrophy; 12 transmembrane domain containing major facilitator subfamily member; mRNA levels negatively regulated by iron deprivation and biotin | YGR065C | VHT1 | 8.3 |
| Plasma membrane transporter responsible for the uptake of thiamine, member of the major facilitator superfamily of transporters; mutation of human ortholog causes thiamine-responsive megaloblastic anemia | YLR237W | THI7 | 7.5 |
| Plasma membrane glucose receptor, highly similar to Snf3p; both Rgt2p and Snf3p serve as transmembrane glucose sensors generating an intracellular signal that induces expression of glucose transporter (HXT) genes | YDL138W | RGT2 | 6 |
| Ferric reductase and cupric reductase, reduces siderophore-bound iron and oxidized copper prior to uptake by transporters; expression induced by low copper and iron levels | YLR214W | FRE1 | 5.3 |
| Alpha-glucoside permease, transports maltose, maltotriose, alpha-methylglucoside, and turanose; identical to Mph3p; encoded in a subtelomeric position in a region likely to have undergone duplication /// Alpha-glucoside permease, transports maltose, maltotriose, alpha-methylglucoside, and turanose; identical to Mph2p; encoded in a subtelomeric position in a region likely to have undergone duplication | YDL247W | MPH2 /// MPH3 | 3.2 |
| Nitrogen compound biosynthetic pathway |  |  |  |
| Dihydroorotate dehydrogenase, catalyzes the fourth enzymatic step in the de novo biosynthesis of pyrimidines, converting dihydroorotic acid into orotic acid | YKL216W | URA1 | 37.5 |
| Inosine monophosphate dehydrogenase, catalyzes the first step of GMP biosynthesis, expression is induced by mycophenolic acid resulting in resistance to the drug, expression is repressed by nutrient limitation | YHR216W | IMD2 | 5.2 |
| Protein with similarity to hydroxymethylpyrimidine phosphate kinases; member of a gene family with THI20 and THI21; not required for thiamine biosynthesis | YPR121W | THI22 | 7.9 |
| Putative cystathionine beta-lyase; involved in copper ion homeostasis and sulfur metabolism; null mutant displays increased levels of spontaneous Rad52 foci; expression induced by nitrogen limitation in a GLN3, GAT1-dependent manner |  | ----- | 7.6 |
| Cystathionine gamma-lyase, catalyzes one of the two reactions involved in the transsulfuration pathway that yields cysteine from homocysteine with the intermediary formation of cystathionine | YAL012W | CYS3 | 4.5 |
| Saccharopine dehydrogenase (NAD+, L-lysine-forming), catalyzes the conversion of saccharopine to L-lysine, which is the final step in the lysine biosynthesis pathway | YIR034C | LYS1 | 7.2 |
| 7,8-diamino-pelargonic acid aminotransferase (DAPA), catalyzes the second step in the biotin biosynthesis pathway; BIO3 is in a cluster of 3 genes (BIO3, BIO4, and BIO5) that mediate biotin synthesis | YNR058W | BIO3 | 9.8 |
| Putative protein of unknown function; YBR284W is not an essential gene; null mutant exhibits decreased resistance to rapamycin and wortmannin |  | ---- | 5.7 |
| Lumazine synthase (6,7-dimethyl-8-ribityllumazine synthase, also known as DMRL synthase); catalyzes synthesis of immediate precursor to riboflavin | YOL143C | RIB4 | 9.7 |
| Homoaconitase, catalyzes the conversion of homocitrate to homoisocitrate, which is a step in the lysine biosynthesis pathway | *YDR234W* | LYS4 | 7.3 |
| Spermine synthase, required for the biosynthesis of spermine and also involved in biosynthesis of pantothenic acid | YLR146C | SPE4 | 9.6 |
| Thiazole synthase, catalyzes formation of the thiazole moiety of thiamin pyrophosphate; required for thiamine biosynthesis and for mitochondrial genome stability | YGR144W | THI4 | 5.8 |
| DRAP deaminase, catalyzes the third step of the riboflavin biosynthesis pathway; cytoplasmic tRNA pseudouridine synthase involved in pseudouridylation of cytoplasmic tRNAs at position 32  Vitamin biosynthetic process | YOL066C | RIB2 | 9.6 |
| Protein with similarity to hydroxymethylpyrimidine phosphate kinases; member of a gene family with THI20 and THI21; not required for thiamine biosynthesis | YPR121W | THI22 | 7.9 |
| 7,8-diamino-pelargonic acid aminotransferase (DAPA), catalyzes the second step in the biotin biosynthesis pathway; BIO3 is in a cluster of 3 genes (BIO3, BIO4, and BIO5) that mediate biotin synthesis | YNR058W | BIO3 | 9.8 |
| Lumazine synthase (6,7-dimethyl-8-ribityllumazine synthase, also known as DMRL synthase); catalyzes synthesis of immediate precursor to riboflavin | YOL143C | RIB4 | 9.7 |
| Thiazole synthase, catalyzes formation of the thiazole moiety of thiamin pyrophosphate; required for thiamine biosynthesis and for mitochondrial genome stability | YGR144W | THI4 | 5.8 |
| DRAP deaminase, catalyzes the third step of the riboflavin biosynthesis pathway; cytoplasmic tRNA pseudouridine synthase involved in pseudouridylation of cytoplasmic tRNAs at position 32 | YOL066C | RIB2 | 9.6 |
| cell surface receptor linked signal transduction |  |  |  |
| Mating pheromone a-factor, made by a cells; interacts with alpha cells to induce cell cycle arrest and other responses leading to mating; biogenesis involves C-terminal modification, N-terminal proteolysis, and export; also encoded by MFA2 | YDR461W | MFA1 | 8.7 |
| MAP kinase kinase kinase of the HOG1 mitogen-activated signaling pathway; functionally redundant with, and homologous to, Ssk2p; interacts with and is activated by Ssk1p; phosphorylates Pbs2p | YCR073C | SSK22 | 12.1 |
| Receptor for alpha-factor pheromone; seven transmembrane-domain GPCR that interacts with both pheromone and a heterotrimeric G protein to initiate the signaling response that leads to mating between haploid a and alpha cells | YFL026W | STE2 | 7.1 |
| Mating pheromone alpha-factor, made by alpha cells; interacts with mating type a cells to induce cell cycle arrest and other responses leading to mating; also encoded by MF(ALPHA)2, although MF(ALPHA)1 produces most alpha-factor | YPL187W | MF(ALPHA)1 | 4.8 |
| Mitogen-activated protein kinase involved in osmoregulation via three independent osmosensors; mediates the recruitment and activation of RNA Pol II at Hot1p-dependent promoters; localization regulated by Ptp2p and Ptp3p | YLR113W | HOG1 | 6.7 |
| Negative regulator of glucose-induced cAMP signaling; directly activates the GTPase activity of the heterotrimeric G protein alpha subunit Gpa2p | YOR107W | RGS2 | 5.6 |
| Response to pheromone |  |  |  |
| Integral membrane protein required for efficient mating; may participate in or regulate the low affinity Ca2+ influx system, which affects intracellular signaling and cell-cell fusion during mating | YBR040W | FIG1 | 9.9 |
| Pheromone-regulated multispanning membrane protein involved in membrane fusion during mating; predicted to have 5 transmembrane segments and a coiled coil domain; localizes to the shmoo tip; regulated by Ste12p | YNL279W | PRM1 | 22 |
| Pheromone-regulated protein, predicted to have 2 transmembrane segments; regulated by Ste12p during mating | YML047C | PRM6 | 15.2 |
| Anchorage subunit of a-agglutinin of a-cells, highly O-glycosylated protein with N-terminal secretion signal and C-terminal signal for addition of GPI anchor to cell wall, linked to adhesion subunit Aga2p via two disulfide bonds | YNR044W | AGA1 | 12.6 |
| Pheromone-regulated protein, predicted to have 1 transmembrane segment; induced during cell integrity signaling | YIL117C | PRM5 | 15.8 |
| Mating pheromone a-factor, made by a cells; interacts with alpha cells to induce cell cycle arrest and other responses leading to mating; biogenesis involves C-terminal modification, N-terminal proteolysis, and export; also encoded by MFA2 | YDR461W | MFA1 | 8.7 |
| Adhesion subunit of a-agglutinin of a-cells, C-terminal sequence acts as a ligand for alpha-agglutinin (Sag1p) during agglutination, modified with O-linked oligomannosyl chains, linked to anchorage subunit Aga1p via two disulfide bonds | YGL032C | AGA2 | 7.3 |
| Pheromone-regulated protein, predicted to have one transmembrane segment; promoter contains Gcn4p binding elements | YDL039C | PRM7 | 7.6 |
| Membrane protein localized to the shmoo tip, required for cell fusion; expression regulated by mating pheromone; proposed to coordinate signaling, fusion, and polarization events required for fusion; potential Cdc28p substrate | YCL027W | FUS1 | 7.1 |
| Receptor for alpha-factor pheromone; seven transmembrane-domain GPCR that interacts with both pheromone and a heterotrimeric G protein to initiate the signaling response that leads to mating between haploid a and alpha cells | YFL026W | STE2 | 7.1 |
| Pheromone-regulated protein, predicted to have 5 transmembrane segments | YJL108C | PRM10 | 4.1 |
| Mating pheromone alpha-factor, made by alpha cells; interacts with mating type a cells to induce cell cycle arrest and other responses leading to mating; also encoded by MF(ALPHA)2, although MF(ALPHA)1 produces most alpha-factor | YPL187W | MF(ALPHA)1 | 4.8 |
| Transcription factor required for gene regulation in repsonse to pheromones; also required during meiosis; exists in two forms, a slower-migrating form more abundant during vegetative growth and a faster-migrating form induced by pheromone | YCL055W | KAR4 | 6.5 |
| Mitogen-activated protein kinase involved in osmoregulation via three independent osmosensors; mediates the recruitment and activation of RNA Pol II at Hot1p-dependent promoters; localization regulated by Ptp2p and Ptp3p | YLR113W | HOG1 | 6.7 |
| Sexual reproduction Cellular process |  |  |  |
| Integral membrane protein required for efficient mating; may participate in or regulate the low affinity Ca2+ influx system, which affects intracellular signaling and cell-cell fusion during mating | YBR040W | FIG1 | 9.9 |
| Pheromone-regulated multispanning membrane protein involved in membrane fusion during mating; predicted to have 5 transmembrane segments and a coiled coil domain; localizes to the shmoo tip; regulated by Ste12p | YNL279W | PRM1 | 22 |
| Pheromone-regulated protein, predicted to have 2 transmembrane segments; regulated by Ste12p during mating | YML047C | PRM6 | 15.2 |
| Anchorage subunit of a-agglutinin of a-cells, highly O-glycosylated protein with N-terminal secretion signal and C-terminal signal for addition of GPI anchor to cell wall, linked to adhesion subunit Aga2p via two disulfide bonds | YNR044W | AGA1 | 12.6 |
| Pheromone-regulated protein, predicted to have 1 transmembrane segment; induced during cell integrity signaling | YIL117C | PRM5 | 15.8 |
| Mating pheromone a-factor, made by a cells; interacts with alpha cells to induce cell cycle arrest and other responses leading to mating; biogenesis involves C-terminal modification, N-terminal proteolysis, and export; also encoded by MFA2 | YDR461W | MFA1 | 8.7 |
| 1,3-beta-glucanosyltransferase, involved with Gas4p in spore wall assembly; has similarity to Gas1p | YLR343W | GAS2 | 6.4 |
| Adhesion subunit of a-agglutinin of a-cells, C-terminal sequence acts as a ligand for alpha-agglutinin (Sag1p) during agglutination, modified with O-linked oligomannosyl chains, linked to anchorage subunit Aga1p via two disulfide bonds | YGL032C | AGA2 | 7.3 |
| Pheromone-regulated protein, predicted to have one transmembrane segment; promoter contains Gcn4p binding elements | YDL039C | PRM7 | 7.6 |
| Membrane protein localized to the shmoo tip, required for cell fusion; expression regulated by mating pheromone; proposed to coordinate signaling, fusion, and polarization events required for fusion; potential Cdc28p substrate | YCL027W | FUS1 | 7.1 |
| Protein of unknown function involved in the assembly of the prospore membrane during sporulation | YML066C | SMA2 | 3.3 |
| Receptor for alpha-factor pheromone; seven transmembrane-domain GPCR that interacts with both pheromone and a heterotrimeric G protein to initiate the signaling response that leads to mating between haploid a and alpha cells | YFL026W | STE2 | 7.1 |
| Alpha-tubulin; associates with beta-tubulin (Tub2p) to form tubulin dimer, which polymerizes to form microtubules; expressed at lower level than Tub1p | YML124C | TUB3 | 6.3 |
| Pheromone-regulated protein, predicted to have 5 transmembrane segments | YJL108C | PRM10 | 4.1 |
| Mating pheromone alpha-factor, made by alpha cells; interacts with mating type a cells to induce cell cycle arrest and other responses leading to mating; also encoded by MF(ALPHA)2, although MF(ALPHA)1 produces most alpha-factor | YPL187W | MF(ALPHA)1 | 4.8 |
| Transcription factor required for gene regulation in repsonse to pheromones; also required during meiosis; exists in two forms, a slower-migrating form more abundant during vegetative growth and a faster-migrating form induced by pheromone | YCL055W | KAR4 | 6.5 |
| Protein involved in shmoo formation and bipolar bud site selection; homologous to Spa2p, localizes to sites of polarized growth in a cell cycle dependent- and Spa2p-dependent manner, interacts with MAPKKs Mkk1p, Mkk2p, and Ste7p | YLR313C | SPH1 | 6.1 |
| Putative glycoside hydrolase of the spore wall envelope; required for normal spore wall assembly, possibly for cross-linking between the glucan and chitosan layers; expressed during sporulation | YLR213C | CRR1 | 3.5 |
| G-protein coupled |  |  |  |
| Mating pheromone a-factor, made by a cells; interacts with alpha cells to induce cell cycle arrest and other responses leading to mating; biogenesis involves C-terminal modification, N-terminal proteolysis, and export; also encoded by MFA2 | YDR461W | MFA1 | 8.7 |
| Receptor for alpha-factor pheromone; seven transmembrane-domain GPCR that interacts with both pheromone and a heterotrimeric G protein to initiate the signaling response that leads to mating between haploid a and alpha cells | YFL026W | STE2 | 7.1 |
| Mating pheromone alpha-factor, made by alpha cells; interacts with mating type a cells to induce cell cycle arrest and other responses leading to mating; also encoded by MF(ALPHA)2, although MF(ALPHA)1 produces most alpha-factor | YPL187W | MF(ALPHA)1 | 4.8 |
| Negative regulator of glucose-induced cAMP signaling; directly activates the GTPase activity of the heterotrimeric G protein alpha subunit Gpa2p | YOR107W | RGS2 | 5.6 |
| Pyramidine nucleotide biosynthetic process |  |  |  |
| Dihydroorotate dehydrogenase, catalyzes the fourth enzymatic step in the de novo biosynthesis of pyrimidines, converting dihydroorotic acid into orotic acid | YKL216W | URA1 | 37.6 |
| RNA modification process |  |  |  |
| Essential protein required for the accumulation of box C/D snoRNA | YHR040W | BCD1 | 6.9 |
| Protein of unknown function, contains a J-domain, which is a region with homology to the E. coli DnaJ protein | YJR097W | JJJ3 | 5.7 |
| Dihydrouridine synthase, member of a widespread family of conserved proteins including Smm1p, Dus1p, and Dus4p; contains a consensus oleate response element (ORE) in its promoter region | YLR401C | DUS3 | 8.1 |
| Protein that plays a role, with Elongator complex, in modification of wobble nucleosides in tRNA; involved in sensitivity to G1 arrest induced by zymocin; interacts with chromatin throughout the genome; also interacts with Cdc19p | YKL110C | KTI12 | 6.2 |
| Protein required for synthesis of diphthamide, a modified histidine residue of translation elongation factor 2; functions with Dph1p, Dph2p, Jjj3p, and Dph5p; required, with Elongator complex, for modification of wobble nucleosides in tRNA | YBL071W-A | KTI11 | 5.3 |
| Mitochondrial protein, forms a heterodimer complex with Mss1p that performs the 5-carboxymethylaminomethyl modification of the wobble uridine base in mitochondrial tRNAs; required for respiration in paromomycin-resistant 15S rRNA mutants | YGL236C | MTO1 | 6.6 |
| DRAP deaminase, catalyzes the third step of the riboflavin biosynthesis pathway; cytoplasmic tRNA pseudouridine synthase involved in pseudouridylation of cytoplasmic tRNAs at position 32 | YOL066C | RIB2 | 9.6 |
