# Supplementary material for: Agp2, a Member of the Yeast Amino Acid Permease Family, Positively Regulates Polyamine Transport at the Transcriptional Level
Source: PLoS One. 2013 Jun 3;8(6):e65717. doi: 10.1371/journal.pone.0065717 (PMC3670898; doi:10.1371/journal.pone.0065717)
Supplement: Table S4 — Functional category of upregulated genes in the absence of Agp2. (DOCX) [file pone.0065717.s005.docx]

**Table S4:** **Functional category of upregulated genes in the absence of Agp2**

| Function | ORF | Gene Name | Fold change |
| --- | --- | --- | --- |
| Cofactor and coenzyme metabolic process |  |  |  |
| Citrate synthase, catalyzes the condensation of acetyl coenzyme A and oxaloacetate to form citrate, mitochondrial isozyme involved in the TCA cycle | YPR001W | CIT3 | 12.3 |
| Glucose-repressible alcohol dehydrogenase II, catalyzes the conversion of ethanol to acetaldehyde; involved in the production of certain carboxylate esters; regulated by ADR1 | YMR303C | ADH2 | 15.5 |
| Mitochondrial external NADH dehydrogenase, catalyzes the oxidation of cytosolic NADH; Nde1p and Nde2p are involved in providing the cytosolic NADH to the mitochondrial respiratory chain | YDL085W | NDE2 | 7.6 |
| Minor succinate dehydrogenase isozyme; homologous to Sdh1p, the major isozyme reponsible for the oxidation of succinate and transfer of electrons to ubiquinone; induced during the diauxic shift in a Cat8p-dependent manner |  | --- |  |
| Mitochondrial inner membrane of unknown function; similar to Tim18p and Sdh4p; expression induced by nitrogen limitation in a GLN3, GAT1-dependent manner |  | --- |  |
| Protein of unknown function with similarity to succinate dehydrogenase cytochrome b subunit; YMR118C is not an essential gene |  | --- |  |
| Kynureninase, required for biosynthesis of nicotinic acid from tryptophan via kynurenine pathway | YLR231C | BNA5 | 9.3 |
| Ferrochelatase, a mitochondrial inner membrane protein, catalyzes the insertion of ferrous iron into protoporphyrin IX, the eighth and final step in the heme biosynthetic pathway | YOR176W | HEM15 | 8.6 |
| 3-hydroxyanthranilic acid dioxygenase, required for biosynthesis of nicotinic acid from tryptophan via kynurenine pathway | YJR025C | BNA1 | 8.1 |
| Acetyl-coA synthetase isoform which, along with Acs2p, is the nuclear source of acetyl-coA for histone acetlyation; expressed during growth on nonfermentable carbon sources and under aerobic conditions | YAL054C | ACS1 | 7.4 |
| Transketolase, similar to Tkl1p; catalyzes conversion of xylulose-5-phosphate and ribose-5-phosphate to sedoheptulose-7-phosphate and glyceraldehyde-3-phosphate in the pentose phosphate pathway; needed for synthesis of aromatic amino acids | YBR117C | TKL2 | 3 |
| One of two isozymes of HMG-CoA reductase that catalyzes the conversion of HMG-CoA to mevalonate, which is a rate-limiting step in sterol biosynthesis; localizes to the nuclear envelope; overproduction induces the formation of karmellae | YML075C | HMG1 | 6.6 |
| Reproductive Cellular Process |  |  |  |
| Protein required for spore wall maturation; expressed during sporulation; may be a component of the spore wall | YHR139C | SPS100 | 5.8 |
| Spore-specific water channel that mediates the transport of water across cell membranes, developmentally controlled; may play a role in spore maturation, probably by allowing water outflow, may be involved in freeze tolerance | YPR192W | AQY1 | 6.3 |
| Substrate of the Hub1p ubiquitin-like protein that localizes to the shmoo tip (mating projection); mutants are defective for mating projection formation, thereby implicating Hbt1p in polarized cell morphogenesis | YDL223C | HBT1 | 10 |
| Integral membrane protein localized to mitochondria (untagged protein) and eisosomes, immobile patches at the cortex associated with endocytosis; sporulation and sphingolipid content are altered in mutants; has homologs SUR7 and YNL194C | YDL222C | FMP45 | 4.7 |
| Meiosis-specific regulatory subunit of the Glc7p protein phosphatase, regulates spore wall formation and septin organization, required for expression of some late meiotic genes and for normal localization of Glc7p | YBR045C | GIP1 | 8.4 |
| Sporulation-specific homolog of the yeast CDC3/10/11/12 family of bud neck microfilament genes; septin protein involved in sporulation; regulated by ABFI | YGR059W | SPR3 | 7.1 |
| G1 cyclin involved in regulation of the cell cycle; activates Cdc28p kinase to promote the G1 to S phase transition; late G1 specific expression depends on transcription factor complexes, MBF (Swi6p-Mbp1p) and SBF (Swi6p-Swi4p) | YPL256C | CLN2 | 8.6 |
| Protein of unknown function involved in the assembly of the prospore membrane during sporulation | YPL027W | SMA1 | 4.3 |
| Hypothetical protein |  | --- | 4.3 |
| Protein involved in sporulation; required for the construction of the outer spore wall layers; required for proper localization of Spo14p | YOR255W | OSW1 | 2.4 |
| Plasma membrane protein involved in G-protein mediated pheromone signaling pathway; overproduction suppresses bem1 mutations | YNL173C | MDG1 | 5.9 |
| Site-specific endonuclease required for gene conversion at the MAT locus (homothallic switching) through the generation of a ds DNA break; expression restricted to mother cells in late G1 as controlled by Swi4p-Swi6p, Swi5p and Ash1p | YBR117C | TKL2 | 2.8 |
| Peroxisomal 2,4-dienoyl-CoA reductase, auxiliary enzyme of fatty acid beta-oxidation; homodimeric enzyme required for growth and sporulation on petroselineate medium; expression induced during late sporulation and in the presence of oleate | YML075C | HMG1 | 6.6 |
| Lipid metabolism |  |  |  |
| Fatty-acyl coenzyme A oxidase, involved in the fatty acid beta-oxidation pathway; localized to the peroxisomal matrix | YGL205W | POX1 | 9.2 |
| Fatty acid desaturase, required for monounsaturated fatty acid synthesis and for normal distribution of mitochondria | YGL055W | OLE1 | 19.4 |
| Peroxisomal delta3,delta2-enoyl-CoA isomerase, hexameric protein that converts 3-hexenoyl-CoA to trans-2-hexenoyl-CoA, essential for the beta-oxidation of unsaturated fatty acids, oleate-induced | YLR284C | ECI1 | 10.2 |
| ER membrane protein involved in regulation of OLE1 transcription, acts with homolog Spt23p; inactive ER form dimerizes and one subunit is then activated by ubiquitin/proteasome-dependent processing followed by nuclear targeting | YIR033W | MGA2 | 6.9 |
| 2-enoyl thioester reductase, member of the medium chain dehydrogenase/reductase family; localized to in mitochondria, where it has a probable role in fatty acid synthesis | YBR026C | ETR1 | 5.2 |
| Peroxisomal adenine nucleotide transporter; involved in beta-oxidation of medium-chain fatty acid; required for peroxisome proliferation | YPR128C | ANT1 | 8.3 |
| Alpha subunit of fatty acid synthetase, which catalyzes the synthesis of long-chain saturated fatty acids; contains beta-ketoacyl reductase and beta-ketoacyl synthase activities | YPL231W | FAS2 | 9.6 |
| Peroxisomal 2,4-dienoyl-CoA reductase, auxiliary enzyme of fatty acid beta-oxidation; homodimeric enzyme required for growth and sporulation on petroselineate medium; expression induced during late sporulation and in the presence of oleate | YNL202W | SPS19 | 5.2 |
| Stress response |  |  |  |
| Protein with similarity to cyclin-dependent kinase inhibitors, overproduction suppresses a plc1 null mutation; green fluorescent protein (GFP)-fusion protein localizes to the cytoplasm in a punctate pattern | YHR136C | SPL2 | 12 |
| Minor succinate dehydrogenase isozyme; homologous to Sdh1p, the major isozyme reponsible for the oxidation of succinate and transfer of electrons to ubiquinone; induced during the diauxic shift in a Cat8p-dependent manner |  | --- |  |
| Substrate of the Hub1p ubiquitin-like protein that localizes to the shmoo tip (mating projection); mutants are defective for mating projection formation, thereby implicating Hbt1p in polarized cell morphogenesis | YDL223C | HBT1 | 10 |
| Meiosis specific protein involved in DMC1-dependent meiotic recombination, forms heterodimer with Mei5p; proposed to be an assembly factor for Dmc1p | YHR079C-A | SAE3 | 4.4 |
| Cytosolic catalase T, has a role in protection from oxidative damage by hydrogen peroxide | YGR088W | CTT1 | 7.2 |
| Hydrophilin of unknown function; stress induced (osmotic, ionic, oxidative, heat shock and heavy metals); regulated by the HOG pathway | YPL223C | GRE1 | 3.4 |
| DNA binding protein with possible chromatin-reorganizing activity involved in transcriptional activation, gene silencing, and DNA replication and repair | YKL112W | ABF1 | 11.2 |
| Peroxisomal delta3,delta2-enoyl-CoA isomerase, hexameric protein that converts 3-hexenoyl-CoA to trans-2-hexenoyl-CoA, essential for the beta-oxidation of unsaturated fatty acids, oleate-induced | YLR284C | ECI1 | 10.2 |
| Meiosis specific protein involved in DMC1-dependent meiotic recombination, forms heterodimer with Sae3p; proposed to be an assembly factor for Dmc1p | YPL121C | MEI5 | 5.9 |
| ER membrane protein involved in regulation of OLE1 transcription, acts with homolog Spt23p; inactive ER form dimerizes and one subunit is then activated by ubiquitin/proteasome-dependent processing followed by nuclear targeting | YIR033W | MGA2 | 6.9 |
| ATPase component of the RSC chromatin remodeling complex; required for expression of early meiotic genes; essential helicase-related protein homologous to Snf2p | YIL126W | STH1 | 6.6 |
| Transketolase, similar to Tkl1p; catalyzes conversion of xylulose-5-phosphate and ribose-5-phosphate to sedoheptulose-7-phosphate and glyceraldehyde-3-phosphate in the pentose phosphate pathway; needed for synthesis of aromatic amino acids | YBR117C | TKL2 | 2.8 |
| Putative medium-chain alcohol dehydrogenase with similarity to BDH1; transcription induced by constitutively active PDR1 and PDR3; BDH2 is an essential gene | YAL061W | BDH2 | 6.6 |
| Protein required for accurate chromosome segregation during meiosis | YIL132C | CSM2 | 4.9 |
| Regulation of transcription |  |  |  |
| Transcriptional corepressor involved in the regulation of ribosomal protein gene transcription via the TOR signaling pathway and protein kinase A, phosphorylated by activated Yak1p which promotes accumulation of Crf1p in the nucleus | YDR223W | CRF1 | 5.3 |
| Protein kinase with a possible role in MAP kinase signaling in the pheromone response pathway | YDL214C | PRR2 | 7.5 |
| DNA binding protein with possible chromatin-reorganizing activity involved in transcriptional activation, gene silencing, and DNA replication and repair | YKL112W | ABF1 | 11.2 |
| Transcription factor that activates transcription of genes expressed at the M/G1 phase boundary and in G1 phase; localization to the nucleus occurs during G1 and appears to be regulated by phosphorylation by Cdc28p kinase | YDR146C | SWI5 | 6.6 |
| ER membrane protein involved in regulation of OLE1 transcription, acts with homolog Spt23p; inactive ER form dimerizes and one subunit is then activated by ubiquitin/proteasome-dependent processing followed by nuclear targeting | YIR033W | MGA2 | 6.9 |
| Essential chromatin-associated protein involved in the initiation of DNA replication; required for the association of the MCM2-7 complex with replication origins | YIL150C | MCM10 | 5.4 |
| ATPase component of the RSC chromatin remodeling complex; required for expression of early meiotic genes; essential helicase-related protein homologous to Snf2p | YIL126W | STH1 | 6.6 |
| Transcriptional repressor that binds to promoter sequences of the cyclin genes, CYS3, and SMF2; expression is induced by stress or starvation during mitosis, and late in meiosis; member of the Swi4p/Mbp1p family; potential Cdc28p substrate | YIL101C | XBP1 | 6.5 |
| Protein similar to heat shock transcription factor; multicopy suppressor of pseudohyphal growth defects of ammonium permease mutants | YGR249W | MGA1 | 3.9 |
| Hypothetical protein | --- |  |  |
| Zinc finger transcription factor involved in the complex regulation of gene expression in response to levels of heme and oxygen; the S288C sequence differs from other strain backgrounds due to a Ty1 insertion in the carboxy terminus | YLR256W | HAP1 | 4.7 |
| Zinc finger protein involved in control of meiosis; prevents meiosis by repressing IME1 expression and promotes mitosis by activating CLN2 expression; directly repressed by a1-a2 regulator; mediates cell type control of sporulation | YGR044C | RME1 | 6.2 |
| Cell cycle processes |  |  |  |
| Daughter cell-specific protein, may participate in pathways regulating cell wall metabolism; deletion affects cell separation after division and sensitivity to drugs targeted against the cell wall | YER124C | DSE1 | 11.4 |
| Glycosylphosphatidylinositol (GPI)-anchored cell wall endoglucanase required for proper cell separation after cytokinesis, expression is activated by Swi5p and tightly regulated in a cell cycle-dependent manner | YNL327W | EGT2 | 11.5 |
| Daughter cell-specific protein, may help establish daughter fate | YOR264W | DSE3 | 7.7 |
| G1 cyclin involved in regulation of the cell cycle; activates Cdc28p kinase to promote the G1 to S phase transition; late G1 specific expression depends on transcription factor complexes, MBF (Swi6p-Mbp1p) and SBF (Swi6p-Swi4p) | YPL256C | CLN2 | 8.6 |
| Essential chromatin-associated protein involved in the initiation of DNA replication; required for the association of the MCM2-7 complex with replication origins | YIL150C | MCM10 | 5.4 |
| ATPase component of the RSC chromatin remodeling complex; required for expression of early meiotic genes; essential helicase-related protein homologous to Snf2p | YIL126W | STH1 | 6.6 |
| Zinc finger protein involved in control of meiosis; prevents meiosis by repressing IME1 expression and promotes mitosis by activating CLN2 expression; directly repressed by a1-a2 regulator; mediates cell type control of sporulation | YGR044C | RME1 | 6.2 |
| B-type cyclin involved in cell cycle progression; activates Cdc28p to promote the transition from G2 to M phase; accumulates during G2 and M, then targeted via a destruction box motif for ubiquitin-mediated degradation by the proteasome | YGR108W | CLB1 | 5.6 |
| Nucleotide biosynthetic process |  |  |  |
| Plasma membrane H+-ATPase, isoform of Pma1p, involved in pumping protons out of the cell; regulator of cytoplasmic pH and plasma membrane potential | YPL036W | PMA2 | 5.1 |
| Kynureninase, required for biosynthesis of nicotinic acid from tryptophan via kynurenine pathway | YLR231C | BNA5 | 9.3 |
| 3-hydroxyanthranilic acid dioxygenase, required for biosynth  esis of nicotinic acid from tryptophan via kynurenine pathway | YJR025C | BNA1 | 8.1 |
| Protein localization and transport |  |  |  |
| O-glycosylated protein required for cell wall stability; attached to the cell wall via beta-1,3-glucan; mediates mitochondrial translocation of Apn1p; expression regulated by the cell integrity pathway and by Swi5p during the cell cycle | YKL164C | PIR1 | 9.4 |
| Oligopeptide transporter; member of the OPT family, with potential orthologs in S. pombe and C. albicans | YPR194C | OPT2 | 6.1 |
| GTPase, similar to Ypt51p and Ypt52p and to mammalian rab5; required for vacuolar protein sorting and endocytosis | YNL093W | YPT53 | 7.3 |
| Protein that interacts with the Atg12p-Atg5p conjugate during formation of the pre-autophagosomal structure; essential for autophagy | YMR159C | ATG16 | 5.3 |
| One of two isozymes of HMG-CoA reductase that catalyzes the conversion of HMG-CoA to mevalonate, which is a rate-limiting step in sterol biosynthesis; localizes to the nuclear envelope; overproduction induces the formation of karmellae | YML075C | HMG1 | 6.6 |
| Protein containing an N-terminal epsin-like domain involved in clathrin recruitment and traffic between the Golgi and endosomes; associates with the clathrin adaptor Gga2p, clathrin adaptor complex AP-1, and clathrin | YDR153C | ENT5 | 5.6 |
| Peptidase activity |  |  |  |
| Retrotransposon TYA Gag and TYB Pol genes; in YARCTY1-1 TYB is mutant and probably non-functional /// Retrotransposon TYA Gag and TYB Pol genes; transcribed/translated as one unit; polyprotein is processed to make a nucleocapsid-like protein (Gag), reverse transcriptase (RT), protease (PR), and integrase (IN); similar to retroviral genes |  | --- |  |
| Catalytic subunit of the COP9 signalosome (CSN) complex that acts as an isopeptidase in cleaving the ubiquitin-like protein Nedd8 from SCF ubiquitin ligases; metalloendopeptidase involved in the adaptation to pheromone signaling | YDL216C | RRI1 | 7.9 |
| Cysteine aminopeptidase with homocysteine-thiolactonase activity; protects cells against homocysteine toxicity; has bleomycin hydrolase activity in vitro; transcription is regulated by galactose via Gal4p; orthologous to human BLMH | YNL239W | LAP3 | 7.4 |
| Protein catabolic process |  |  |  |
| Substrate of the Hub1p ubiquitin-like protein that localizes to the shmoo tip (mating projection); mutants are defective for mating projection formation, thereby implicating Hbt1p in polarized cell morphogenesis | YDL223C | HBT1 | 10 |
| Cytosolic catalase T, has a role in protection from oxidative damage by hydrogen peroxide | YGR088W | CTT1 | 7.2 |
| Cytoplasmic proteinase A inhibitor, dependent on Pbs2p and Hog1p protein kinases for osmotic induction; intrinsically unstructured, N-terminal half becomes ordered in the active site of proteinase A upon contact | YMR174C | PAI3 | 6.4 |
| Acetyl-coA synthetase isoform which, along with Acs2p, is the nuclear source of acetyl-coA for histone acetlyation; expressed during growth on nonfermentable carbon sources and under aerobic conditions | YAL054C | ACS1 | 7.3 |
| Putative medium-chain alcohol dehydrogenase with similarity to BDH1; transcription induced by constitutively active PDR1 and PDR3; BDH2 is an essential gene | YAL061W | BDH2 | 6.6 |
| Protein with similarity to hect domain E3 ubiquitin-protein ligases, not essential for viability | YJR036C | HUL4 | 8.6 |
| Energy metabolism |  |  |  |
| Plasma membrane H+-ATPase, isoform of Pma1p, involved in pumping protons out of the cell; regulator of cytoplasmic pH and plasma membrane potential | YPL036W | PMA2 | 5.1 |
| Subunit of a heterodimeric peroxisomal ATP-binding cassette transporter complex (Pxa1p-Pxa2p), required for import of long-chain fatty acids into peroxisomes; similarity to human adrenoleukodystrophy transporter and ALD-related proteins | YPL147W | PXA1 | 8.6 |
| Helicase encoded by the Y' element of subtelomeric regions, highly expressed in the mutants lacking the telomerase component TLC1; potentially phosphorylated by Cdc28p /// Putative protein of unknown function | YDR545W | YRF1-1 /// YRF1-2 /// YRF1-3 /// YRF1-4 /// YRF1-5 /// YRF1-6 /// YRF1-7 | 3.4 |
| ATPase component of the RSC chromatin remodeling complex; required for expression of early meiotic genes; essential helicase-related protein homologous to Snf2p | YIL126W | STH1 | 6.6 |
| Essential ATP-dependent RNA helicase of the DEAD-box protein family, involved in nonsense-mediated mRNA decay and rRNA processing | YNL112W | DBP2 | 3.7 |
| Helicase-like protein encoded within the telomeric Y' element /// Helicase encoded by the Y' element of subtelomeric regions, highly expressed in the mutants lacking the telomerase component TLC1; potentially phosphorylated by Cdc28p /// Putative protein of unknown function /// Putative protein of unknown function; similarity to DNA helicases that are encoded within subtelomeric Y' elements and induced in telomerase deficient survivors /// Putative protein of unknown function; similarity to DNA helicases that are also encoded within subtelomeric Y' elements and induced in telomerase deficient survivors /// Putative protein of unknown function with similarity to helicases; YLL066C is not an essential gene /// Putative protein of unknown function with similarity to helicases /// Putative protein of unknown function with similarity to helicases; the authentic, non-tagged protein is detected in highly purified mitochondria in high-throughput studies; YML133C contains an intron | YDR545W | YRF1-1 /// YRF1-2 /// YRF1-3 /// YRF1-5 /// YRF1-6 /// YRF1-7 | 11 |
