# Supplementary material for: Agp2, a Member of the Yeast Amino Acid Permease Family, Positively Regulates Polyamine Transport at the Transcriptional Level
Source: PLoS One. 2013 Jun 3;8(6):e65717. doi: 10.1371/journal.pone.0065717 (PMC3670898; doi:10.1371/journal.pone.0065717)
Supplement: Table S5 — Predicted transcription factors that might signal the downregulation of genes in the absence of Agp2. (DOC) [file pone.0065717.s006.doc]

| **Table S5. Predicted transcription factors that might signal the downregulation of genes in the absence of Agp2** | | | | | | | | | | | |
| --- | --- | --- | --- | --- | --- | --- | --- | --- | --- | --- | --- |
| TF[[1]](#footnote-2) | Total genes (microarray) | Total genes downregulated (≥2.5-fold reduction in expression) | | Downregulation ≥ 6-fold | | Downregulation ≥ 5-fold | | Downregulation ≥ 4-fold | | Downregulation ≥ 3-fold | |
|  | *n*= 5724 | *n*= 140 | | *n*= 10 | | *n*=15 | | *n*=27 | | *n*=81 | |
|  | % | % | ER | % | ER | % | ER | % | ER | % | ER |
| Sfp1 | 36.9 | 56.7 |  | 90.0 |  | 92.9 |  | 68.0 |  | 63.0 |  |
| Ste12 | 34.2 | 51.1 |  | 80.0 |  | 71.4 |  | 56.0 |  | 58.0 |  |
| Yap1 | 29.0 | 41.1 |  | 50.0 |  | 50.0 |  | 52.0 |  | 37.0 |  |
| Rap1 | 23.4 | 31.9 |  | 20.0 |  | 42.9 |  | 44.0 |  | 37.0 |  |
| Gcn4 | 21.4 | 31.9 |  | 40.0 |  | 50.0 |  | 44.0 |  | 30.9 |  |
| Met4 | 20.7 | 25.5 |  | 30.0 |  | 28.6 |  | 28.0 |  | 30.9 |  |
| Msn2 | 19.6 | 36.2 |  | 40.0 |  | 50.0 |  | 48.0 |  | 39.5 |  |
| Aft1 | 17.9 | 32.6 |  | 50.0 |  | 42.9 |  | 32.0 |  | 33.3 |  |
| Sok2 | 16.7 | 39.0 |  | 70.0 |  | 71.4 |  | 56.0 |  | 45.7 |  |
| Rpn4 | 16.4 | 25.5 |  | 50.0 |  | 50.0 |  | 40.0 |  | 30.9 |  |
| Fhl1 | 13.6 | 13.5 |  | 0 |  | 14.3 |  | 16.0 |  | 16.0 |  |
| Arr1 | 12.0 | 17.0 |  | 40.0 |  | 35.7 |  | 24.0 |  | 21.0 |  |
| Msn4 | 11.8 | 24.1 |  | 40.0 |  | 28.6 |  | 32.0 |  | 27.2 |  |
| Abf1 | 11.1 | 11.3 |  | 20.0 |  | 21.4 |  | 12.0 |  | 11.1 |  |
| Ino4 | 10.9 | 17.7 |  | 20.0 |  | 21.4 |  | 16.0 |  | 16.0 |  |
| Gln3 | 10.9 | 17.0 |  | 30.0 |  | 35.7 |  | 28.0 |  | 22.2 |  |
| Sko1 | 10.3 | 21.3 |  | 30.0 |  | 28.6 |  | 20.0 |  | 19.8 |  |
| Skn7 | 10.2 | 19.9 |  | 10.0 |  | 7.1 |  | 16.0 |  | 18.5 |  |
| Gcr2 | 10.1 | 25.5 |  | 10.0 |  | 14.3 |  | 32.0 |  | 28.4 |  |
| Pdr1 | 9.7 | 17.0 |  | 60.0 |  | 50.0 |  | 32.0 |  | 23.5 |  |
| Yap6 | 9.7 | 8.5 |  | 20.0 |  | 14.3 |  | 8.0 |  | 6.2 |  |
| Hsf1 | 9.5 | 12.1 |  | 20.0 |  | 21.4 |  | 24.0 |  | 11.1 |  |
| Tec1 | 9.5 | 20.6 |  | 40.0 |  | 35.7 |  | 28.0 |  | 19.8 |  |
| Swi4 | 9.5 | 18.4 |  | 20.0 |  | 14.3 |  | 16.0 |  | 24.7 |  |
| Cin5 | 9.0 | 9.9 |  | 0 |  | 0 |  | 4.0 |  | 8.6 |  |
| Leu3 | 8.6 | 8.5 |  | 20.0 |  | 14.3 |  | 8.0 |  | 8.6 |  |
| Pdr3 | 8.6 | 15.6 |  | 30.0 |  | 42.9 |  | 28.0 |  | 22.2 |  |
| Reb1p | 8.4 | 9.9 |  | 0 |  | 0 |  | 0 |  | 3.7 |  |
| Rfx1 | 8.3 | 13.5 |  | 30.0 |  | 21.4 |  | 16.0 |  | 12.3 |  |
| Xbp1 | 8.2 | 9.9 |  | 0 |  | 0 |  | 0 |  | 9.9 |  |
| Phd1 | 8.0 | 12.8 |  | 0 |  | 14.3 |  | 12.0 |  | 11.1 |  |
| Cad1 | 7.9 | 9.9 |  | 20.0 |  | 21.4 |  | 24.0 |  | 11.1 |  |
| Mbp1 | 7.6 | 6.4 |  | 20.0 |  | 14.3 |  | 12.0 |  | 7.4 |  |
| Yox1 | 7.4 | 14.2 |  | 50.0 |  | 35.7 |  | 28.0 |  | 18.5 |  |
| Adr1 | 7.4 | 12.8 |  | 20.0 |  | 28.6 |  | 16.0 |  | 16.0 |  |
| Yap5 | 7.2 | 9.9 |  | 0 |  | 7.1 |  | 12.0 |  | 8.6 |  |
| Mcm1 | 6.9 | 13.5 |  | 20.0 |  | 14.3 |  | 16.0 |  | 11.1 |  |
| Hap4 | 6.5 | 14.9 |  | 40.0 |  | 28.6 |  | 20.0 |  | 16.0 |  |
| Pho4 | 6.4 | 9.9 |  | 0 |  | 0 |  | 4.0 |  | 9.9 |  |
| Nrg1 | 6.2 | 15.6 |  | 10.0 |  | 14.3 |  | 12.0 |  | 14.8 |  |
| Rox1 | 6.2 | 19.9 |  | 50.0 |  | 42.9 |  | 28.0 |  | 23.5 |  |
| Ifh1 | 5.7 | 5.0 |  | 10.0 |  | 7.1 |  | 12.0 |  | 6.2 |  |
| Cbf1 | 5.5 | 6.4 |  | 20.0 |  | 14.3 |  | 16.0 |  | 6.2 |  |
| Stb5 | 5.4 | 12.1 |  | 0 |  | 0 |  | 0 |  | 12.3 |  |
| Stp2 | 5.3 | 14.2 |  | 20.0 |  | 14.3 |  | 32.0 |  | 17.3 |  |
| Yhp1 | 5.1 | 17.7 |  | 60.0 |  | 42.9 |  | 32.0 |  | 22.2 |  |
| -----[[2]](#footnote-3) | ----- |  |  |  |  |  |  |  |  |  |  |
| Crz1 | 4.9 | 7.8 |  | 10.0 |  | 14.3 |  | 8.0 |  | 7.4 |  |
| Mga1 | 4.8 | 13.5 |  | 30.0 |  | 28.6 |  | 20.0 |  | 14.8 |  |
| Ecm22 | 4.6 | 9.2 |  | 0 |  | 0 |  | 0 |  | 9.9 |  |
| Oaf1 | 4.4 | 9.9 |  | 0 |  | 0 |  | 0 |  | 7.4 |  |
| Gcr1 | 4.1 | 7.1 |  | 20.0 |  | 14.3 |  | 8.0 |  | 8.6 |  |
| Mig1 | 3.9 | 8.5 |  | 0 |  | 0 |  | 0 |  | 8.6 |  |
| Ume6 | 3.9 | 8.5 |  | 0 |  | 0 |  | 0 |  | 8.6 |  |
| Gis1 | 3.8 | 9.2 |  | 10.0 |  | 7.1 |  | 12.0 |  | 13.6 |  |
| Rme1 | 3.8 | 10.6 |  | 40.0 |  | 35.7 |  | 20.0 |  | 12.3 |  |
| Dal81 | 3.7 | 6.4 |  | 0 |  | 0 |  | 12.0 |  | 7.4 |  |
| Rtg3 | 3.5 | 7.1 |  | 0 |  | 14.3 |  | 16.0 |  | 8.6 |  |
| Rim101 | 3.4 | 8.5 |  | 0 |  | 0 |  | 0 |  | 7.4 |  |
| Hms1 | 3.3 | 7.1 |  | 40.0 |  | 28.6 |  | 16.0 |  | 8.6 |  |
| Cst6 | 3.2 | 13.5 |  | 20.0 |  | 21.4 |  | 16.0 |  | 14.8 |  |
| Upc2 | 3.2 | 7.1 |  | 20.0 |  | 21.4 |  | 20.0 |  | 9.9 |  |
| Ino2 | 2.7 | 10.6 |  | 20.0 |  | 14.3 |  | 12.0 |  | 9.9 |  |
| Gzf3 | 2.2 | 7.8 |  | 20.0 |  | 21.4 |  | 20.0 |  | 11.1 |  |
| Mot3 | 2.2 | 9.2 |  | 20.0 |  | 14.3 |  | 12.0 |  | 11.1 |  |
| Azf1 | 2.0 | 7.8 |  | 20.0 |  | 14.3 |  | 8.0 |  | 4.9 |  |
| Rgt1 | 1.0 | 4.3 |  | 0 |  | 0 |  | 0 |  | 7.4 |  |
| Kar4 | 0.4 | 2.1 |  | 0 |  | 7.1 |  | 4.0 |  | 2.5 |  |

TF, transcription factor; ER, % enrichment for TF representation in that subset (ER=100 x[% in subset/% of total arrayed])

Binding of TFs listed below the dotted line is documented for < 5% of total genes screened in the microarray.

1. TF, transcription factor; ER, % enrichment for TF representation in that subset [↑](#footnote-ref-2)
2. Binding of TFs listed below the dotted line is documented for < 5% of total genes screened in the microarray. [↑](#footnote-ref-3)
