# Supplementary material for: Agp2, a Member of the Yeast Amino Acid Permease Family, Positively Regulates Polyamine Transport at the Transcriptional Level
Source: PLoS One. 2013 Jun 3;8(6):e65717. doi: 10.1371/journal.pone.0065717 (PMC3670898; doi:10.1371/journal.pone.0065717)
Supplement: Table S6 — Predicted transcription factors that might signal the upregulation of genes in the absence of Agp2. (DOC) [file pone.0065717.s007.doc]

| **Table S6. Predicted transcription factors that might signal the upregulation of genes in the absence of Agp2** | | | | | | | | | |
| --- | --- | --- | --- | --- | --- | --- | --- | --- | --- |
| TF[[1]](#footnote-2) | Total genes (microarray) | Total genes upregulated (≥2-fold increase in expression) | | Upregulation ≥ 5-fold | | Upregulation ≥ 4-fold | | Upregulation ≥ 3-fold | |
|  | *n*= 5724 | *n*= 152 | | *n*=12 | | *n*=22 | | *n*=41 | |
|  | % | % | ER | % | ER | % | ER | % | ER |
| Sfp1 | 36.9 | 34.7 |  | 25.0 |  | 31.8 |  | 36.6 |  |
| Ste12 | 34.2 | 48.0 |  | 75.0 |  | 68.2 |  | 68.3 |  |
| Yap1 | 29.0 | 40.7 |  | 50.0 |  | 54.5 |  | 58.5 |  |
| Rap1 | 23.4 | 34.7 |  | 25.0 |  | 36.4 |  | 36.6 |  |
| Gcn4 | 21.4 | 23.3 |  | 50.0 |  | 45.5 |  | 39.0 |  |
| Met4 | 20.7 | 22.7 |  | 33.3 |  | 31.8 |  | 29.3 |  |
| Msn2 | 19.6 | 29.3 |  | 41.7 |  | 50.0 |  | 51.2 |  |
| Aft1 | 17.9 | 33.3 |  | 50.0 |  | 40.9 |  | 46.3 |  |
| Sok2 | 16.7 | 32.0 |  | 66.7 |  | 68.2 |  | 53.7 |  |
| Rpn4 | 16.4 | 18.7 |  | 25.0 |  | 27.3 |  | 29.3 |  |
| Fhl1 | 13.6 | 14.0 |  | 16.7 |  | 18.2 |  | 12.2 |  |
| Arr1 | 12.0 | 16.0 |  | 16.7 |  | 18.2 |  | 17.1 |  |
| Msn4 | 11.8 | 18.0 |  | 25.0 |  | 27.3 |  | 39.0 |  |
| Abf1 | 11.1 | 12.0 |  | 25.0 |  | 22.7 |  | 14.6 |  |
| Ino4 | 10.9 | 13.3 |  | 16.7 |  | 13.6 |  | 12.2 |  |
| Gln3 | 10.9 | 7.3 |  | 8.3 |  | 9.1 |  | 7.3 |  |
| Sko1 | 10.3 | 14.0 |  | 33.3 |  | 22.7 |  | 31.7 |  |
| Skn7 | 10.2 | 12.7 |  | 16.7 |  | 18.2 |  | 22.0 |  |
| Gcr2 | 10.1 | 19.3 |  | 25.0 |  | 22.7 |  | 22.0 |  |
| Pdr1 | 9.7 | 18.0 |  | 16.7 |  | 27.3 |  | 34.1 |  |
| Yap6 | 9.7 | 10.0 |  | 8.3 |  | 9.1 |  | 12.2 |  |
| Hsf1 | 9.5 | 14.7 |  | 16.7 |  | 22.7 |  | 29.3 |  |
| Tec1 | 9.5 | 14.0 |  | 16.7 |  | 22.7 |  | 24.4 |  |
| Swi4 | 9.5 | 11.3 |  | 8.3 |  | 13.6 |  | 12.2 |  |
| Cin5 | 9.0 | 10.0 |  | 8.3 |  | 9.1 |  | 12.2 |  |
| Leu3 | 8.6 | 12.7 |  | 16.7 |  | 18.2 |  | 17.1 |  |
| Pdr3 | 8.6 | 14.0 |  | 16.7 |  | 27.3 |  | 29.3 |  |
| Reb1p | 8.4 | 4.0 |  | 0 |  | 0 |  | 2.4 |  |
| Rfx1 | 8.3 | 14.0 |  | 33.3 |  | 18.2 |  | 14.6 |  |
| Xbp1 | 8.2 | 14.7 |  | 25.0 |  | 27.3 |  | 22.0 |  |
| Phd1 | 8.0 | 11.3 |  | 25.0 |  | 13.6 |  | 12.2 |  |
| Cad1 | 7.9 | 14.0 |  | 33.3 |  | 27.3 |  | 17.1 |  |
| Mbp1 | 7.6 | 6.7 |  | 8.3 |  | 9.1 |  | 4.9 |  |
| Yox1 | 7.4 | 9.3 |  | 8.3 |  | 9.1 |  | 12.2 |  |
| Adr1 | 7.4 | 22.0 |  | 50.0 |  | 40.9 |  | 31.7 |  |
| Yap5 | 7.2 | 14.7 |  | 16.7 |  | 27.3 |  | 26.8 |  |
| Mcm1 | 6.9 | 10.7 |  | 16.7 |  | 13.6 |  | 9.8 |  |
| Hap4 | 6.5 | 11.3 |  | 41.7 |  | 31.8 |  | 22.0 |  |
| Pho4 | 6.4 | 10.7 |  | 33.3 |  | 27.3 |  | 22.0 |  |
| Nrg1 | 6.2 | 8.0 |  | 8.3 |  | 9.1 |  | 12.2 |  |
| Rox1 | 6.2 | 15.3 |  | 16.7 |  | 27.3 |  | 31.7 |  |
| Ifh1 | 5.7 | 4.0 |  | 16.7 |  | 13.6 |  | 7.3 |  |
| Cbf1 | 5.5 | 4.7 |  | 16.7 |  | 9.1 |  | 7.3 |  |
| Stb5 | 5.4 | 7.3 |  | 8.3 |  | 18.2 |  | 12.2 |  |
| Stp2 | 5.3 | 11.3 |  | 16.7 |  | 22.7 |  | 19.5 |  |
| Yhp1 | 5.1 | 9.3 |  | 8.3 |  | 9.1 |  | 9.8 |  |
| -----[[2]](#footnote-3) | ----- |  |  |  |  |  |  |  |  |
| Fkh2 | 5.0 | 8.7 |  | 8.3 |  | 4.5 |  | 9.8 |  |
| Crz1 | 4.9 | 8.7 |  | 16.7 |  | 9.1 |  | 12.2 |  |
| Mga1 | 4.8 | 4.7 |  | 0 |  | 4.5 |  | 4.9 |  |
| Ecm22 | 4.6 | 6.0 |  | 8.3 |  | 4.5 |  | 4.9 |  |
| Tos8 | 4.6 | 7.3 |  | 25.0 |  | 22.7 |  | 14.6 |  |
| Oaf1 | 4.4 | 18.0 |  | 41.7 |  | 40.9 |  | 36.6 |  |
| Gcr1 | 4.1 | 5.3 |  | 16.7 |  | 9.1 |  | 4.9 |  |
| Fkh1 | 4.0 | 6.7 |  | 16.7 |  | 9.1 |  | 7.3 |  |
| Rtg1 | 4.0 | 4.0 |  | 16.7 |  | 13.6 |  | 7.3 |  |
| Flo8 | 3.9 | 5.3 |  | 8.3 |  | 9.1 |  | 4.9 |  |
| Mig1 | 3.9 | 3.3 |  | 0 |  | 0 |  | 4.9 |  |
| Ume6 | 3.9 | 10.7 |  | 8.3 |  | 18.2 |  | 14.6 |  |
| Gis1 | 3.8 | 14.7 |  | 41.7 |  | 36.4 |  | 36.6 |  |
| Rme1 | 3.8 | 10.0 |  | 25.0 |  | 22.7 |  | 19.5 |  |
| Dal81 | 3.7 | 3.3 |  | 0 |  | 0 |  | 2.4 |  |
| Stp1 | 3.7 | 6.0 |  | 16.7 |  | 22.7 |  | 14.6 |  |
| Hac1 | 3.5 | 4.0 |  | 8.3 |  | 9.1 |  | 7.3 |  |
| Rtg3 | 3.5 | 4.7 |  | 16.7 |  | 13.6 |  | 7.3 |  |
| Rim101 | 3.4 | 6.0 |  | 0 |  | 13.6 |  | 9.8 |  |
| Swi5 | 3.4 | 8.7 |  | 16.7 |  | 18.2 |  | 12.2 |  |
| Hms1 | 3.3 | 6.0 |  | 8.3 |  | 4.5 |  | 7.3 |  |
| Cst6 | 3.2 | 10.0 |  | 8.3 |  | 18.2 |  | 19.5 |  |
| Upc2 | 3.2 | 5.3 |  | 8.3 |  | 4.5 |  | 7.3 |  |
| Rlm1 | 3.1 | 6.0 |  | 16.7 |  | 13.6 |  | 9.8 |  |
| Smp1 | 3.0 | 6.0 |  | 25.0 |  | 13.6 |  | 9.8 |  |
| Zap1 | 2.8 | 6.7 |  | 8.3 |  | 4.5 |  | 9.8 |  |
| Aft2 | 2.7 | 6.7 |  | 16.7 |  | 9.1 |  | 9.8 |  |
| Ino2 | 2.7 | 5.3 |  | 8.3 |  | 9.1 |  | 9.8 |  |
| Ace2 | 2.6 | 5.3 |  | 16.7 |  | 13.6 |  | 9.8 |  |
| Pho2 | 2.6 | 6.0 |  | 16.7 |  | 18.2 |  | 14.6 |  |
| Bas1 | 2.4 | 3.3 |  | 16.7 |  | 18.2 |  | 12.2 |  |
| Sum1 | 2.4 | 6.0 |  | 0 |  | 0 |  | 2.4 |  |
| Gat3 | 2.3 | 6.7 |  | 8.3 |  | 4.5 |  | 7.3 |  |
| Pip2 | 2.3 | 9.3 |  | 8.3 |  | 9.1 |  | 12.2 |  |
| Gzf3 | 2.2 | 4.0 |  | 8.3 |  | 4.5 |  | 7.3 |  |
| Mot3 | 2.2 | 5.3 |  | 16.7 |  | 9.1 |  | 12.2 |  |
| Met31 | 2.1 | 2.7 |  | 16.7 |  | 13.6 |  | 7.3 |  |
| Azf1 | 2.0 | 5.3 |  | 0 |  | 4.5 |  | 7.3 |  |
| Cat8 | 2.0 | 6.0 |  | 33.3 |  | 36.4 |  | 19.5 |  |
| Gat4 | 2.0 | 9.3 |  | 16.7 |  | 9.1 |  | 17.1 |  |
| Gal4 | 1.9 | 4.0 |  | 0 |  | 0 |  | 7.3 |  |
| Ash1 | 1.8 | 3.3 |  | 8.3 |  | 9.1 |  | 4.9 |  |
| Aro80 | 1.6 | 1.3 |  | 16.7 |  | 9.1 |  | 4.9 |  |
| Rgm1 | 1.5 | 5.3 |  | 8.3 |  | 4.5 |  | 7.3 |  |
| Msn1 | 1.4 | 2.7 |  | 0 |  | 4.5 |  | 7.3 |  |
| Hot1 | 1.3 | 4.0 |  | 8.3 |  | 9.1 |  | 9.8 |  |
| Sut1 | 1.2 | 3.3 |  | 8.3 |  | 4.5 |  | 7.3 |  |
| Cdc14 | 1.1 | 3.3 |  | 16.7 |  | 13.6 |  | 9.8 |  |
| Rgt1 | 1.0 | 1.3 |  | 0 |  | 0 |  | 0 |  |
| Kar4 | 0.4 | 0.7 |  | 0 |  | 0 |  | 0 |  |
| Opi1 | 0.4 | 2.0 |  | 0 |  | 9.1 |  | 4.9 |  |

TF, transcription factor; ER, % enrichment for TF representation in that subset (ER=100 x[% in subset/% of total arrayed])

Binding of TFs listed below the dotted line is documented for < 5% of total genes screened in the microarray.

1. TF, transcription factor; ER, % enrichment for TF representation in that subset [↑](#footnote-ref-2)
2. Binding of TFs listed below the dotted line is documented for < 5% of total genes screened in the microarray. [↑](#footnote-ref-3)
